# Supplementary material for: Epidemiology of pre-cancerous cervical lesion and risk factors among adult women in Tigray, Ethiopia
Source: PLoS One. 2023 Jan 6;18(1):e0280191. doi: 10.1371/journal.pone.0280191 (PMC9821489; doi:10.1371/journal.pone.0280191)
Supplement: Attachment — Submitted filename: PONE-D-20-24267_R2_reviewer comments 08-22.pdf [file pone.0280191.s004.pdf]

PLOS ONE

Epidemiology of Pre-cancerous Cervical Lesion and Risk Factors among Adult Women n Tigray, Ethiopia

--Manuscript Draft--

**Manuscript Number:**	PONE-D-20-24267R2
**Article Type:**	Research Article
**Full Title:**	Epidemiology of Pre-cancerous Cervical Lesion and Risk Factors among Adult Women n Tigray, Ethiopia
**Short Title:**	Epidemiology of Pre-cancerous Cervical Lesion and Risk Factors among Adult Women n Tigray, Ethiopia
**Corresponding Author:**	Gerezgiher Buruh Abera, M.sc<br>Mekelle University<br>MEKELLE, Ethiopia ETHIOPIA
**Keywords:**	epidemiology; cervical; Pre-cancerous lesions
**Abstract:**	Background<br><br>Cervical cancer is a preventable disease if detected early, but remains the second leading cause of mortality among women in low- and middle-income countries. Data on epidemiology and risk factors in these settings are scarce. This study aimed to assess the magnitude of precancerous lesions and risk factors in Tigray region, Ethiopia.<br><br>Methods<br><br>A community-based cross sectional study was used and 900 participants were selected using multistage sampling. Data were collected using an interviewer administered questionnaire and screening with visual inspection with ascetic acid. Multinomial logistic regression analysis was conducted to estimate predictors.<br><br>Results<br><br>Seventy-nine (8.95%) women were positive for pre-cancer lesion and 35 (3.96%) were suspicious for cervical cancer. Divorced or widowed women had risked 2.5 and 4.7 times more likely to be positive and suspicious, respectively, compared to single women (rrr=2.5, 95% CI [1.13, 5.52]); (rrr=4.69, 95% CI [1.00, 21.84]). The risk of having a suspicious result was 68% lower for women with primary education compared to those with no formal education (rrr=0.32, 95% CI [1.00, 21.84]). History of sexually transmitted infection was associated with positive pre-cancer lesion (rrr=1.91, 95% CI [1.11, 3.27]) whereas, being farmer (rrr=4.83, 95% CI [1.44, 16.13]), merchant (rrr=4.85, 95% CI [1.52, 15.46]), bleeding between periods (rrr =3.26, 95% CI [1.32, 8.04]) and pelvic or back pain (rrr =2.79, 95% CI [1.18, 6.58]) were associated with suspicious for cancer.<br><br>Conclusion<br><br>About 8.9% and 4% of the women were positive and suspicious for pre-cancer cervical lesion, respectively. Marital status, education, sexually transmitted infection, bleeding, and pelvic pain were predictors of cervical precancerous lesions.
**Order of Authors:**	Gerezgiher Buruh Abera, M.sc
	Henock G. Yebyo
	Haftamu Hailekiros
	Selam Niguse
	Yibrah Berhe
	Goitom Gigar

Powered by Editorial Manager® and ProduXion Manager® from Aries Systems Corporation

	Tsehaye Asmelash
	Gelila Goba

Response to Reviewers:

Date:February 12 2022
To:PLOS ONE" plosone@plos.org
From:Gerezgiher Buruh Abera" gbamsc2002@gmail.com (PI)

Subject:Point by Point response to editor and reviewer(s).
TitleEpidemiology of Pre-cancerous Cervical Lesion and Risk Factors among Adult Women in Tigray, Ethiopia
ReferencePONE-D-20-24267R1

Dear/Sir/ professors: editor and reviewer:
We apologize for the late response! It is because of the internet lockdown in Tigray, North Ethiopia, due to crises. We are responding through the internet from NGOs that allow to use for publications, international projects and PhD students only. We are very grateful for the consideration of the manuscript. In accordance with the editors and reviewers' valuable comments and recommendations, we have revised the manuscript and we are hereby submitting the revised work for your consideration. Pages referred in this letter are based on the manuscript with track change.

Additional Editor Comments (if provided):
The authors have tried to address some of my comments but the paper still requires a lot of work before a decision can be made. I would like to request the authors to take time to think about the comments raised and try to offer appropriate responses to ALL the comments raised. We can't continue to return the same comments forever. So, the authors should look at the comments returned by reviewer No. 2 as well as myself and endeavor to fully address all the areas that were inadequately addressed. For instance, I raised issues on the grammatical errors which the authors indicated were addressed but to the contrary, the same errors as before still exist in the paper. I had a comment on the formatting of the references and requested the authors to revise them according to the journal's formatting requirements. However, the references were returned with the same errors as before. Besides, the explanation provided for adjusting the sample size by 10% is not sufficient. What is needed is to include a citation that backs up the use of this percentage. This was not done. Here are other additional comments for the authors to consider.

•Response: Sorry for the inconvenience! We were not to refuse comments. To be genuine, I am not competent in English, since it is my second language. In the previous time, we provide it to colleagues in Illinois University, a native English speaker (through the last listed co-author in this manuscript, She is from Chicago), and at that time we received the copy of the language edited version. Now we tried to go through the manuscript to correct grammatical errors as much as possible based on the direction given.
•We accept the comment on the format of the reference and we tried to correct especially reference 15 based on the old version of the paper, but we could not able to Google to recheck the citation style.
•A different contingency are commonly used from 5% to up to 30% (as in trials), based on the study design. In our cross sectional study, VIA screening was used, which is among the factors that could increase nonresponse rate. Now, we had added sample references to our manuscript. Based on previous studies, response rates ranges from 77.1% (24) to 98.2%, (25). This implies a non-response rate of 1.8% to 29.9% Therefore, based on the average of the references; we assume that 10% would enough to maximize the sample size to compensate the non-response rate expected.

1. The paper STILL has many grammatical errors that would benefit from being reviewed by an English Specialist. I raised similar comments earlier but these aspects are not yet fully addressed. For instance, within the abstract, instead of writing, 'Divorced or widowed women had risked 2.5 and 4.7 times more likely to be positive and suspicious, respectively, compared to single women…', the authors should write: 'Divorced or widowed women were 2.5 and 4.7 times more likely to be positive and suspicious… than single women' The expressed: "had risked" is wrongly used. In addition, the use of 'more likely' begs the use of 'than' rather than 'compared to'. Similar statements exist in the abstract and in the main manuscript that should be

Powered by Editorial Manager® and ProduXion Manager® from Aries Systems Corporation

addressed.

•Response - We accept the comments and it is corrected accordingly, as it can be seen in abstract, page 2 and result sections.
Please also fix the following errors/issues in the abstract:
*Expand ‘rrr’ at first mention, and then abbreviate thereafter
*The word ‘In’ in the title should be changed to ‘in’, while ‘Among’ should be written as ‘among’.
*95%CI should be written in expanded form at first mention, and then abbreviate thereafter
*’…68% lower for women with primary education compared to those with no formal education’ – use ‘than’ instead of ‘compared to’.
*’…History of sexually transmitted infection was associated with positive pre-cancer lesion’ – change ‘pre-cancer lesion’ to ‘pre-cancerous cervical lesion’.
*’Seventy-nine (8.95%) women were positive for pre-cancer lesion’ – “pre-cancer lesion” should be written as ‘pre-cancerous cervical lesions’
*’…were associated with suspicious for cancer’ – rewrite as: ‘… were associated with having a suspicious cervical cancer result’
*In the title, the authors refer to ‘risk factors’ but in the conclusion, they refer to ‘predictors’. Do these two terms mean the same?
•Response: We accept the comment and rrr is written in expanded form (relative risk ratio) under the title of “data analysis” at last paragraph. In abstract, we prefer modification, to delete the values of regression including the rrr and represents using summarized statements. This minimizes the presentation of abstract and did not compromise the meaning, since it is already described under the result in detail.
•We accept the comment and the initial letter of ‘In’ ‘and ‘Among’ in the title has been changed to small letters.
•We accept the comment and CI is written in expanded form (confidence interval) under the title of “Factors associated with cervical screening VIA result” at first paragraph of page 12.
• We accept the comment and the ‘compared to’ has been change to “than” as can be seen under the title of “Factors associated with cervical screening VIA result” at page 12, and under abstract.
•We accept the comment and the word “pre-cancerous cervical lesion’ is used in the whole manuscript to replace all miss spelled “cervical precancerous lesion.
•We accept the comment and the meaning of ‘risk factors’ (something that contributes or indicates to illness: a features or habits, or personal history that increases the probability of disease) and ‘predictors’ (something explains what is going to happen in the future, often on the basis of present). We corrected as ‘risk factors’ as it can be seen in the conclusion section of the abstract and result titles.

2. In the abstract, the expression: ‘A community-based cross sectional study was used…’ should be written as: ‘This was a community-based, cross-sectional study conducted among 900 women in Tigray region, Ethiopia, from --- to ----‘. In other words, the opening statement on the ‘Methods’ sub-section should tell the reader what the study design was, where the study was conducted, and when the study was done. Please note the use of the ‘comma’ after the word ‘community-based’ and the ‘hyphen’ inserted in the words ‘cross sectional’ to turn them into one word.

•Response: We accept the comment and corrected accordingly, as it can be seen in the abstract

3. General formatting requirements. The authors should ensure that they follow the general formatting requirements of the journal. For instance, the formatting for the sections indicates how the headings and sub-headings should be formatted, etc. For instance, under the ‘Methods’ section (the authors write it as ‘Method’), this should be formatted as:

Materials and Methods (Level 1: bold type; 18pt font)
xxx

Study setting and design (Level 2: for sub-sections of major sections; Bold type, 16pt font)

Powered by Editorial Manager® and ProduXion Manager® from Aries Systems Corporation

•Response: We accept the comment and the titles and subtitles has been formatted as level 1 and level 2 respectively and Method has been changed to Materials and Methods.

4. Other corrections
*Use of abbreviations: Please ensure that ALL abbreviations are presented in expanded form at first mention in the abstract or body of the manuscript. Even if these abbreviations have been listed in the list of abbreviations at the end of the paper, this does not take away the need to expand all abbreviations at first mention.
•Response: We accept the comments, and tried to correct accordingly through the whole manuscript.

*Line 153, page 7: 'Data was analysed…' Data is a plural word; so, revise as: 'Data were analysed…'
*Lines 163-164, page 8: the authors write: '…ethicalclearance was obtained from mekelle university, collage of health sience research and community service commiyyee'. The word 'ethicalclearance' should be written as two words (ethical clearance). Names of places should always begin with a capital letter, e.g. Mekelle University… The word 'sience' should be edited to 'Sciences'. This also applies to 'collage' which should be written as 'College'. The word 'commiyyee' does not exist in English. I think the authors meant to write: 'committee'. In general, the entire paper should be edited to address any other areas that are not included in this report. The entire sentence should be revised as follows:
'… ethical clearance was obtained from Mekelle University College of Health Sciences Research and Community Service Committee …'

•Response: We accept the comments, and it is corrected accordingly under the 'data analysis and ethical consideration sub sections.

*Please note that should include the study protocol clearance numbers (e.g. Protocol#: 001/2019') as per your country's national research management guidelines.
•Response: We accept the comments, and the registration number has been included in the manuscript, under "Ethical consideration sub section.

*Ensure consistency in writing the word 'pre-cancerous'. In some sections of the paper, the word is written as 'precancerous' while in others, it is written as 'pre-cancerous'. Please choose one form and maintain it throughout the paper.
•Response: We accept the comments, and we tried to correct throughout the manuscript accordingly.

*Check ALL the references used against the journal's referencing guidelines. I can see that some journal names are italicized while others are not; I can also see that the referenced paper's title ends with a comma instead of a full stop. Other references have the journal name written in full. Please check the journal's referencing style (https://journals.plos.org/plosone/s/submission-guidelines) and fix all the errors in the reference section. When you click on the link, scroll down and look for 'references'. There is a table that shows you how different references should be written.

•Response: We accept the comments. As per our understanding, some journals specify citation preference with some abbreviations (example – BMC Res Notes is for BMC research Notes e.tc.), while others not, for those journals we took as it is. Because we may miss spell it when we abbreviate ourselves. The big challenge is that I am now in Tigray, North Ethiopia where the crisis found and all way of communication are locked. I am responding this Email through NGOs, which allow us to use internet for project and PhD publication only. I am sorry to say that I could not get internet access to either research the journals or to contact my most co-authors to recheck the reference (since they are living abroad). Anyway, I had tried to address some errors as I could.

*Reference 15 reads: 'Dye D et al., 2009) Dye D., Solomon B., Claire H., Yared T., Vanessa H., Teshome D., 384 Marion B., Anne R., (2009) Complex care systems in developing countries. Breast 385 Cancer Patient Navigation in Ethiopia 116:577–85.)' This is an example of bad referencing style.

Powered by Editorial Manager® and ProduXion Manager® from Aries Systems Corporation

•Response: We accept the comments, and we tried to correct reference 15 in the revised manuscript by referring the old version.

In general, please ensure that the references are well formatted based on the journal style.
Please note that:
- Where there are more than six authors, please cite three of them followed by the word 'et al.'
- Where there are six authors or less, list all the authors.
- A reference cannot have two years of publication in the same reference, as shown in the example above
- The year of publication should come at the end, after the journal's name
- PLoS ONE does not use the author version (Anne R., or Marion B., ...). Click on the link above for guidance on how to write authors' names in the references section.
- PLoS ONE uses abbreviated journal names. Please ensure that the journal names are abbreviated but not italicized.
•Response: We accept the comments. For the name of authors in the references, it is difficult to differentiate the surname to be abbreviated, example - Amos D. Mwaka. We understand D. could be the first name. Therefore, when we use initial letter of last name first, we could mistake the first name rather than last name; for that, we prefer to use as it has been published in the journals.

5. Supplementary information. The journal requires that: "Authors can submit essential supporting files and multimedia files along with their manuscripts. All supporting information will be subject to peer review. All file types can be submitted, but files must be smaller than 20 MB in size." I did not see any supplementary information provided. Please click on the link above and check for 'supplementary information'. Here are examples of supplementary information that should be included:
*Data collection tools
*Dataset used during the analysis
* Please check the journal's guidelines on how supplementary information should be formatted.
•Response: We accept the comments, and we had uploaded the dataset in excel used during the analysis. There is no multimedia file which did not incorporated in the analysis and all of the relevant information is included in the manuscript. Data uploaded in the form of tables are used to analyze and develop the manuscript. For the confidentiality issue, we did not upload filled questionnaires.
6. Additional information requested at submission: All manuscripts should carry the following sub-section (please click on the link above for guidance on how to include this sub-section):
- Authors' contributions
[Note: HTML markup is below. Please do not edit.]
•Response: We accept the comments. Since it is short time allowed to use internet, we could not check the link to recheck requirements, but we used the link when we were preparing the manuscript for PLoS ONE. We understand that declaration is important and we added the declaration section as additional information including Authors' contributions and others at the end of this manuscript above acknowledgement sub section.
Reviewers' comments:
Reviewer's Responses to Questions
Comments to the Author

1. If the authors have adequately addressed your comments raised in a previous round of review and you feel that this manuscript is now acceptable for publication, you may indicate that here to bypass the "Comments to the Author" section, enter your conflict of interest statement in the "Confidential to Editor" section, and submit your "Accept" recommendation.
Reviewer #1: All comments have been addressed
Reviewer #2: (No Response)

____
2. Is the manuscript technically sound, and do the data support the conclusions?

The manuscript must describe a technically sound piece of scientific research with data that supports the conclusions. Experiments must have been conducted rigorously, with

appropriate controls, replication, and sample sizes. The conclusions must be drawn appropriately based on the data presented.
Reviewer #1: Yes
Reviewer #2: No

____

3. Has the statistical analysis been performed appropriately and rigorously?
Reviewer #1: Yes
Reviewer #2: No

____

4. Have the authors made all data underlying the findings in their manuscript fully available?

The PLOS Data policy requires authors to make all data underlying the findings described in their manuscript fully available without restriction, with rare exception (please refer to the Data Availability Statement in the manuscript PDF file). The data should be provided as part of the manuscript or its supporting information, or deposited to a public repository. For example, in addition to summary statistics, the data points behind means, medians and variance measures should be available. If there are restrictions on publicly sharing data—e.g. participant privacy or use of data from a third party—those must be specified.
Reviewer #1: Yes
Reviewer #2: No

____

5. Is the manuscript presented in an intelligible fashion and written in standard English?

PLOS ONE does not copyedit accepted manuscripts, so the language in submitted articles must be clear, correct, and unambiguous. Any typographical or grammatical errors should be corrected at revision, so please note any specific errors here.
Reviewer #1: Yes
Reviewer #2: No

____

6. Review Comments to the Author

Please use the space provided to explain your answers to the questions above. You may also include additional comments for the author, including concerns about dual publication, research ethics, or publication ethics. (Please upload your review as an attachment if it exceeds 20,000 characters)
Reviewer #1: (No Response)
Reviewer #2: Summary: The comments have been incompletely addressed, and there are still numerous spelling/grammatical errors.

Please find below specific comments for each section.
Data availability:
1. Previous comment: The authors state that the data are fully available and included in the manuscript. Is there an appendix with the individual patient data, or where can they be found?
Author reply: only data in table 1, 2, and 3 were collected in this paper. Except the identifiers (like ID), there is no additional information collected than tables 1, 2, and 3. If the format of the empty questionnaire is needed, we can upload when requested.
Comment: Data in table 1, 2, and 3 are aggregate data, not individual patient data. It is not possible to replicate the analyses with these data. Can the dataset with the individual patient data be access somewhere? If not, the data availability statement needs to be addressed accordingly.
•As per your good recommendation, the data availability sub section had been added in the manuscript under declaration section, describing all data are included in the manuscript.
Abstract:
1. Previous comment: The abbreviation rrr should be introduced.
Author reply: The abbreviation rrr has been introduced in the “list of abbreviations” section.
Comment: All abbreviations should be introduced in the manuscript text, not only in the list of abbreviations.
•Response: We accept the comment and rrr is written in expanded form (relative risk

Powered by Editorial Manager® and ProduXion Manager® from Aries Systems Corporation

ratio) under the title of “data analysis” at last paragraph in its first use. In abstract, since the result section is better to be summary of the findings, we prefer to summarize and to delete the values of regression including the rrr. This could not change the meaning of the results.

2. Previous comment: In the conclusion, please state the implications of your findings.
Author reply: We accept the comment and correction is made accordingly.
Comment: What changes were made? I don’t see any in the conclusion.

•Response: We accept the comment and implications for the prevalence and risk factors has been specified as seen under conclusion section.

Introduction:
1. Previous comment: Page 3, line 54: Which host factors are the authors referring to?
Author reply: We accept the comment and we refer the participants’ factor as the host factors.
Comment: It is not enough to refer to participants’ factors instead of host factors. Which participant factors may be relevant?
•Response: We understand your concern and we are referring to the life style (habits) of participants like risk taking behavior, substance abuse, alcohol use e.tc.
Methods:
1. Previous comment: Page 5, line 102: Why did the authors assume a cervical precancer prevalence of 50% for the sample size calculation when a previous institution-based study had found a prevalence of 6.7% in the Tigray region?
Author reply: In Ethiopia, VIA screening was not available for non HIV women until 2015. It was only three hospitals providing VIA screening for HIV patents in the study area, Tigray. Therefore the above mentioned prevalence is for HIV patients. Moreover, the above prevalence would provide us less number of participants and it is advisable to increases sample size if affordable. Therefore, we assume increasing participants would improve the representativeness of the eligible participants.
Comment: I do not understand this reply. Women living with HIV are at higher risk of developing precancerous cervical lesions. If the estimate of 6.7% stems from a study among women living with HIV, then a lower prevalence should be assumed for the reported study including HIV-negative women.
•Response: Yes, this is logically true. But to initiate our study, we base on a 4 years report from the regional health bureau and PATHFINDER, Ethiopia, Sep, 2014. In the report, 111 HIV unknown status were counseled and all were screened. Based on the result found, 29(26%) were VIA +ve for pre-cancerous cervical lesion with no specified reason. Therefore, we thought that it could be more and taking the 6.7% may provide less number of samples that could not represent the region and we decide to use if there is greater prevalence. We did not find any recent community based study in the region that could give us large sample size; for that reason we consider the prevalence that can provide us maximum sample size (50%). The drawback of this may be maximizing the cost of the study.
2. Previous comment: Please state which variables were included in the multivariable model finally.
Author reply: Dear/Sir/Madam: the model variables are mentioned under the “factors associated---,” sub section, in paragraph two.
Response: From that paragraph it is not clear which variables were included in the final multivariable model. Furthermore, the authors should explain how they chose variables for inclusion in the multivariable model. This information should be given in the methods section.
•Response: We apologize for our previous response. As now included under the analysis sub section, the variables with p-value < 0.05 in the unadjusted multinomial regression was considered as significant and used in the multivariable multinomial logistic regression.

3. Eligibility criteria: “all adult women” – please state an age range. Were women eligible for screening from the age of 15 already? What do the national cervical cancer screening guidelines recommend?
•Response: We understand and accept the concern. When we depend on the national guideline, it recommends 30 – 49 years old female to be eligible for screening. This manual is developed based on different developing countries. Meaning, in our set up, there are 9 years old married (starting of sexual contact). The range of 30-49 ages

implies that they started sex in the least 5 (immune-compromised) to 10 years (which is at 20 years to 44 years). In our set up, it is different, (which may be 14 - 19 years); for that we can find at least 15 to 19 years old married women stayed 5 to 10 yeast after sexual initiation on the ground. Moreover, this guideline is focusing on the HPV caused cervical cancer screening. But PHV is not the only factor that can cause cervical cancer. As part of our professional activities, we had faced with 16 years old women who had advanced cervical CA. This may be caused by other factors (not HPV) that are not related to early sexual initiation. Therefore, we consider 16 to 65 years old women who are with sexual experience (at least in the last 5 years) provided that they are living independent of their family or with husband for the justification of Ethical clearance. This provides a good coverage of eligible women in the community.

Results:
1. Previous comment: How was history of STI determined? Symptoms? Treated STI?
Author response: We determine the STI history based on items related to symptom of the
infections taken from the national syndrome approach of the STI guideline.
Comment: This information should be added to the manuscript.

•Response: We accept the comment and we used - having history of STI, if any one of the S/S is present (discharge, offensive secretion, itching, dysuria, lower abdominal pain, and fever). We also added it to the manuscript at the last statement of the data collection procedure sub section.

2. Previous comment: Why was history of cervical cancer screening not included as a potential predictor? It would have been interesting to see how many women had been screened previously, and whether that was associated with having cervical precancer.
Author response: we respect your valuable comment. First of all, there was no routine cervical screening mechanism in the country; except for HIV cases started 2011. In the absence of service, asking utilization is not logical.
Comment: This background information should be added to the manuscript as readers may not be aware of this.
•Response: We accept the concern, and we added it to the manuscript at the last statement of the data collection procedure sub section.

3. Table 4: For some of the variables (e.g., age) there is no reference category for aRRR. The analysis looks wrong.
•Response: We accept the concern, and 15-30 is the reference and we corrected it in the table (wrongly, last values of the category is pasted to first category in both positive and suspicious outcome variables).
4. Table 4: explanations for *, **, and *** are missing.
•Response: We accept your concern, it is included under the table as NB: P - value < 0.001 = ***, 0.001 - 0. 009 = ** and 0. 010 - 0. 05 = * and cRRR – crude relative risk ratio, aRRR - Adjusted relative risk ratio, C.I. - Confidence Interval

Discussion:

1. Previous comment: Page 12, line 232: HIV status mentioned here, but not in the results section?
Author response: We accept the comment and correction is made accordingly.
Comment: What is the correction? That HIV status is removed entirely? Does that mean that no information was available on HIV status?

•Response: At first, it was included as variable, and the statistician was used the raw data to analyze the draft result. But at that time when we evaluate the data collection process, we addressed that the collected data on the variable 'HIV' status was incomplete (some sites has no HIV testing clinic and filled as unknown, which could not be logically true to treat those sites with sites that have HIV clinic). Hence we agreed to discard this variable to prevent error. The problem that we included it at the first draft was because of all Co-authors did not meet in person, since they live at different countries (Addis Ababa, Mekelle - Tigray, German, and Chicago (USA).

2. Previous comment: The authors should discuss the limitations of their study.
Author response: We accept the comment and correction is made accordingly.

Powered by Editorial Manager® and ProduXion Manager® from Aries Systems Corporation

	Comment: Discussion of study limitations is insufficient.<br><br>•Response: We accept the concern, and we have tried to incorporate more limitations as much as we can. We believe limitations beyond the study may not be explanatory for our findings.<br><br>3. Previous comment: The discussion is mainly a comparison with results from other studies. It would be helpful if the authors could also expand on hypotheses and explanations of why certain factors may predict cervical pre-cancer. For example, why would divorced and widowed women be at increased risk of precancer after adjustment for age? Any hypotheses?<br>Author response: We accept the comment and correction is made accordingly.<br>Comment: This comment has been inadequately addressed, and the discussion section is difficult to understand sometimes. E.g., “In Ethiopia, long time contraceptive users are few, which is evidenced by having high fertility rate. This may not predispose to cervical pre-cancerious lesion.” – what does that mean? What does not predispose to precancerous cervical lesions? The discussion section needs to be restructured and improved.<br><br>•Response: We accept the comment and tried to go through again to incorporate some hypothesis. It can be seen from track changes file.<br>____<br>7. PLOS authors have the option to publish the peer review history of their article (what does this mean?). If published, this will include your full peer review and any attached files.<br><br>If you choose “no”, your identity will remain anonymous but your review may still be made public.<br><br>Do you want your identity to be public for this peer review? For information about this choice, including consent withdrawal, please see our Privacy Policy.<br>Reviewer #1: No<br>Reviewer #2: No<br>[NOTE: If reviewer comments were submitted as an attachment file, they will be attached to this email and accessible via the submission site. Please log into your account, locate the manuscript record, and check for the action link "View Attachments". If this link does not appear, there are no attachment files.]<br><br>While revising your submission, please upload your figure files to the Preflight Analysis and Conversion Engine (PACE) digital diagnostic tool, https://pacev2.apexcovantage.com/. PACE helps ensure that figures meet PLOS requirements. To use PACE, you must first register as a user. Registration is free. Then, login and navigate to the UPLOAD tab, where you will find detailed instructions on how to use the tool. If you encounter any issues or have any questions when using PACE, please email PLOS at figures@plos.org. Please note that Supporting Information files do not need this step.<br><br>____<br>In compliance with data protection regulations, you may request that we remove your personal registration details at any time. (Remove my information/details). Please contact the publication office if you have any questions.
**Additional Information:**	
**Question**	**Response**
**Financial Disclosure**<br><br>Enter a financial disclosure statement that describes the sources of funding for the	This project is funded by Mekelle University, Mekelle, Ethiopia and Tiray Regiona Health Bureau with the registration number of CRPO/CHS/Ext001/08/2015. “The author(s) received no any funding for this paperwork or manuscript publication from any source." “The Mekelle University and Regional Health Bureau had no role in study

work included in this submission. Review the submission guidelines for detailed requirements. View published research articles from *PLOS ONE* for specific examples.<br><br>This statement is required for submission and **will appear in the published article** if the submission is accepted. Please make sure it is accurate.<br><br>**Unfunded studies**<br>Enter: *The author(s) received no specific funding for this work.*<br><br>**Funded studies**<br>Enter a statement with the following details:<br>• Initials of the authors who received each award<br>• Grant numbers awarded to each author<br>• The full name of each funder<br>• URL of each funder website<br>• Did the sponsors or funders play any role in the study design, data collection and analysis, decision to publish, or preparation of the manuscript?<br>• **NO** - Include this sentence at the end of your statement: *The funders had no role in study design, data collection and analysis, decision to publish, or preparation of the manuscript.*<br>• **YES** - Specify the role(s) played.<br><br>* typeset	design, data collection and analysis, decision to publish, or preparation of the manuscript."
**Competing Interests**<br><br>Use the instructions below to enter a competing interest statement for this submission. On behalf of all authors, disclose any competing interests that could be perceived to bias this work—acknowledging all financial support and any other relevant financial or non-financial competing interests.<br><br>This statement **will appear in the published article** if the submission is accepted. Please make sure it is accurate. View published research articles from *PLOS ONE* for specific examples.	The authors have no competing interests to declare

Powered by Editorial Manager® and ProduXion Manager® from Aries Systems Corporation

**NO authors have competing interests**<br><br>Enter: *The authors have declared that no competing interests exist.*<br><br>**Authors with competing interests**<br><br>Enter competing interest details beginning with this statement:<br><br>*I have read the journal's policy and the authors of this manuscript have the following competing interests: [insert competing interests here]*<br><br>* typeset	
**Ethics Statement**<br><br>Enter an ethics statement for this submission. This statement is required if the study involved:<br><br>• Human participants<br>• Human specimens or tissue<br>• Vertebrate animals or cephalopods<br>• Vertebrate embryos or tissues<br>• Field research<br><br>Write "N/A" if the submission does not require an ethics statement.<br><br>General guidance is provided below. Consult the submission guidelines for detailed instructions. **Make sure that all information entered here is included in the Methods section of the manuscript.**	The study protocol was evaluated and approved by the Research Ethics Review Committee of College of Health Sciences, Mekelle University. Official cooperation letters were obtained from Mekelle University, Tigray Regional Health Bureau, and zonal health offices. Moreover, prior conducting the study, the purpose and objective of the study were described to the study participants and a written informed consent was obtained. The consent involves permission to disseminate the findings of the study through scientific workshop and publish in reputable journals. The study participants were told that they have full right to discontinue the study. Confidentiality and any special data security requirements were maintained and assured. The results of the screening that have a direct benefit to the health of the study participants were informed to physicians working in the nearby health facility.

Powered by Editorial Manager® and ProduXion Manager® from Aries Systems Corporation

**Format for specific study types**<br><br>**Human Subject Research (involving human participants and/or tissue)**<br>• Give the name of the institutional review board or ethics committee that approved the study<br>• Include the approval number and/or a statement indicating approval of this research<br>• Indicate the form of consent obtained (written/oral) or the reason that consent was not obtained (e.g. the data were analyzed anonymously)<br><br>**Animal Research (involving vertebrate animals, embryos or tissues)**<br>• Provide the name of the Institutional Animal Care and Use Committee (IACUC) or other relevant ethics board that reviewed the study protocol, and indicate whether they approved this research or granted a formal waiver of ethical approval<br>• Include an approval number if one was obtained<br>• If the study involved *non-human primates*, add *additional details* about animal welfare and steps taken to ameliorate suffering<br>• If anesthesia, euthanasia, or any kind of animal sacrifice is part of the study, include briefly which substances and/or methods were applied<br><br>**Field Research**<br><br>Include the following details if this study involves the collection of plant, animal, or other materials from a natural setting:<br>• Field permit number<br>• Name of the institution or relevant body that granted permission	
**Data Availability**<br><br>Authors are required to make all data underlying the findings described fully available, without restriction, and from the time of publication. PLOS allows rare exceptions to address legal and ethical concerns. See the PLOS Data Policy and FAQ for detailed information.	Yes - all data are fully available without restriction

A Data Availability Statement describing where the data can be found is required at submission. Your answers to this question constitute the Data Availability Statement and **will be published in the article**, if accepted.<br><br>**Important:** Stating 'data available on request from the author' is not sufficient. If your data are only available upon request, select 'No' for the first question and explain your exceptional situation in the text box.<br><br>Do the authors confirm that all data underlying the findings described in their manuscript are fully available without restriction?	
**Describe where the data may be found in full sentences. If you are copying our sample text, replace any instances of XXX with the appropriate details.**<br><br>• If the data are **held or will be held in a public repository**, include URLs, accession numbers or DOIs. If this information will only be available after acceptance, indicate this by ticking the box below. For example: *All XXX files are available from the XXX database (accession number(s) XXX, XXX.).*<br>• If the data are all contained **within the manuscript and/or Supporting Information files**, enter the following: *All relevant data are within the manuscript and its Supporting Information files.*<br>• If neither of these applies but you are able to provide **details of access elsewhere**, with or without limitations, please do so. For example:<br><br>*Data cannot be shared publicly because of [XXX]. Data are available from the XXX Institutional Data Access / Ethics Committee (contact via XXX) for researchers who meet the criteria for access to confidential data.*<br><br>*The data underlying the results presented in the study are available from (include the name of the third party*	All data that are used to analyze this manuscript are included in the submitted manuscript

and contact information or URL).<br>• This text is appropriate if the data are owned by a third party and authors do not have permission to share the data.<br><br>* typeset	
Additional data availability information:	

Powered by Editorial Manager® and ProduXion Manager® from Aries Systems Corporation

Manuscript

Click here to access/download;Manuscript;Manscript final_March _30 2021 - edited TRHB - 2 Clean.doc

Epidemiology of Cervical Precancerous Lesion and Risk Factors Among Adult Women In Tigray, Ethiopia

Authors:

Gerezgiher B. Abera[1*], Henock G. Yebyo[2], Haftamu Hailekiros[3], Selam Niguse[3], Yibrah Berhe[3], Goitom Gigar[4], Tsehaye Asmelash[5], Gelila Goba[6]

Affiliation:

[1]School of Nursing, College of Health Sciences, Mekelle University, Mekelle, Ethiopia;

[2]School of Public Health, College of Health Sciences, Mekelle University, Mekelle, Ethiopia;

[3] School of Medicine, College of Health Sciences, Mekelle University, Mekelle, Ethiopia;

[4] Tigray Regional Health Bureau, Mekelle, Ethiopia;

[5]College of Health Sciences, Aksum University, Axum", Ethiopia;

[6]Department of Obstetrics and Gynecology, University of Illinois at Chicago, Chicago, USA

*__Corresponding Author__: E-mail: gbamsc2002@gmail.com

ORCID ID https://orcid.org/0000-0003-4968-2885

Abstract

Background: Cervical cancer is a preventable disease if treated early, but remains the second leading cause of cancer-related mortality among women in low- and middle-income countries. Data on epidemiology and risk factors in these settings are scarce. This study aimed to assess the prevalence of precancerous lesions and risk factors in Tigray region, Ethiopia.

Methods: A community-based cross sectional study was used and 900 participants were recruited using multistage sampling and 883 were ressponeded correctly. Data were collected using an interviewer administered questionnaire and screening with visual inspection with ascetic acid. Multinomial logistic regression analysis was conducted to estimate predictors.

Results: Seventy-nine (8.95%) women were positive for pre-cancer lesion and 35 (3.96%) were suspicious for cervical cancer. Divorced or widowed women had 2.5 and 4.7 times more risk to be positive and suspicious respectively, compared to single women (rrr=2.5, 95% CI [1.13, 5.52]); (rrr=4.69, 95% CI [1.00, 21.84]). The risk of having a suspicious result was 68% lower for women with primary education compared to those with no formal education (rrr=0.32, 95% CI [1.00, 21.84]). History of sexually transmitted infection was associated with positive pre-cancer lesion (rrr=1.91, 95% CI [1.11, 3.27]) whereas, being farmer (rrr=4.83, 95% CI [1.44, 16.13]), merchant (rrr=4.85, 95% CI [1.52, 15.46]), bleeding between periods (rrr =3.26, 95% CI [1.32, 8.04]) and pelvic or back pain (rrr =2.79, 95% CI [1.18, 6.58]) were associated with suspicious for cancer.

Conclusion: About 8.9% and 4% of the women were positive and suspicious for pre-cancer cervical lesion, respectively. Marital status, education, sexually transmitted infection, bleeding, and pelvic pain were predictors of cervical precancerous lesions.

Key words: Epidemiology, Cervical, Pre-cancerous lesions.

Introduction

Cervical cancer (CC) is a public health problem worldwide [1], primarily in low and middle-income countries, though data are scarce [2, 3]. The disease is fatal, but preventable if treated early [4]. The World Health Organization (WHO) indicates that CC kills an estimated 275,000 women every year and 500,000 new cases reported worldwide [1]. CC is the second largest killer among cancer-related death of women in low-and-middle income countries (LMICs) [4]. Reports from different countries show that the incidence of CC is high in developing countries and sexually transmitted infections (STIs), hormonal influences, genetics and participant factors are risk factors for CC [5, 6]. In Sub-Saharan Africa, an estimated 57,000 cases of CC occurred in 2000, comprising 22.2% of all female cancers [7].

A study from India revealed that late-stages reporting of disease was common with a peak age of CC incidence from 55 to 59 years and early marriage, multiple sexual partners, multiple pregnancies, oral contraceptives, and lack of awareness were significant risk factors [8]. A study in Uganda showed that 99% of women had heard about CC and 63% believed that family planning was a cause of CC as well as 85% recognizing inter-menstrual bleeding as a symptom of CC [9]. Other studies have also revealed the burden and public health importance of CC in LMICs [10-13].

In Ethiopia, cervical cancer is the most common cause of cancer death among female cancers with an overall mortality of 70% [14]. A study in Ethiopia showed that the mean age at diagnosis with cervical cancer was 48 years [15]. In a community based cross-sectional survey conducted in Gondar, northwest Ethiopia (2010), only 31% of respondents were knowledgeable about risk factors, symptoms, treatment options, prevention and early detection measures for cervical cancer [16]. Studies from Addis Ababa, Adama and Jimma showed that women 40 - 49 years of

age, having a history of STIs, having two or more lifetime sexual partners and contraceptive use were risk factors of the cervical pre-cancerous lesion [17- 19]. Another institution based studies in Tigray revealed a prevalence of precancerous cervical lesion of 6.7% and that STIs are predictors of the precancerous cervical lesions [20, 21].

The Ethiopian Ministry of Health officially launched guideline for cervical cancer prevention and control in 2005, describes a “See and Treat approach” using visual inspection with acetic acid (VIA) screening and cryotherapy treatment for the reason of feasibility and affordability (22).

Although Ethiopia’s federal ministry of health (FMoH) has given priority for CC prevention and control, there is a scarcity of data related to precancerous cervical lesions. Existing guidelines call for community based precancerous cervical lesions epidemiology and risk factors [22]. Accordingly, this community-based research determines the prevalence and factors associated with precancerous cervical lesions among adult women in Tigray, Ethiopia.

Method

Study setting and design

We conducted a community-based, cross-sectional study in Tigray, Northern Ethiopia. Data were collected from March 2016 to June 2017. The study was conducted in Tigray region, which has seven zones, 34 rural and 18 urban districts ('Woredas' in local name) and 763 sub-districts ('Kebele/Tabia' in local name). In 2017, the Tigray Region had one specialized hospital, 14 general hospitals, 22 primary hospitals, 214 health centers, and 712 health posts as well as more than 500 private health facilities.

Study Population

Study participants: All adult women living in Tigray region for about six months or more were considered as a source population, and those who were voluntary and selected as study subjects were the study population.

Eligibility criteria: All adult women, who were sexually active and voluntarily consented to participate were included in the study. Those with history of hysterectomy were excluded from the study.

Sample size and sampling technique:

The sample size was calculated with the aim of estimating a precise population prevalence of precancerous cervical lesions in the Tigray Region. As there is no baseline study of prevalence of precancerious cervical lesion, we assumed a 0.5 probability of the disease, 0.05 probability of alpha testing, 80% power and design effect of 2 accounting for two-stage sampling. We added 10% to the calculated sample size to account for non-responses, yielding a sample size of 846, which we rounded to 900 for convenience.

A multistage sampling was used to select the study subjects. Considering 40% of the 52 districts in the region, 21 of the districts which were distributed proportionally to zones were selected randomly. Since there are 315 sub-districts in the selected districts, 20% of the sub-districts (a total of 63 sub-districts) were selected randomly. Study participants were allocated proportionally to sub-districts based on the number of participants fulfilling the eligibility criteria in each sites. This was taken from the health extension workers registration book.

Recruitment process: A large mobilization was preceded the screening. This effort entailed training of Health Extension Workers (HEWs) from selected sub-districts. HEWs in turn oriented women in their communities. They also mobilized Women Development Army (WDA) leaders and members as well as sub-district leaders by providing brief orientations about cervical cancer (CC) as well as upcoming screening dates and locations. Grassroots mobilization was complimented by promotion on regional radio stations by the Tigray Regional Health Bureau (TRHB).

Following community mobilization, participants were invited for screening to a total of 17 primary and general hospitals in the sub districts which have cervical cancer screening cencers using visual inspection with acetic acid (VIA). As per the estimated number of expected participants, a key value of 4 was calculated. Every forth wo[illegible] arriving at the hospitals following grassroots mobilization were selected and invited to participate in the study. Finally, those who fulfill the eligibility criteria were recruited as a study subject.

Data collection procedure:

Women providing consent were interviewed using an interviewer administered questionnaire to obtain socio-demographic and risk-factors information, including health related behaviors and lifestyle, knowledge, attitude, and clinical attributes.

Clinical testing was conducted by certified nurses trained by the TRHB according to the national cervical cancer prevention guidelines [23]. A Visual Inspection with Acetic Acid (VIA) screening protocol was developed based on the national guideline, The protocol included private exam area, examination table, trained health professionals, adequate light source, sterile vaginal speculum, new examination or surgical gloves, large cotton swabs, diluted (3-5%) acetic acid, small bowl, containers with 0.5% chlorine solution, plastic bucket with a plastic bag and quality assurance system to maximize accuracy.

Precancerous cervical lesions w[illegible] determined by vaginal speculum exam during which provider applied diluted acetic acid to the cervix. Visual confirmation of color change by the naked eye was used to determine the presence or absence of lesions. The test was considered positive if a sharp, distinct, well-defined, dense (opaque/dull or oyster) Aceto-white area was present with raised margins touching the Squamo-columnar junction (SCJ). The test was considered negative if no or faint Aceto-white lesions and/or smooth surface was present. The test was ci[illegible]dered suspicious for CC where clinically visible ulcerative, cauliflower-like growth or ulcer, oozing, and/or bleeding on touch were observed.

Data analysis

Data was analyzed using STATA version14. Data entry and clearance was done using descriptive analysis to check if any missing and inappropriate categories. Descriptive statistics were presented in texts and tables. The dependent variable was cervical pre-cancer status, in which the screening result had three possible outcomes: Negative, Positive and Suspicious for VIA. A multinomial regression model was used to check the influence of socio-demographic, risk factors and reproductive health characteristics on CC screening outcomes. Unadjusted multinomial regression was used to examine the separate bivariate relationship between each

independent variables and the dependent variable. Relative risk ratios (rrr) were used to assess the multivariate association of the predictor variables with the dependent variable.

Ethical consideration: Before beginning data collection ethicalclearance was obtained from mekelle university, collage of health sience research and community service commiyyee and support letter was obtained from Tigray regional health bureau. Participants were oriented the general objective of the study, procedure of data collection, privacy and confidenciality of the information obtained, it was also informed that participation is voluntary based and the possibility to withdraw at any time when they need. Finally written informed consent was obtained from the participants prior to the actual data collection.

Results

Characteristics of the study participants

A total of 883 questionnaires [illegible] collected with complete information, equating to a response rate of 98.1%. More than half of the participants 496 (56.2%) came from urban districts. Mean age of participants was 35.69 years (SD ±8.45, range 16 - 62). Among all respondents, 532 (60.2%) were married, 723 (81.9%) were Orthodox Christian, 286 (32.4%) had no formal education, 282 (31.9%) had no formal work, and 717 (81.2%) had zero to four children. Median income was Ethiopian Birr 1,500 and 515 (58.3%) women earned between Ethiopian Birr 0 and 2,000 per month [Table 1].

Table 1. Distribution of Socio-demographic characteristics by VIA results among women of reproductive age in Tigray, (n=883)

Variables	Groups	VIA results			Total
		**Negative** N (%)	**Positive** N (%)	**Suspicious** N (%)	**Total** N (%)
Residence	Rural	345(89.1)	28(7.2)	14(3.6)	387(43.8)
	Urban	424(85.5)	51(10.3)	21(4.2)	496(56.2)
Age (yr)	15-30	257(86.5)	30(10.1)	10(3.4)	297 (33.6)
	31-45	416(87.6)	44(9.3)	15(3.2)	475 (53.8)
	>=46	96(86.5)	5(4.5)	10(9.0)	111 (12.6)
Marital status	Single	208(92.0)	14(6.2)	4(1.8)	226(25.6)
	Married	472(88.7)	42(7.9)	18(3.4)	532(60.2)
	Divorced/Others	89(71.2)	23(18.4)	13(10.4)	125(14.2)
Religion	Orthodox	627(86.7)	64(8.9)	32(4.4)	723(81.9)
	Muslim and others	142(88.8)	15(9.4)	3(1.9)	160(18.1)
Educational status	No formal education	240(83.9)	25(8.7)	21(7.3)	286(32.4)
	Primary education	325(89.0)	30(8.2)	10(2.7)	365(41.3)
	Secondary education	116(85.3)	17(12.5)	3(2.2)	136(15.4)
	Tertiary education	88(91.7)	7(7.3)	1(1.0)	96(10.9)
Occupation	Civil servant	209(93.3)	11(4.9)	4(1.8)	224(25.4)
	Farmer	141(81.5)	21(12.1)	11(6.4)	173(19.6)
	Merchant	172(84.3)	18(8.8)	14(6.9)	204(23.1)
	No formal work	247(87.6)	29(10.3)	6(2.1)	282(31.9)
Monthly income	0-2000	439(85.2)	51(9.9)	25(4.9)	515(58.3)
	2001-4000	158(86.3)	16(8.7)	9(4.9)	183(20.7)
	4001-6000	105(92.1)	8(7.0)	1(0.9)	114(12.9)
	6001-8000	16(88.9)	2(11.1)	0(0.0)	18(2.0)
	8001-10000	51(96.2)	2(3.8)	0(0.0)	53(6.0)
Parity	0-4 children	628(87.6)	67(9.3)	22(3.1)	717(81.2)
	5-12 children	141(84.9)	12(7.2)	13(7.8)	166(18.79)

Cervical precancerous lesion:

A total of 782 (88.6%) had visible SCJ. Among all the participants, 79 (8.9%) were positive for VIA, 35 (4.0%) were suspicious for cancer, and 769 (87.1%) were negative for pre-cancer lesions. A total of 776 (87.9%) reported that their age at first sexual contact was less than 20 years. Of the total, 344 (39.0%) respondents had more than two sexual partners in their lifetime and 199 (22.5%) experienced STIs. Thirty-two (3.6%) were smokers and one-third of respondents consumed alcohol 255 (28.9%). Corticosteroids were used in 97 (11.0%) women and 199 (22.5%) where current contraceptive users. Seventy (7.9%) had a family history of CC. Out of these women, nine (12.9%) were VIA-positive [Table 2].

Table. 2. Distribution of risk factors by VIA results among women of reproductive age in Tigray, (n=883).

**Risk factors of cervical cancer**		**VIA results**			**Total** N (%)
		**Negative** N (%)	**Positive** N (%)	**Suspicious** N (%)	
Age at first sexual contact	≤ 20 years age	675(87.0)	66(8.5)	35(4.5)	776(87.9)
	> 20 years age	94(87.9)	13(12.1)	0(0.0)	107(12.1)
Number Sexual Partner	Only one	461(88.3)	45(8.6)	16(3.1)	522(59.1)
	Two or more	293(85.2)	33(9.6)	18(5.2)	344(39.0)
	No or unspecified	15(88.2)	1(5.9)	1(5.9)	17(1.9)
History of STI	Yes	156(78.4)	32(16.1)	11(5.5)	199(22.5)
	No	613(89.6)	47(6.9)	24(3.5)	684(77.5)
Active smoking	Yes	29(90.6)	2(6.2)	1(3.1)	32(3.6)
	No	740(87.0)	77(9.0)	34(4.0)	851(96.4)
Alcohol consumption	Yes	232(91.0)	18(7.1)	5(2.0)	255(28.9)
	No	537(85.5)	61(9.7)	30(4.8)	628(71.1)
Chronic corticosteroids use	Yes	86(88.7)	8(8.2)	3(3.1)	97(11.0)
	No	683(86.9)	71(9.0)	32(4.1)	786(89.0)
Current contraceptive use	Yes	169(84.9)	21(10.6)	9(4.5)	199(22.5)
	No	600(87.7)	58(8.5)	26(3.8)	684(77.5)
Family history of cervical cancer	Yes	60(85.7)	9(12.9)	1(1.4)	70(7.9)
	No	709(87.2)	70(8.6)	34(4.2)	813(92.1)

From the study participants, 183 (20.7%) women experienced bleeding between menstruation, 94 (10.6%) experienced heavy menstruation, 176 (19.9%) experienced contact bleeding and 152

(17.2%) women experienced pain during sex. Pelvic or back pain was reported by 267 (30.2%) women [Table 3].

Table 3. Distribution of signs and symptoms by VIA results among women of reproductive age in Tigray, (n=883).

**Sign and symptoms and cervical cancer status**		**VIA results**			
		**Negative** N (%)	**Positive** N (%)	**Suspicious** N (%)	**N (%)**
Bleeding between menstruation	Yes	149(81.4)	16(8.7)	18(9.8)	183(20.7)
	No	620(88.6)	63(9.0)	17(2.4)	700(79.3)
Heavy menstruation	Yes	70(74.5)	12(12.8)	12(12.8)	94(10.6)
	No	699(88.6)	67(8.5)	23(2.9)	789(89.4)
Bleeding after menopause	Yes	78(77.2)	14(13.9)	9(8.9)	101(11.4)
	No	691(88.4)	65(8.3)	26(3.3)	782(88.6)
Contact bleeding	Yes	143(81.2)	19(10.8)	14(8.0)	176(19.9)
	No	626(88.5)	60(8.5)	21(3.0)	707(80.1)
Pain during sex	Yes	120(78.9)	23(15.1)	9(5.9)	152(17.2)
	No	649(88.8)	56(7.7)	26(3.6)	731(82.8)
Pelvic or back pain	Yes	213(79.8)	33(12.4)	21(7.9)	267(30.2)
	No	556(90.3)	46(7.5)	14(2.3)	616(69.8)
Unusual vaginal discharge	Yes	239(79.9)	37(12.4)	23(7.7)	299(33.9)
	No	530(90.8)	42(7.2)	12(2.1)	584(66.1)

Factors associated with cervical screening VIA resul[illegible]

In the multinomial analysis “Negtive result of the dependent variable was used as a base reference against the positive and suspicious results; hence the relative risk is relative to those who have negative results.

Multinomial bivariate analysis revealed that age, marital and education status, occupation, symptoms of CC, parity, STI history of partner, and lower abdominal pain were significantly associated with positive or suspicious results (p-value < 0.05). Other variables were not significantly associated with cervical precancerous lesions. The multivariate logistic regression analysis showed that marital and educational status, occupation, history of STIs, bleeding between menstruation, and pelvic or back pain were significantly associated with the presence of cervical precancerous lesions. Divorced or widowed women had 2.5 and 4.7 times risk, to be

VIA-positive and suspicious respectively compared to single women (rrr=2.50, 95% CI [1.13, 5.52]); (rrr=4.69, 95% CI [1.00, 21.84]). The risk of being suspicious was 68% lower for women with primary education compared to those with no formal education (rrr=0.32, 95% CI [1.00, 21.84]). The risk of being suspicious for cancer was 4.8 times higher among farmers (rrr=4.83, 95% CI [1.44, 16.13]) and merchants (rrr=4.85, 95% CI [1.52, 15.46]) compared to respondents with no formal work.

The risk of being VIA-positive was 90% higher among women with a history of STIs (rrr=1.91, 95% CI [1.11, 3.27]). The risk of being suspicious for cancer was 3.26 times higher among women who experienced bleeding between periods (rrr =3.26, 95% CI [1.32, 8.04]). Women with a history of pelvic or back pain were 2.7 times more likely to be suspicious for cancer (rrr =2.79, 95% CI [1.18, 6.58]) [Table 4].

Table.4. Bivariate and Multivariable analysis of independent variables with cervical cancer status among reproductive age women in Tigray, (n=883).

Variables			VIA results					
		Negative	Positive			Susp[illegible]s		
		N(%)	*N(%)*	*cRRR [95% CI]*	*aRRR [95% C.I.)*	*N(%)*	*CRRR [95% CI]*	*ARRR [95% C.I.)*
Age	15-30	257(86.5)	30(10.1)	1	0.91(0.53, 1.55)	14(3.6)	1	0.58(0.22, 1.55)
	31-45	416(87.6)	44(9.3)	0.91 (0.56, 1.48)	0.50(0.17, 1.45)	21(4.2)	0.93(0.410, 2.09)	2.34(0.74, 7.36)
	>=46	96(86.5)	5(4.5)	0.45(0.17, 1.18)	0.91(0.53, 1.55)	10(3.4)	2.68(1.08, 6.63) *	0.58(0.22, 1.55)
Marital status	Single	208(92.0)	14(6.2)	1	1	4(1.8)	1	1
	Married	472(88.7)	42(7.9)	1.32(0.71, 2.47)	0.94(0.46, 1.91)	18(3.4)	1.98(0.66, 5.93)	2.15(0.51, 8.97)
	Divorced/widowed	89(71.2)	23(18.4	3.84(1.89, 7.80)***	2.50(1.13, 5.52)*	13(10.4	7.59(2.41, 23.93)***	4.69(1.00, 21.84)*
Education al status	No formal education	240(83.9)	25(8.7)	1	1	21(7.3)	1	1
	Primary Education	325(89.0)	30(8.2)	0.89(0.51, 1.55)	0.78(0.41, 1.48)	10(2.7)	0.35(0.17, 0 .76) ***	0.32(0.11, 0.91)*
	Secondary Education	116(85.3)	17(12.5)	1.41(0.73, 2.71)	1.15(0.54, 2.43)	3(2.2)	0.29(0.09, 1.01)	0.25(0.05, 1.09)
	Tertiary Education	88(91.7)	7(7.3)	0.76(0.32, 1.83)	0.84(0.29, 2.39)	1(1.0)	0.13(0.02, 0.98)*	0.12(0.01, 1.41)
Occupatio n	Civil Servant	209(93.3)	11(4.9)	0.45(0.22, 0.92)*	0.78(0.41, 1.48)	4(1.8)	0.78(0.22, 2.82)	2.67(0.48, 14.68)
	Farmer	141(81.5)	21(12.1)	1.26(0.69, 2.30)	1.15(0.54, 2.43)	11(6.4)	3.21(1.16, 8.87)*	4.83(1.44, 16.13)*
	Merchant	172(84.3)	18(8.8)	0.89(0.47, 1.65)	0.84(0.29, 2.39)	14(6.9)	3.35(1.26, 8.89)*	4.85(1.52, 15.46)**
	No formal work	247(87.6)	29(10.3)	1	1	6(2.1)	1	1
Parity	0-4 children	628(87.6)	67(9.3)	1	1	22(3.1)	1	
	5-12 children	141(84.9)	12(7.2)	0.79(0.42, 1.51)	0.85(0.40, 1.79)	13(7.8)	2.63(1.29, 5.35)**	1.62(0.61, 4.25)
History of STI	Yes	156(78.4)	32(16.1)	2.67(1.65, 4.33)***	1.91(1.11, 3.27)*	11(5.5)	1.80(0.86, 3.75)	0.99(0.40, 2.42)
	No	613(89.6)	47(6.9)	1	1	24(3.5)	1	1
Blood between period	Yes	149(81.4)	16(8.7)	1.05(0.59, 1.88)	0.71(0.35, 1.45)	18(9.8)	4.40(2.21, 8.75)***	3.26(1.32, 8.04)*
	No	620(88.6)	63(9.0)	1	1	17(2.4)	1	1
Heavy menstruati[illegible]	Yes	70(74.5)	12(12.8)	1.78(0.92, 3.46)	1.23(0.55, 2.74)	12(12.8)	5.20(2.48, 10.9)***	2.45(0.92, 6.52)
	No	699(88.6)	67(8.5)	1	1	23(2.9)	1	1
Bleeding after [illegible]	Yes	78(77.2)	14(13.9)	1.90 (1.02, 3.55)*	1.25(0.57, 2.71)	9(8.9)	3.06 (1.38, 6.77) **	1.27(0.40, 3.99)
	No	691(88.4)	65(8.3)	1	1	26(3.3)	1	1
Contact bleeding	Yes	143(81.2)	19(10.8)	1.38(0.80, 2.39)	0.85(.42, 1.69)	14(8.0)	2.91(1.44, 5.87)**	0.81(0.30, 2.19)
	No	626(88.5)	60(8.5)	1	1	21(3.0)	1	1
Pain during sex	Yes	120(78.9)	23(15.1)	2.22(1.31, 3.74)**	1.39(0.70, 2.74)	9(5.9)	1.87(0.85, 4.09)	0.69(0.23, 2.01)
	No	649(88.8)	56(7.7)	1	1	26(3.6)	1	1
Pelvic or back pain	Yes	213(79.8)	33(12.4)	1.87(1.16, 3.00)*	1.60(0.90, 2.85)	21(7.9)	3.91(1.95, 7.84)***	2.79(1.18, 6.58)*
	No	556(90.3)	46(7.5)	1	1	14(2.3)	1	1
Unusual vagina [illegible]	Yes	239(79.9)	37(12.4)	1.95(1.22, 3.11)**	1.39(0.78, 2.49)	23(7.7)	4.26 (2.08, 8.68)***	2.22(0.91, 5.38)
	No	530(90.8)	42(7.2)	1	1	12(2.1)	1	1

Discussion

Ethiopia's Federal Ministry of Health has prioritized CC prevention and treatment [23]; however, there is a scarcity of data related to the prevalence of precancerous cervical lesions and abnormal screening results. This study explored the prevalence of precancerous lesions and abnormal results in Tigray, Northern Ethiopia.

Our study showed that 79 (8.9%) respondents were VIA-positive and 35 (4.0%) were suspicious for CC. The overall prevalence of VIA-positive was higher in this study than a study at Alameda Textile Factory in Tigray, which showed a VIA-positive rate of 6.7% [20]. A study at a Family Guidance Association of Ethiopia (FGAE) Clinic in Jimma, southern Ethiopia, showed that 12.9% of women were VIA-positive [19]. Studies in Swaziland, Cameroon, and Jakarta, Indonesia, showed VIA-positive rates of 15%, 3.33%, and 4.7%, respectively [10, 11, 12]. Our study also showed that 4.0% of women were suspicious for cancer compared to 1.2% of women in the study done in Jimma [19]. Though most are similar, the difference might be because of the skill difference of the care providers, and type acetic acid used for screening.

After adjusting for other variables, marital and educational status, occupation, history of STIs, bleeding between period, and pelvic or back pain were statistically significant risk factors for VIA-positive or suspicious for cancer. Divorced or widowed women had a risk of being VIA-positive (p=0.023) and being suspicious (p=0.049) for CC than single women. In a study in Dar Es Salaam, Tanzania, divorced/separated women were more likely to be VIA-positive [13]. In Jakarta, Indonesia, 8.3% of VIA-positive women were married more than once compared to 4.7% of overall women [12]. Women who have started sexual practice and have no permanent husband had a chance of exposing to multiple sexual partner; which increases the risk of exposure to HPV. Increasing outreach and screening intensity to divorced, separated, and

widowed women could be a simple approach to streamlining the identification and treatment of women at risk for developing CC.

Women in our study with primary education were approximately 70% less likely to be suspicious for CC (p=0.034) than women with no education, though education was not significantly correlated with VIA-positive. What's more, higher education levels, including secondary and tertiary, were not significantly associated with VIA-positive or suspicious. In Dar Es Salaam, women with low education had odds 4.3 higher of being VIA-positive [13]. Studies in Gondar, Ethiopia, and India showed that lack of awareness of CC was a risk factor for CC [7,16]. Increasing awareness including educational level could influence individuals to prevent themselves from any risk factors of cancer.

Women who are farmers and merchants had odds 4.8 times higher of being suspicious for CC in our study. Farmers may have less awareness of CC and lack the ability to obtain early screening. In the case of merchants, these women are wealthier than farmers in the study area and may have heightened risk due to more sexual practices that expose them to HPV.

Respondents with a history of STI had 1.91 times the risk of being VIA-positive (p=0.018), though STIs were not statistically significantly associated with suspicious results. This was similar to studies done in Addis Ababa, Adama and at the Alamed Textile Factory where the history of STI was a risk factor for cervical precancerous lesions [17, 18, 20]. One explanation for this is that women with STIs are more prone to HPV infection, which is by far the dominant pathway for CC to initiate and progress.

Bleeding between period and pelvic or back pain were significantly associated with suspicious results, which were 3.26 (p=0.010) and 2.79 (p=0.019) times higher risk respectivelythan their counter parts. A study from Uganda revealed that the participants recognized inter-menstrual

bleeding as a symptom of CC [9]. This is mainly related to suspicious for cancer that as the lesion advanced, the common sign and symptom are vaginal bleeding and pain.

Among women 46 years of age or older, 9.0% were found to be suspicious for CC compared to approximately 9.5% for women less than 46 years of age. Though age was not statistically significant, further inquiry is needed to confirm and explain these variations. In a study in India, women 55 to 59 years of age had the highest risk of CC [8], this was also observed in one Ethiopian study [15]. Ethiopian women may have earlier sexual initiation than Indian women that delaying introduction and progression of the disease to pre-cancer lesion.

In the present study, the presence of cervical precancerous lesion was not associated with current contraceptive use or age at first sexual contact. The age at first sexual contact was significant predictor for cervical precancerous lesion in the study done in Jimma [19]. A study in Jakarta showed that current use of hormonal contraceptives was associated with odds of VIA-positive [12], whereas studies in Addis Ababa and Adama (Ethiopia), Uganda, and India showed that contraceptive use was a risk-factor for precancerous cervical lesions [9,8,17,18]. In Ethiopia, long time contraceptive users are few, which is evidenced by having high fertility rate. This may not predispose to cervical precancerious lesion.

Strenngth and limitation of the study:

The primary data of cervical precancerous lesion was acertaned using VIA screening, rather than history taking. This method of data collection minimizes recall bias. However a case control study design could better estimate the association of independent variables with the outcome variable.

Conclusion

In this study, 8.9% and 4% of the women were positive and suspicious for pre-cancer cervical lesion, respectively. Marital status, educational status, occupation, history of an STI, bleeding between period, and pelvic or back pain were risk factors for pre-cancer cervical lesion. Cervical cancer prevention is better to be strengthened focusing on preventable risk factors, including exposure to sexually transmitted infections and specific vulnerable groups like divorced and widowed women. Further study of the age distribution of precancerous lesions and suspicious results is merited.

List of Abbreviations

ARRR – Adjusted Relative Risk Ratio

cRRR - Crude Relative Risk Ratio

HIC - High Income Countries

HIV - Human Immunodeficiency Virus

HPV - Human Papilloma Virus

LMIC - Low Income Countries

RRR - Relative Risk Ration

STI - Sexually Transmitted Infection

SD - Standard Deviation

SCJ - Squamous-Columnar Junction

VIA - Visual Inspection With Acetic acid

Acknowledgments:

Our great appreciation goes to healthcare workers of the health facilities involved in the data collection and all study participants of the study. Moreover we would like to extend our acknowledgment for administrative and community health care workers for their support during data collection and facilitation of the project.

References

1. A report from WHO. A demonstration project, Prevention of cervical cancer through screening using visual inspection with acetic acid (VIA) and treatment with cryotherapy, African population and health research center, 2012.
2. American cancer society report, a guideline for cervical cancer and its managements, www.cancer.org Last Medical Review: 4/11/2013 and Last Revised: 1/31/2014
3. The Alliance for Cervical Cancer Prevention (ACCP), inspection with acetic acid (VIA): Evidence to date, www.alliance-cxca.org
4. Data derived from official reports by WHO, United Nations, Cervical Cancer Global Crisis Card, The World Bank, IARC Globocan http://www.who.int/hpvcentre/statistics/en/
5. Campbell C. Assessment of Cervical Cancer Incidence, Histopathology, and Screening Practices Among Hispanic Women in Latin America and Michigan, a dissertation for the degree of Doctor of Philosophy, 2011.
6. Zohre Momenimovahed and Hamid Salehiniya, Incidence, mortality and risk factors of cervical cancer in the world, Biomedical Research and Therapy, BioMedPress, 2018, http://www.bmrat.org/index.php/BMRAT/article/view/386
7. Rose I Anorlu, Cervical cancer: the sub-Saharan African perspective, Reproductive Health Matters, 2008;16(32)
8. Aswathy Sreedevi, Reshma Javed, and Avani Dinesh, Epidemiology of cervical cancer with special focus on India, Int J Womens Health. 2015; 7: 405–414, doi: 10.2147/IJWH.S50001

9. Amos D. Mwaka, Christopher G. Orach, Edward M. Were, Georgios Lyratzopoulos, Henry Wabinga, Martin Roland, Awareness of cervical cancer risk factors and symptoms: cross-sectional community survey in post-conflict northern Uganda, Health Expect. 2016 Aug; 19(4): 854–867, doi: 10.1111/hex.12382
10. Ginindza TG, Almonte M, Dlamini X, Sartorius B. Distribution of cervical abnormalities detected by visual inspection with acetic acid in Swaziland, 2011-2014: A retrospective study. *African J Prim Heal care Fam Med*. 2018;10(1):e1-e7. doi:10.4102/phcfm.v10i1.1773
11. Nkfusai NC, Mubah TM, Yankam BM, Tambe TA, Cumber SN. Prevalence of precancerous cervical lesions in women attending Mezam Polyclinic Bamenda, Cameroon. *Pan Afr Med J*. 2019;32:174. doi:10.11604/pamj.2019.32.174.16895
12. Anggraeni TD, Peters AAW. Prevalence , Age Distribution , and Risk Factors of Visual Inspection With Acetic Acid-Positive From 2007 to 2011 in Jakarta. 2017;22(2):103-107.
13. Kahesa C, Kjaer SK, Ngoma T, et al. Risk factors for VIA positivity and determinants of screening attendances in Dar es Salaam , Tanzania. *BMC Public Health*. 2012;12(1):1. doi:10.1186/1471-2458-12-1055
14. Haile T. Beyene M. Berg A. prevention of cervical cancer in Ethiopia, Senamoksh a report from Women´s Health Center, Addis Ababa, Ethiopia and ESOG, Ethiopia, a model clinic in Addis Ababa, 2018.
15. Dye D *et al*., 2009) Dye D., Solomon B., Claire H., Yared T., Vanessa H., Teshome D., Marion B., Anne R., **(2009**) Complex care systems in developing countries. *Breast Cancer Patient Navigation in Ethiopia* 116:577–85.)

16. Birhanu Z. et al, Health seeking behavior for cervical cancer in Ethiopia: a qualitative study, International Journal for Equity in Health, BioMed center, 2012, 11:83
17. Roza Teshome Kassa, Risk factors associated with precancerous cervical lesion among women screened at Marie Stops Ethiopia, Adama town, Ethiopia: a case control study, BMC Res Notes (2018) 11:145, https://doi.org/10.1186/s13104-018-3244-6.
18. Teame H, Addissie A, Ayele W, Hirpa S, Gebremariam A, Gebreheat G, et al. (2018) Factors associated with cervical precancerous lesions among women screened for cervical cancer in Addis Ababa, Ethiopia: A case control study. PLoS ONE 13(1): e0191506. https://doi.org/10.1371/journal.pone.0191506
19. Deksissa ZM, Tesfamichael FA, Ferede HA. Prevalence and factors associated with VIA positive result among clients screened at Family Guidance Association of Ethiopia, south west area office, Jimma model clinic, Jimma, Ethiopia 2013: A cross-sectional study Womens Health. *BMC Res Notes*. 2015;8(1):8-13. doi:10.1186/s13104-015-1594-x
20. Kebede Haile Misgina, Hailay Seyoum Belay and Teklehaymanot Huluf Abraha, Prevalence of precancerous cervical lesion and associated factors among women in North Ethiopia, Journal of Public Health and Epidemiology,2017, Vol. 9(3), pp. 46-50, DOI: 10.5897/JPHE2016.0891
21. Bayu H, Berhe Y, Mulat A, Alemu A (2016) Cervical Cancer Screening Service Uptake and Associated Factors among Age Eligible Women in Mekelle Zone, Northern Ethiopia, 2015: A Community Based Study Using Health Belief Model. PLoS ONE 11(3): e0149908. doi:10.1371/journal.pone.0149908
22. Yeneabeba Tilahun, National Cervical Cancer Prevention Training Package Participant Manual, Federal Ministry of Health Ethiopia, 2015.

23. Federal Democratic Republic of Ethiopia Ministry of Health. *Guideline for Cervical Cancer Prevention and Control in Ethiopia*.; 2015. https://www.iccp-portal.org/system/files/plans/Guideline Eth Final.pdf.

Manuscript
Click here to access/download;Manuscript;Manuscript- Feb - 12-2022.doc

Epidemiology of Pre-cancerous Cervical Lesion and Risk Factors among Adult Women n Tigray, Ethiopia

Authors:

Gerezgiher B. Abera[1*], Henock G. Yebyo[2], Haftamu Hailekiros[3], Selam Niguse[3], Yibrah Berhe[3], Goitom Gigar[4], Tsehaye Asmelash[5], Gelila Goba[6]

Affiliation:

[1]School of Nursing, College of Health Sciences, Mekelle University, Mekelle, Ethiopia;

[2]School of Public Health, College of Health Sciences, Mekelle University, Mekelle, Ethiopia;

[3] School of Medicine, College of Health Sciences, Mekelle University, Mekelle, Ethiopia;

[4] Tigray Regional Health Bureau, Mekelle, Ethiopia;

[5]College of Health Sciences, Aksum University, Axum", Ethiopia;

[6]Department of Obstetrics and Gynecology, University of Illinois at Chicago, Chicago, USA

***Corresponding Author**: E-mail: gbamsc2002@gmail.com

ORCID ID https://orcid.org/0000-0003-4968-2885

Abstract

Background: Cervical cancer is a preventable disease if treated early, but remains the second leading cause of cancer-related mortality among women in low and middle-income countries. Data on epidemiology and risk factors in these settings are scarce. This study aimed to assess the prevalence of precancerous lesions and risk factors in Tigray region, Ethiopia.

Methods: This was a community-based, cross-sectional study conducted among 900 women in Tigray region, Ethiopia, from March 2016 to June 2017.Data were collected using an interviewer administered questionnaire and screening with visual inspection with ascetic acid. Multinomial logistic regression analysis was conducted to estimate risk factors.

Results: Of the participants, 883 were responded correctly. Seventy-nine (8.95%) women were positive for pre-cancerous cervical lesion'and 35 (3.96%) were suspicious for cervical cancer. In the multivariable analysis, divorced or widowed women had higher risk of being positive for pre-cancerous cervical lesion'and or suspicious for cancer than single women. The risk of having a suspicious for cancer result was lower for women with primary education than those with no formal education. Participants who had history of sexually transmitted infection were at higher of being positive for pre-cancerous cervical lesion result . Participants who were farmer and merchant in occupation, those who had bleeding between periods and had pelvic or back pain were at risk of having suspicious for cervical cancer results.

Conclusion: About 8.9% and 4% of the women were positive for pre-cancerous cervical lesion'and suspicious for cancer, respectively. Marital status, education, sexually transmitted infection, bleeding, and pelvic pain were risk factors of pre-cancerous cervical lesion'.

Key words: Epidemiology, Cervical, Pre-cancerous lesions.

Introduction

Cervical cancer is a public health problem worldwide [1], primarily in low and middle-income countries, though data are scarce [2, 3]. The disease is fatal, but preventable if treated early [4]. The World Health Organization (WHO) indicates that cervical cancer kills an estimated 275,000 women every year and 500,000 new cases reported worldwide [1]. Cervical cancer is the second largest killer among cancer-related death of women in low-and-middle income countries (LMICs) [4]. Reports from different countries showed that the incidence of cervical cancer is high in developing countries and sexually transmitted infections (STIs), hormonal influences, genetics and participant factors (risk taking behavior, substance abuse, alcohol use) are risk factors for cervical cancer [5, 6]. In Sub-Saharan Africa, an estimated 57,000 cases of cervical cancer occurred in 2000, comprising 22.2% of all female cancers [7].

A study from India revealed that late-stages reporting of disease was common with a peak age of cervical cancer incidence from 55 to 59 years and early marriage, multiple sexual partners, multiple pregnancies, oral contraceptives, and lack of awareness were significant risk factors [8]. A study in Uganda showed that 99% of women had heard about cervical cancer and 63% believed that family planning was a cause of cervical cancer as well as 85% recognizing inter-menstrual bleeding as a symptom of cervical cancer [9]. Other studies have also revealed the burden and public health importance of cervical cancer in LMICs [10-13].

In Ethiopia, cervical cancer is the most common cause of cancer death among female cancers with an overall mortality of 70% [14]. A study in Ethiopia showed that the mean age at diagnosis with cervical cancer was 48 years [15]. In a community based cross-sectional survey conducted in Gondar, northwest Ethiopia (2010), only 31% of respondents were knowledgeable about risk factors, symptoms, treatment options, prevention and early detection measures for cervical

cancer [16]. Studies from Addis Ababa, Adama and Jimma showed that women 40 - 49 years of age, having a history of STIs, having two or more lifetime sexual partners and contraceptive use were risk factors of the cervical pre-cancerous lesion [17- 19]. Another institution based studies in Tigray revealed a prevalence of pre-cancerous cervical lesion of 6.7% and that STIs are predictors of the pre-cancerous cervical lesions [20, 21].

The Ethiopian Ministry of Health officially launched guideline for cervical cancer prevention and control in 2005, describes a “See and Treat approach” using visual inspection with acetic acid (VIA) screening and cryotherapy treatment for the reason of feasibility and affordability (22).

Although Ethiopia’s federal ministry of health (FMoH) has given priority for cervical cancer prevention and control, there is a scarcity of data related to pre-cancerous cervical lesions. Existing guidelines call for community based pre-cancerous cervical lesions epidemiology and risk factors [22]. Accordingly, this community-based research determines the prevalence and factors associated with pre-cancerous cervical lesions among adult women in Tigray, Ethiopia.

Materials and Methods:

Study setting and design

We conducted a community-based, cross-sectional study in Tigray, Northern Ethiopia. Data were collected from March 2016 to June 2017. The study was conducted in Tigray region, which has seven zones, 34 rural and 18 urban districts ('Woredas' in local name) and 763 sub-districts ('Kebele/Tabia' in local name). In 2017, the Tigray Region had one specialized hospital, 14 general hospitals, 22 primary hospitals, 214 health centers, and 712 health posts as well as more than 500 private health facilities.

Study Population

Study participants: All adult women living in Tigray region for about six months or more were considered as a source population, and those who were voluntary and selected as study subjects were the study population.

Eligibility criteria*:* All adult women, who were sexually active and voluntarily consented to participate were included in the study. Those with history of hysterectomy were excluded from the study.

Sample size and sampling technique:

The sample size was calculated with the aim of estimating a precise population prevalence of pre-cancerous cervical lesions in the Tigray Region. As there is no baseline study of prevalence of pre-cancerious cervical lesion, we assumed a 0.5 probability of the disease, 0.05 probability of alpha testing, 80% power and design effect of 2 accounting for two-stage sampling. Previous studies, the response rate ranges from 77.1% to 98.2%, which implies a 1.8% to 2.9.9%

contingency(24, 25). We added 10% to the calculated sample size to account for non-responses, yielding a sample size of 846, which we rounded to 900 for convenience.

A multistage sampling was used to select the study subjects. Considering 40% of the 52 districts in the region, 21 of the districts which were distributed proportionally to zones were selected randomly. Since there are 315 sub-districts in the selected districts, 20% of the sub-districts (a total of 63 sub-districts) were selected randomly. Study participants were allocated proportionally to sub-districts based on the number of participants fulfilling the eligibility criteria in each sites. This was taken from the health extension workers registration book.

Recruitment process: A large mobilization was preceded the screening. This effort entailed training of Health Extension Workers (HEWs) from selected sub-districts. HEWs in turn oriented women in their communities. They also mobilized Women Development Army (WDA) leaders and members as well as sub-district leaders by providing brief orientations about cervical cancer as well as upcoming screening dates and locations. Grassroots mobilization was complimented by promotion on regional radio stations by the Tigray Regional Health Bureau (TRHB).

Following community mobilization, participants were invited for screening to a total of 17 primary and general hospitals in the sub districts which have cervical cancer screening cencers using visual inspection with acetic acid (VIA). As per the estimated number of expected participants, a key value of 4 was calculated. Every forth women arriving at the hospitals following grassroots mobilization were selected and invited to participate in the study. Finally, those who fulfill the eligibility criteria were recruited as a study subject.

Data collection procedure:

Women providing consent were interviewed using an interviewer administered questionnaire to obtain socio-demographic and risk-factors information, including health related behaviors and lifestyle, knowledge, attitude, and clinical attributes.

Clinical testing was conducted by certified nurses trained by the TRHB according to the national cervical cancer prevention guidelines [23]. A VIA screening protocol was developed based on the national guideline, The protocol included private exam area, examination table, trained health professionals, adequate light source, sterile vaginal speculum, new examination or surgical gloves, large cotton swabs, diluted (3-5%) acetic acid, small bowl, containers with 0.5% chlorine solution, plastic bucket with a plastic bag and quality assurance system to maximize accuracy.

Pre-cancerous cervical lesions was determined by vaginal speculum exam during which provider applied diluted acetic acid to the cervix. Visual confirmation of color change by the naked eye was used to determine the presence or absence of lesions. The test was considered positive if a sharp, distinct, well-defined, dense (opaque/dull or oyster) Aceto-white area was present with raised margins touching the Squamo-columnar junction (SCJ). The test was considered negative if no or faint Aceto-white lesions and/or smooth surface was present. The test was cinsudered suspicious for cervical cancer where clinically visible ulcerative, cauliflower-like growth or ulcer, oozing, and/or bleeding on touch were observed.

The history of sexually transmited infection (STI) was determined if any one of the sign and symptoms (discharge, offensive secretion, itching, dysuria, lower abdominal pain, and fever) were present. The variable 'history of cervical cancer screening' was not included in our study, since most of our sites did not have the screening modality previously and we may get no participants with previous history of cervical cancer screening.

Data analysis

Data were analyzed using statistical analysis (STATA) version14. Data entry and clearance was done using descriptive analysis to check if any missing and inappropriate categories. Descriptive statistics were presented in texts and tables. The dependent variable was pre-cancerous cervical lesion'status, in which the screening result had three possible outcomes: Negative, Positive and Suspicious for VIA. A multinomial regression model was used to check the influence of socio-demographic, risk factors and reproductive health characteristics on pre-cancerous cervical lesion 'status. Unadjusted multinomial regression was used to examine the separate bivariate relationship between each independent variables and the dependent variable. The variables with p-value < 0.05 in the unadjusted multinomial regression was considered as significant and used in the multivariable multinomial logistic regression. Relative risk ratios (rrr) were used to assess the multivariate association of the predictor variables with the dependent variable.

Ethical consideration: Before beginning data collection, ethical clearance was obtained from Mekelle University, Collage of Health Siences Research and Community Service Committee (Registration number – ERC 0597/2015) and support letter was obtained from Tigray regional health bureau. Participants were oriented the general objective of the study, procedure of data collection, privacy and confidenciality of the information obtained, it was also informed that participation is voluntary based and the possibility to withdraw at any time when they need. Finally written informed consent was obtained from the participants prior to the actual data collection.

Results

Socio-demographic characteristics of the study participants

A total of 883 questionnaires was collected with complete information, equating to a response rate of 98.1%. More than half of the participants 496 (56.2%) came from urban districts. Mean age of participants was 35.69 years (standard deviation (SD) ±8.45, range 16 - 62). Among all respondents, 532 (60.2%) were married, 723 (81.9%) were Orthodox Christian, 286 (32.4%) had no formal education, 282 (31.9%) had no formal work, and 717 (81.2%) had zero to four children. Median income was Ethiopian Birr 1,500 and 515 (58.3%) women earned between Ethiopian Birr 0 and 2,000 per month [Table 1].

Table 1. Distribution of Socio-demographic characteristics by pre-cancerous cervical lesion status among women of reproductive age in Tigray, (n=883).

**Socio-demographic Variables**	**Groups**	**VIA results**			**Total** N (%)
		**Negative** N (%)	**Positive** N (%)	**Suspicious** N (%)	
Residence	Rural	345(89.1)	28(7.2)	14(3.6)	387(43.8)
	Urban	424(85.5)	51(10.3)	21(4.2)	496(56.2)
Age (yr)	15-30	257(86.5)	30(10.1)	10(3.4)	297 (33.6)
	31-45	416(87.6)	44(9.3)	15(3.2)	475 (53.8)
	>=46	96(86.5)	5(4.5)	10(9.0)	111 (12.6)
Marital status	Single	208(92.0)	14(6.2)	4(1.8)	226(25.6)
	Married	472(88.7)	42(7.9)	18(3.4)	532(60.2)
	Divorced/Others	89(71.2)	23(18.4)	13(10.4)	125(14.2)
Religion	Orthodox	627(86.7)	64(8.9)	32(4.4)	723(81.9)
	Muslim and others	142(88.8)	15(9.4)	3(1.9)	160(18.1)
Educational status	No formal education	240(83.9)	25(8.7)	21(7.3)	286(32.4)
	Primary education	325(89.0)	30(8.2)	10(2.7)	365(41.3)
	Secondary education	116(85.3)	17(12.5)	3(2.2)	136(15.4)
	Tertiary education	88(91.7)	7(7.3)	1(1.0)	96(10.9)
Occupation	Civil servant	209(93.3)	11(4.9)	4(1.8)	224(25.4)
	Farmer	141(81.5)	21(12.1)	11(6.4)	173(19.6)
	Merchant	172(84.3)	18(8.8)	14(6.9)	204(23.1)
	No formal work	247(87.6)	29(10.3)	6(2.1)	282(31.9)
Monthly income	0-2000	439(85.2)	51(9.9)	25(4.9)	515(58.3)
	2001-4000	158(86.3)	16(8.7)	9(4.9)	183(20.7)
	4001-6000	105(92.1)	8(7.0)	1(0.9)	114(12.9)
	6001-8000	16(88.9)	2(11.1)	0(0.0)	18(2.0)
	8001-10000	51(96.2)	2(3.8)	0(0.0)	53(6.0)
Parity	0-4 children	628(87.6)	67(9.3)	22(3.1)	717(81.2)
	5-12 children	141(84.9)	12(7.2)	13(7.8)	166(18.79)

Risk factrs of cervical cancer:

A total of 782 (88.6%) had visible SCJ. Among all the participants, 79 (8.9%) were positive for VIA, 35 (4.0%) were suspicious for cancer, and 769 (87.1%) were negative for pre-cancer lesions. A total of 776 (87.9%) reported that their age at first sexual contact was less than 20 years. Of the total, 344 (39.0%) respondents had more than two sexual partners in their lifetime and 199 (22.5%) experienced STIs. Thirty-two (3.6%) were smokers and one-third of respondents consumed alcohol 255 (28.9%). Corticosteroids were used in 97 (11.0%) women and 199 (22.5%) where current contraceptive users. Seventy (7.9%) had a family history of CC. Out of these women, nine (12.9%) were VIA-positive [Table 2].

Table. 2. Distribution of risk factors by pre-cancerous cervical lesion status among women of reproductive age in Tigray, (n=883).

**Risk factors of cervical cancer**		**VIA results**			**Total** N (%)
		**Negative** N (%)	**Positive** N (%)	**Suspicious** N (%)	
Age at first sexual contact	≤ 20 years age	675(87.0)	66(8.5)	35(4.5)	776(87.9)
	> 20 years age	94(87.9)	13(12.1)	0(0.0)	107(12.1)
Number Sexual Partner	Only one	461(88.3)	45(8.6)	16(3.1)	522(59.1)
	Two or more	293(85.2)	33(9.6)	18(5.2)	344(39.0)
	No or unspecified	15(88.2)	1(5.9)	1(5.9)	17(1.9)
History of STI	Yes	156(78.4)	32(16.1)	11(5.5)	199(22.5)
	No	613(89.6)	47(6.9)	24(3.5)	684(77.5)
Active smoking	Yes	29(90.6)	2(6.2)	1(3.1)	32(3.6)
	No	740(87.0)	77(9.0)	34(4.0)	851(96.4)
Alcohol consumption	Yes	232(91.0)	18(7.1)	5(2.0)	255(28.9)
	No	537(85.5)	61(9.7)	30(4.8)	628(71.1)
Chronic corticosteroids use	Yes	86(88.7)	8(8.2)	3(3.1)	97(11.0)
	No	683(86.9)	71(9.0)	32(4.1)	786(89.0)
Current contraceptive use	Yes	169(84.9)	21(10.6)	9(4.5)	199(22.5)
	No	600(87.7)	58(8.5)	26(3.8)	684(77.5)
Family history of cervical cancer	Yes	60(85.7)	9(12.9)	1(1.4)	70(7.9)
	No	709(87.2)	70(8.6)	34(4.2)	813(92.1)

NB: *STI – sexually transmitted infection*

Signs and symptoms of Cervical Cancer:

From the study participants, 183 (20.7%) women experienced bleeding between menstruation, 94 (10.6%) experienced heavy menstruation, 176 (19.9%) experienced contact bleeding and 152

(17.2%) women experienced pain during sex. Pelvic or back pain was reported by 267 (30.2%) women [Table 3].

Table 3. Distribution of signs and symptoms by pre-cancerous cervical lesion status among women of reproductive age in Tigray, (n=883).

**Sign and symptoms and cervical cancer status**		**VIA results**			
		**Negative** N (%)	**Positive** N (%)	**Suspicious** N (%)	**N (%)**
Bleeding between menstruation	Yes	149(81.4)	16(8.7)	18(9.8)	183(20.7)
	No	620(88.6)	63(9.0)	17(2.4)	700(79.3)
Heavy menstruation	Yes	70(74.5)	12(12.8)	12(12.8)	94(10.6)
	No	699(88.6)	67(8.5)	23(2.9)	789(89.4)
Bleeding after menopause	Yes	78(77.2)	14(13.9)	9(8.9)	101(11.4)
	No	691(88.4)	65(8.3)	26(3.3)	782(88.6)
Contact bleeding	Yes	143(81.2)	19(10.8)	14(8.0)	176(19.9)
	No	626(88.5)	60(8.5)	21(3.0)	707(80.1)
Pain during sex	Yes	120(78.9)	23(15.1)	9(5.9)	152(17.2)
	No	649(88.8)	56(7.7)	26(3.6)	731(82.8)
Pelvic or back pain	Yes	213(79.8)	33(12.4)	21(7.9)	267(30.2)
	No	556(90.3)	46(7.5)	14(2.3)	616(69.8)
Unusual vaginal discharge	Yes	239(79.9)	37(12.4)	23(7.7)	299(33.9)
	No	530(90.8)	42(7.2)	12(2.1)	584(66.1)

Factors associated with pre-cancerous Cervical Lession:

In the multinomial analysis “Negtive result of the dependent variable was used as a base reference against the positive and suspicious results; hence the relative risk is relative to those who have negative results.

Multinomial bivariate analysis revealed that age, marital and education status, occupation, symptoms of cervical cancer, parity, STI history of partner, and lower abdominal pain were significantly associated with positive or suspicious results (p-value < 0.05). Other variables were not significantly associated with pre-cancerous cervical lesion’. The multivariate logistic regression analysis showed that marital and educational status, occupation, history of STIs, bleeding between menstruation, and pelvic or back pain were significantly associated with the presence of pre-cancerous cervical lesion’. Divorced or widowed women were 2.5 and 4.7 times

more likely, to be VIA-positive and suspicious respectively than single women (rrr = 2.50, 95% confidence interval (CI) [1.13, 5.52]); (rrr=4.69, 95% CI [1.00, 21.84]). The risk of being suspicious was 68% lower for women with primary education than those with no formal education (rrr=0.32, 95% CI [1.00, 21.84]). The risk of being suspicious for cancer was 4.8 times higher among farmers (rrr=4.83, 95% CI [1.44, 16.13]) and merchants (rrr=4.85, 95% CI [1.52, 15.46]) than respondents with no formal work.

The risk of being VIA-positive was 90% higher for women with a history of STIs (rrr=1.91, 95% CI [1.11, 3.27]) than their counter parts. The risk of being suspicious for cancer was 3.26 times higher for women who experienced bleeding between periods (rrr =3.26, 95% CI [1.32, 8.04]) than their counter parts. Women with a history of pelvic or back pain were 2.7 times more likely to be suspicious for cancer (rrr =2.79, 95% CI [1.18, 6.58]) [Table 4] than their counter parts.

Table.4. Bivariate and Multivariable analysis of independent variables with pre-cancerous cervical lesion status among reproductive age women in Tigray, (n=883).

**Variables**			**VIA results**					
		**Negative**	**Positive**			**Suspicious**		
		N(%)	***N(%)***	***cRRR [95% CI]***	***aRRR [95% C.I.)***	***N(%)***	***cRRR [95% CI]***	***aRRR [95% C.I.)***
Age	15-30	257(86.5)	30(10.1)	1	1	14(3.6)	1	1
	31-45	416(87.6)	44(9.3)	0.91 (0.56, 1.48)	0.50(0.17, 1.45)	21(4.2)	0.93(0.410, 2.09)	2.34(0.74, 7.36)
	>=46	96(86.5)	5(4.5)	0.45(0.17, 1.18)	0.91(0.53, 1.55)	10(3.4)	2.68(1.08, 6.63) *	0.58(0.22, 1.55)
Marital status	Single	208(92.0)	14(6.2)	1	1	4(1.8)	1	1
	Married	472(88.7)	42(7.9)	1.32(0.71, 2.47)	0.94(0.46, 1.91)	18(3.4)	1.98(0.66, 5.93)	2.15(0.51, 8.97)
	Divorced/widowed	89(71.2)	23(18.4	3.84(1.89, 7.80)***	2.50(1.13, 5.52)*	13(10.4	7.59(2.41, 23.93)***	4.69(1.00, 21.84)*
Educational status	No formal education	240(83.9)	25(8.7)	1	1	21(7.3)	1	1
	Primary Education	325(89.0)	30(8.2)	0.89(0.51, 1.55)	0.78(0.41, 1.48)	10(2.7)	0.35(0.17, 0 .76) ***	0.32(0.11, 0.91)*
	Secondary Education	116(85.3)	17(12.5)	1.41(0.73, 2.71)	1.15(0.54, 2.43)	3(2.2)	0.29(0.09, 1.01)	0.25(0.05, 1.09)
	Tertiary Education	88(91.7)	7(7.3)	0.76(0.32, 1.83)	0.84(0.29, 2.39)	1(1.0)	0.13(0.02, 0.98)*	0.12(0.01, 1.41)
Occupation	Civil Servant	209(93.3)	11(4.9)	0.45(0.22, 0.92)*	0.78(0.41, 1.48)	4(1.8)	0.78(0.22, 2.82)	2.67(0.48, 14.68)
	Farmer	141(81.5)	21(12.1)	1.26(0.69, 2.30)	1.15(0.54, 2.43)	11(6.4)	3.21(1.16, 8.87)*	4.83(1.44, 16.13)*
	Merchant	172(84.3)	18(8.8)	0.89(0.47, 1.65)	0.84(0.29, 2.39)	14(6.9)	3.35(1.26, 8.89)*	4.85(1.52, 15.46)**
	No formal work	247(87.6)	29(10.3)	1	1	6(2.1)	1	1
Parity	0-4 children	628(87.6)	67(9.3)	1	1	22(3.1)	1	
	5-12 children	141(84.9)	12(7.2)	0.79(0.42, 1.51)	0.85(0.40, 1.79)	13(7.8)	2.63(1.29, 5.35)**	1.62(0.61, 4.25)
History of STI	Yes	156(78.4)	32(16.1)	2.67(1.65, 4.33)***	1.91(1.11, 3.27)*	11(5.5)	1.80(0.86, 3.75)	0.99(0.40, 2.42)
	No	613(89.6)	47(6.9)	1	1	24(3.5)	1	1
Blood between period	Yes	149(81.4)	16(8.7)	1.05(0.59, 1.88)	0.71(0.35, 1.45)	18(9.8)	4.40(2.21, 8.75)***	3.26(1.32, 8.04)*
	No	620(88.6)	63(9.0)	1	1	17(2.4)	1	1
Heavy menstruati[illegible]	Yes	70(74.5)	12(12.8)	1.78(0.92, 3.46)	1.23(0.55, 2.74)	12(12.8)	5.20(2.48, 10.9)***	2.45(0.92, 6.52)
	No	699(88.6)	67(8.5)	1	1	23(2.9)	1	1
Bleeding after [illegible]	Yes	78(77.2)	14(13.9)	1.90 (1.02, 3.55)*	1.25(0.57, 2.71)	9(8.9)	3.06 (1.38, 6.77) **	1.27(0.40, 3.99)
	No	691(88.4)	65(8.3)	1	1	26(3.3)	1	1
Contact bleeding	Yes	143(81.2)	19(10.8)	1.38(0.80, 2.39)	0.85(.42, 1.69)	14(8.0)	2.91(1.44, 5.87)**	0.81(0.30, 2.19)
	No	626(88.5)	60(8.5)	1	1	21(3.0)	1	1
Pain during sex	Yes	120(78.9)	23(15.1)	2.22(1.31, 3.74)**	1.39(0.70, 2.74)	9(5.9)	1.87(0.85, 4.09)	0.69(0.23, 2.01)
	No	649(88.8)	56(7.7)	1	1	26(3.6)	1	1
Pelvic or back pain	Yes	213(79.8)	33(12.4)	1.87(1.16, 3.00)*	1.60(0.90, 2.85)	21(7.9)	3.91(1.95, 7.84)***	2.79(1.18, 6.58)*
	No	556(90.3)	46(7.5)	1	1	14(2.3)	1	1
Unusual vagina [illegible]	Yes	239(79.9)	37(12.4)	1.95(1.22, 3.11)**	1.39(0.78, 2.49)	23(7.7)	4.26 (2.08, 8.68)***	2.22(0.91, 5.38)
	No	530(90.8)	42(7.2)	1	1	12(2.1)	1	1

NB: *P - value < 0.001 = ***, 0.001 - 0. 009 = ** and 0. 010 - 0. 05 = * and cRRR – crude relative risk ratio, aRRR - Adjusted relative risk ratio, C.I. - Confidence Interval*

Discussion

Ethiopia's Federal Ministry of Health has prioritized cervical cancer prevention and treatment [23]; however, there is a scarcity of data related to the prevalence of pre-cancerous cervical lesions. This study explored the prevalence of pre-cancerous cervical lesions in Tigray, Northern Ethiopia.

Our study showed that 79 (8.9%) respondents were VIA-positive and 35 (4.0%) were suspicious for cervical cancer. The overall prevalence of VIA-positive was higher in this study than a study at Alameda Textile Factory in Tigray, which showed a VIA-positive rate of 6.7% [20]. A study at a Family Guidance Association (FGA) Clinic in Jimma, southern Ethiopia, showed that 12.9% of women were VIA-positive [19]. Studies in Swaziland, Cameroon, and Jakarta, Indonesia, showed VIA-positive rates of 15%, 3.33%, and 4.7%, respectively [10, 11, 12]. Our study also showed that 4.0% of women were suspicious for cancer compared to 1.2% of women in the study done in Jimma [19]. Though most are similar, the difference might be because of the skill difference of the care providers, and type acetic acid used for screening.

After adjusting for other variables, marital and educational status, occupation, history of STIs, bleeding between period, and pelvic or back pain were statistically significant risk factors for VIA-positive or suspicious for cancer.

Divorced or widowed women had a risk of being VIA-positive (p=0.023) and being suspicious (p=0.049) for cervical cancer than single women. A study done in Dar Es Salaam, Tanzania, showed thatdivorced women were more likely to be VIA positive [13], which is similar to this study. A study in Jakarta, Indonesia, revealed that the overall VIA positive rate was 4.7%; of which, 8.3% were married more than one timesin their life [12]. This implies that women who have started sexual practice and have divorced, widowed or separated and has no permanent

husband had a chance of exposing to multiple sexual partner; which increases the risk of exposure to HPV. Increasing outreach and screening intensity to divorced, separated, and widowed women could be a simple approach to streamlining the identification and treatment of women at risk for developing cervical cancer.

Women with primary education were approximately 70% less likely to be suspicious for cervical cancer (p=0.034) than women with no education, and education was not significantly correlated with VIA positivity. What's more, higher education levels, including secondary and tertiary, were not significantly associated with VIA-positive or suspicious. Similar to our study, a research findings from Dar Es Salaam, showed that women with low education had odds 4.3 higher of being VIA positive [13],. Studies in Gondar, Ethiopia, and India also showed that lack of awareness on cervical cancerr was a risk factor for cervical cancer [7,16]. This might be that increasing awareness including educational level could influence individuals to prevent themselves from any risk factors of cervical cancer.

In this study,women who are farmers and merchants in occupation had odds of 4.8 times higher of being suspicious for cervical cancer. Farmers may have less awareness of cervical cancer and lack of ability to participate in the early screening of cervical cancer. In the case of merchants, they could have the opportunity to visit different areas and stay out of houses. This may expose them to heightened risk due to increased chance of sexual practices that expose them to HPV.

Respondents with a history of STI had 1.91 times the risk of being VIA-positive (p=0.018), and though with history of STIs were not significantly associated with suspicious results. This was similar to studies done in Addis Ababa, Adama and Alamed Textile Factory, which revealted thatthe history of STI was a risk factor for pre-cancerous cervical lesion[17, 18, 20]. This could

be that women with STIs are more prone to aquire HPV infection, which is by far the dominant pathway for cervical cancer to initiate and progress.

Bleeding between period and pelvic or back pain were significantly associated with suspicious cance, which were 3.26 (p=0.010) and 2.79 (p=0.019) times higher risk respectively than their counter parts. Which was similar to a study done in Uganda revealed that the participants recognized inter-menstrual bleeding as a symptom of cervical cancer [9]. This might be mainly related to suspicious for cervical cancer;as the lesion advanced, the common sign and symptom are vaginal bleeding and pain.

In this study, 9.0% of 46 years or older women, were found suspicious for cervical cancer compared to approximately 9.5% for women less than 46 years of age. Age was not statistically significant.. Unlike this study, a study in India revealed that women 55 to 59 years of age had the highest risk of cervical cancer [8], this was also observed in one of the studies in Ethiopian[15]. This may implies that Ethiopian women may have earlier sexual initiation than Indian women that delaying introduction and progression of the disease to pre-cancerous cervical lesion.

In thisstudy, the presence of pre-cancerous cervical lesion'was not associated with current contraceptive use or age at first sexual contact. Unlike to this study, the age at first sexual contact was significant risk factor for pre-cancerous cervical lesion'in a study done in Jimma [19]. Unlike to this study, a research findings done in Jakarta, Addis Ababa and Adama, Uganda, and India showed that contraceptive use was a risk factor for pre-cancerous cervical lesions [9,8, 12,17,18]. Logically, long term contraceptive uses might be the risk factor for cervical cance. Hence, it might be because of that there are no more long time contraceptive users in the study area, which is evidenced by having high fertility rate. This might be preventive to pre-cancerous cervical lesion.

Strenngth and limitation of the study:

The primary data of pre-cancerous cervical lesion'was acertaned using VIA screening, rather than history taking. This method of data collection minimizes recall bias. However a case control study design could better estimate the association of independent variables with the outcome variable. This study did not include variables that have probability of being risk factors like HIV status and history of cervical cancer screening because of the absence of the services in some sites.

Conclusion

In this study, 8.9% and 4% of the women were positive and suspicious for pre-cancerous cervical lesion, respectively. The prevalence of pre-cancerous cervical lesion is high as compared to other regional prevalence in the country. Marital status, educational status, occupation, history of an STI, bleeding between period, and pelvic or back pain were risk factors for pre-cancerous cervical lesion. This also implies that cervical cancer prevention is better to be strengthened focusing on preventable risk factors, including exposure to sexually transmitted infections and specific vulnerable groups like divorced and widowed women. Further study of the age distribution of pre-cancerous cervical lesions is merited.

List of Abbreviations:

aRRR – Adjusted Relative Risk Ratio

cRRR - Crude Relative Risk Ratio

FMoH - Ethiopia's federal ministry of health

HEWs - Health Extension Workers

HIC - High Income Countries

HIV - Human Immunodeficiency Virus

HPV - Human Papilloma Virus

LMICs - low-and-middle income countries

RRR - Relative Risk Ration

SCJ - Squamous-Columnar Junction

SD - Standard Deviation

STATA - STATistical Analysis

STI - Sexually Transmitted Infection

TRHB - Tigray Regional Health Bureau

VIA - Visual Inspection With Acetic acid

WDA - Women Development Army

Declarations

Ethics Approval and Consent to Participate:

The study protocol was evaluated and approved by the Research Ethics Review Committee of College of Health Sciences, Mekelle University. Official cooperation letters were obtained from Mekelle University, Tigray Regional Health Bureau, and zonal health offices. Moreover, the purpose, advantage and disadvantage of the study were described to the study participants. The consent involves permission to disseminate the findings of the study through scientific workshop presentations and publications. The study participants were told that they have full right to discontinue the study. Confidentiality and any special data security requirements were maintained and assured. The participants were informed that results of the screening that have a direct benefit to their health would be informed to physicians working in the nearby health facility. Finally written informed consent was obtained from the participants prior to the actual data collection.

Consent for publications: Not applicable

Data availability: All the data sets used and/or analyzed during the current study are included in the manuscript. Data set has been uploaded with this revised documents.

Authors' contribution

GBA, HY, SN and HH involved during the conception and designing of the study. GBA, SN, HH carried out data collection and supervision of the data collection. GBA, HY, SN and HH performed statistical analysis. YB, GG1, AT and GG2 were participated in following, editing, recommending and providing constructive comments till finalization of this manuscript. All authors read and approved the final manuscript.

Authors' information:

GBA is an instructor and researcher as associate professors in maternity and reproductive health, at College of Health sciences, Mekelle

YB is a physician gynecologist and instructor as associate professors and researchers at College of Health sciences, Mekelle University;

AT is a microbiologist instructor, and Presidant of the University and PhD holder as associate professor and researcher at college of Health Sciences, Axum University;

SN and HH are a microbiologist instructors as assistant professors and researchers at College of health Sciences, Mekelle University;

HY is an instructor, assistant professor in biostatistics and researcher in Public Health, College of Health Sciences, Mekelle University. Currently living in Germen.

GG1 is a public health in professiona and currently deputy general director of FMHACA, Addis Ababa

GG2 is a Female physician GYNOBS and global women fellowship program director at University of Illinoise, Chicago.

Competing Interests: The authors have no competing interests to declare.

Funding: Tigray Regional Health Bureau and Mekelle University funded the data collection prosess. “The author(s) did not receive any funding for this paperwork or manuscript publication from any source." “The Mekelle University had no role in study design, data collection and analysis, decision to publish, or preparation of the manuscript.”

ORCID iD https://orcid.org/0000-0003-4968-2885

Acknowledgments:

We aknowledge the Tigray Regional Health Bureau and Mekelle University for their support in the data collection process. Our great appreciation also goes to healthcare workers of the health facilities involved in the data collection and all study participants of the study for their commitment and geniun participation. Moreover we would like to extend our acknowledgment for administrative and community health care workers for their support during data collection and facilitation of the project.

References

1. A report from WHO. A demonstration project, Prevention of cervical cancer through screening using visual inspection with acetic acid (VIA) and treatment with Cryotherapy.African population and health research center, 2012.
2. American cancer society report, a guideline for cervical cancer and its managements. www.cancer.org Last Medical Review: 4/11/2013 and Last Revised: 1/31/2014.
3. The Alliance for Cervical Cancer Prevention (ACCP), inspection with acetic acid (VIA): Evidence to date. www.alliance-cxca.org.
4. Data derived from official reports by WHO, United Nations, Cervical Cancer Global Crisis Card.The World Bank, IARC Globocan. http://www.who.int/hpvcentre/statistics/en/.
5. Campbell C. Assessment of Cervical Cancer Incidence, Histopathology, and Screening Practices Among Hispanic Women in Latin America and Michigan. A dissertation for the degree of Doctor of Philosophy, 2011.
6. Zohre Momenimovahed and Hamid Salehiniya, Incidence, mortality and risk factors of cervical cancer in the world. Biomedical Research and Therapy, BioMedPress, 2018, http://www.bmrat.org/index.php/BMRAT/article/view/386.
7. Rose I Anorlu, Cervical cancer: the sub-Saharan African perspective. Reproductive Health Matters, 2008;16(32).
8. Aswathy Sreedevi, Reshma Javed, and Avani Dinesh, Epidemiology of cervical cancer with special focus on India. Int J Womens Health. 2015; 7: 405–414, doi: 10.2147/IJWH.S50001.
9. Amos D. Mwaka, Christopher G. Orach, Edward M. Were, Georgios Lyratzopoulos, Henry Wabinga, Martin Roland, Awareness of cervical cancer risk factors and symptoms: cross-sectional community survey in post-conflict northern Uganda. Health Expect. 2016 Aug; 19(4): 854–867, doi: 10.1111/hex.12382.
10. Ginindza TG, Almonte M, Dlamini X, Sartorius B. Distribution of cervical abnormalities detected by visual inspection with acetic acid in Swaziland, 2011-2014: A retrospective study. African J Prim Heal care Fam Med. 2018;10(1):e1-e7, doi:10.4102/phcfm.v10i1.1773.

11. Nkfusai NC, Mubah TM, Yankam BM, Tambe TA, Cumber SN. Prevalence of precancerous cervical lesions in women attending Mezam Polyclinic Bamenda, Cameroon. Pan Afr Med J. 2019; 32:174. doi:10.11604/pamj.2019.32.174.16895.
12. Anggraeni TD, Peters AAW. Prevalence , Age Distribution , and Risk Factors of Visual Inspection With Acetic Acid-Positive From 2007 to 2011 in Jakarta. 2017;22(2):103-107.
13. Kahesa C, Kjaer SK, Ngoma T, et al. Risk factors for VIA positivity and determinants of screening attendances in Dar es Salaam , Tanzania. BMC Public Health. 2012; 12(1):1. doi:10.1186/1471-2458-12-1055.
14. Haile T. Beyene M. Berg A. prevention of cervical cancer in Ethiopia, a report from Women´s Health Center, Addis Ababa, Ethiopia and ESOG, Ethiopia, a model clinic in Addis Ababa, 2018.
15. Dye D., Solomon B., Claire H., et al, Complex care systems in developing countries. Breast Cancer Patient Navigation in Ethiopia,2009, 116:577–85.).
16. Birhanu Z. et al, Health seeking behavior for cervical cancer in Ethiopia: a qualitative study, International Journal for Equity in Health, BioMed center, 2012, 11:83.
17. Roza Teshome Kassa, Risk factors associated with precancerous cervical lesion among women screened at Marie Stops Ethiopia, Adama town, Ethiopia: a case control study, BMC Res Notes,2018, 11:145, https://doi.org/10.1186/s13104-018-3244-6.
18. Teame H, Addissie A, Ayele W, Hirpa S, Gebremariam A, Gebreheat G, et al. Factors associated with cervical precancerous lesions among women screened for cervical cancer in Addis Ababa, Ethiopia: A case control studyPLoS ONE, , 2018. 13(1): e0191506. https://doi.org/10.1371/journal.pone.0191506.
19. Deksissa ZM, Tesfamichael FA, Ferede HA. Prevalence and factors associated with VIA positive result among clients screened at Family Guidance Association of Ethiopia, south west area office, Jimma model clinic, Jimma, Ethiopia: A cross-sectional study, Womens Health. BMC Res Notes. 2015; 8(1):8-13. doi:10.1186/s13104-015-1594.
20. Kebede Haile Misgina, Hailay Seyoum Belay and Teklehaymanot Huluf Abraha, Prevalence of precancerous cervical lesion and associated factors among women in North Ethiopia, Journal of Public Health and Epidemiology, 2017, Vol. 9(3), pp. 46-50, DOI: 10.5897/JPHE2016.0891

21. Bayu H, Berhe Y, Mulat A, Alemu A Cervical Cancer Screening Service Uptake and Associated Factors among Age Eligible Women in Mekelle Zone, Northern Ethiopia,: A Community Based Study Using Health Belief Model. PLoS ONE, 2016, 11(3): e0149908. doi:10.1371/journal.pone.0149908
22. Yeneabeba Tilahun, National Cervical Cancer Prevention Training Package Participant Manual, Federal Ministry of Health Ethiopia, 2015.
23. Federal Democratic Republic of Ethiopia Ministry of Health. Guideline for Cervical Cancer Prevention and Control in Ethiopia.; 2015. https://www.iccp-portal.org/system/files/plans/Guideline Eth Final.pdf.
24. Seyoum T, Yesuf A, Kejela G, Gebremeskel F (2017) Utilization of Cervical Cancer Screening and Associated Factors among Female Health Workers in Governmental Health Institution of Arba Minch Town and Zuria District, Gamo Gofa Zone, Arba Minch, Ethiopia, 2016. Arch Cancer Res. Vol.5 No.4:165
25. Cecilia Chinemerem Nwabichie, Rosliza Abdul Manaf*, Suriani Binti Ismail, Factors Affecting Uptake of Cervical Cancer Screening Among African Women in Klang Valley, Malaysia, Asian Pac J Cancer Prev, 19 (3), 825-831

Manuscript
Click here to access/download;Manuscript;2. Manuscript- Feb - 12-2022.doc

Epidemiology of Pre-cancerous Cervical Lesion and Risk Factors among Adult Women n Tigray, Ethiopia

Authors:

Gerezgiher B. Abera[1*], Henock G. Yebyo[2], Haftamu Hailekiros[3], Selam Niguse[3], Yibrah Berhe[3], Goitom Gigar[4], Tsehaye Asmelash[5], Gelila Goba[6]

Affiliation:

[1]School of Nursing, College of Health Sciences, Mekelle University, Mekelle, Ethiopia;

[2]School of Public Health, College of Health Sciences, Mekelle University, Mekelle, Ethiopia;

[3] School of Medicine, College of Health Sciences, Mekelle University, Mekelle, Ethiopia;

[4] Tigray Regional Health Bureau, Mekelle, Ethiopia;

[5]College of Health Sciences, Aksum University, Axum", Ethiopia;

[6]Department of Obstetrics and Gynecology, University of Illinois at Chicago, Chicago, USA

***Corresponding Author**: E-mail: gbamsc2002@gmail.com

ORCID ID https://orcid.org/0000-0003-4968-2885

Abstract

Background: Cervical cancer is a preventable disease if treated early, but remains the second leading cause of cancer-related mortality among women in low and middle-income countries. Data on epidemiology and risk factors in these settings are scarce. This study aimed to assess the prevalence of precancerous lesions and risk factors in Tigray region, Ethiopia.

Methods: This was a community-based, cross-sectional study conducted among 900 women in Tigray region, Ethiopia, from March 2016 to June 2017.Data were collected using an interviewer administered questionnaire and screening with visual inspection with ascetic acid. Multinomial logistic regression analysis was conducted to estimate risk factors.

Results: Of the participants, 883 were responded correctly. Seventy-nine (8.95%) women were positive for pre-cancerous cervical lesion'and 35 (3.96%) were suspicious for cervical cancer. In the multivariable analysis, divorced or widowed women had higher risk of being positive for pre-cancerous cervical lesion'and or suspicious for cancer than single women. The risk of having a suspicious for cancer result was lower for women with primary education than those with no formal education. Participants who had history of sexually transmitted infection were at higher of being positive for pre-cancerous cervical lesion result . Participants who were farmer and merchant in occupation, those who had bleeding between periods and had pelvic or back pain were at risk of having suspicious for cervical cancer results.

Conclusion: About 8.9% and 4% of the women were positive for pre-cancerous cervical lesion'and suspicious for cancer, respectively. Marital status, education, sexually transmitted infection, bleeding, and pelvic pain were risk factors of pre-cancerous cervical lesion'.

Key words: Epidemiology, Cervical, Pre-cancerous lesions.

Introduction

Cervical cancer is a public health problem worldwide [1], primarily in low and middle-income countries, though data are scarce [2, 3]. The disease is fatal, but preventable if treated early [4]. The World Health Organization (WHO) indicates that cervical cancer kills an estimated 275,000 women every year and 500,000 new cases reported worldwide [1]. Cervical cancer is the second largest killer among cancer-related death of women in low-and-middle income countries (LMICs) [4]. Reports from different countries showed that the incidence of cervical cancer is high in developing countries and sexually transmitted infections (STIs), hormonal influences, genetics and participant factors (risk taking behavior, substance abuse, alcohol use) are risk factors for cervical cancer [5, 6]. In Sub-Saharan Africa, an estimated 57,000 cases of cervical cancer occurred in 2000, comprising 22.2% of all female cancers [7].

A study from India revealed that late-stages reporting of disease was common with a peak age of cervical cancer incidence from 55 to 59 years and early marriage, multiple sexual partners, multiple pregnancies, oral contraceptives, and lack of awareness were significant risk factors [8]. A study in Uganda showed that 99% of women had heard about cervical cancer and 63% believed that family planning was a cause of cervical cancer as well as 85% recognizing inter-menstrual bleeding as a symptom of cervical cancer [9]. Other studies have also revealed the burden and public health importance of cervical cancer in LMICs [10-13].

In Ethiopia, cervical cancer is the most common cause of cancer death among female cancers with an overall mortality of 70% [14]. A study in Ethiopia showed that the mean age at diagnosis with cervical cancer was 48 years [15]. In a community based cross-sectional survey conducted in Gondar, northwest Ethiopia (2010), only 31% of respondents were knowledgeable about risk factors, symptoms, treatment options, prevention and early detection measures for cervical

cancer [16]. Studies from Addis Ababa, Adama and Jimma showed that women 40 - 49 years of age, having a history of STIs, having two or more lifetime sexual partners and contraceptive use were risk factors of the cervical pre-cancerous lesion [17- 19]. Another institution based studies in Tigray revealed a prevalence of pre-cancerous cervical lesion of 6.7% and that STIs are predictors of the pre-cancerous cervical lesions [20, 21].

The Ethiopian Ministry of Health officially launched guideline for cervical cancer prevention and control in 2005, describes a "See and Treat approach" using visual inspection with acetic acid (VIA) screening and cryotherapy treatment for the reason of feasibility and affordability (22).

Although Ethiopia's federal ministry of health (FMoH) has given priority for cervical cancer prevention and control, there is a scarcity of data related to pre-cancerous cervical lesions. Existing guidelines call for community based pre-cancerous cervical lesions epidemiology and risk factors [22]. Accordingly, this community-based research determines the prevalence and factors associated with pre-cancerous cervical lesions among adult women in Tigray, Ethiopia.

Materials and Methods:

Study setting and design

We conducted a community-based, cross-sectional study in Tigray, Northern Ethiopia. Data were collected from March 2016 to June 2017. The study was conducted in Tigray region, which has seven zones, 34 rural and 18 urban districts ('Woredas' in local name) and 763 sub-districts ('Kebele/Tabia' in local name). In 2017, the Tigray Region had one specialized hospital, 14 general hospitals, 22 primary hospitals, 214 health centers, and 712 health posts as well as more than 500 private health facilities.

Study Population

Study participants: All adult women living in Tigray region for about six months or more were considered as a source population, and those who were voluntary and selected as study subjects were the study population.

Eligibility criteria: All adult women, who were sexually active and voluntarily consented to participate were included in the study. Those with history of hysterectomy were excluded from the study.

Sample size and sampling technique:

The sample size was calculated with the aim of estimating a precise population prevalence of pre-cancerous cervical lesions in the Tigray Region. As there is no baseline study of prevalence of pre-cancerious cervical lesion, we assumed a 0.5 probability of the disease, 0.05 probability of alpha testing, 80% power and design effect of 2 accounting for two-stage sampling. Previous studies, the response rate ranges from 77.1% to 98.2%, which implies a 1.8% to 2.9.9%

contingency(24, 25). We added 10% to the calculated sample size to account for non-responses, yielding a sample size of 846, which we rounded to 900 for convenience.

A multistage sampling was used to select the study subjects. Considering 40% of the 52 districts in the region, 21 of the districts which were distributed proportionally to zones were selected randomly. Since there are 315 sub-districts in the selected districts, 20% of the sub-districts (a total of 63 sub-districts) were selected randomly. Study participants were allocated proportionally to sub-districts based on the number of participants fulfilling the eligibility criteria in each sites. This was taken from the health extension workers registration book.

Recruitment process: A large mobilization was preceded the screening. This effort entailed training of Health Extension Workers (HEWs) from selected sub-districts. HEWs in turn oriented women in their communities. They also mobilized Women Development Army (WDA) leaders and members as well as sub-district leaders by providing brief orientations about cervical cancer as well as upcoming screening dates and locations. Grassroots mobilization was complimented by promotion on regional radio stations by the Tigray Regional Health Bureau (TRHB).

Following community mobilization, participants were invited for screening to a total of 17 primary and general hospitals in the sub districts which have cervical cancer screening cencers using visual inspection with acetic acid (VIA). As per the estimated number of expected participants, a key value of 4 was calculated. Every forth women arriving at the hospitals following grassroots mobilization were selected and invited to participate in the study. Finally, those who fulfill the eligibility criteria were recruited as a study subject.

Data collection procedure:

Women providing consent were interviewed using an interviewer administered questionnaire to obtain socio-demographic and risk-factors information, including health related behaviors and lifestyle, knowledge, attitude, and clinical attributes.

Clinical testing was conducted by certified nurses trained by the TRHB according to the national cervical cancer prevention guidelines [23]. A VIA screening protocol was developed based on the national guideline, The protocol included private exam area, examination table, trained health professionals, adequate light source, sterile vaginal speculum, new examination or surgical gloves, large cotton swabs, diluted (3-5%) acetic acid, small bowl, containers with 0.5% chlorine solution, plastic bucket with a plastic bag and quality assurance system to maximize accuracy.

Pre-cancerous cervical lesions was determined by vaginal speculum exam during which provider applied diluted acetic acid to the cervix. Visual confirmation of color change by the naked eye was used to determine the presence or absence of lesions. The test was considered positive if a sharp, distinct, well-defined, dense (opaque/dull or oyster) Aceto-white area was present with raised margins touching the Squamo-columnar junction (SCJ). The test was considered negative if no or faint Aceto-white lesions and/or smooth surface was present. The test was cinsudered suspicious for cervical cancer where clinically visible ulcerative, cauliflower-like growth or ulcer, oozing, and/or bleeding on touch were observed.

The history of sexually transmited infection (STI) was determined if any one of the sign and symptoms (discharge, offensive secretion, itching, dysuria, lower abdominal pain, and fever) were present. The variable 'history of cervical cancer screening' was not included in our study, since most of our sites did not have the screening modality previously and we may get no participants with previous history of cervical cancer screening.

Data analysis

Data were analyzed using statistical analysis (STATA) version14. Data entry and clearance was done using descriptive analysis to check if any missing and inappropriate categories. Descriptive statistics were presented in texts and tables. The dependent variable was pre-cancerous cervical lesion'status, in which the screening result had three possible outcomes: Negative, Positive and Suspicious for VIA. A multinomial regression model was used to check the influence of socio-demographic, risk factors and reproductive health characteristics on pre-cancerous cervical lesion 'status. Unadjusted multinomial regression was used to examine the separate bivariate relationship between each independent variables and the dependent variable. The variables with p-value < 0.05 in the unadjusted multinomial regression was considered as significant and used in the multivariable multinomial logistic regression. Relative risk ratios (rrr) were used to assess the multivariate association of the predictor variables with the dependent variable.

Ethical consideration: Before beginning data collection, ethical clearance was obtained from Mekelle University, Collage of Health Siences Research and Community Service Committee (Registration number – ERC 0597/2015) and support letter was obtained from Tigray regional health bureau. Participants were oriented the general objective of the study, procedure of data collection, privacy and confidenciality of the information obtained, it was also informed that participation is voluntary based and the possibility to withdraw at any time when they need. Finally written informed consent was obtained from the participants prior to the actual data collection.

Results

Socio-demographic characteristics of the study participants

A total of 883 questionnaires was collected with complete information, equating to a response rate of 98.1%. More than half of the participants 496 (56.2%) came from urban districts. Mean age of participants was 35.69 years (standard deviation (SD) ±8.45, range 16 - 62). Among all respondents, 532 (60.2%) were married, 723 (81.9%) were Orthodox Christian, 286 (32.4%) had no formal education, 282 (31.9%) had no formal work, and 717 (81.2%) had zero to four children. Median income was Ethiopian Birr 1,500 and 515 (58.3%) women earned between Ethiopian Birr 0 and 2,000 per month [Table 1].

Table 1. Distribution of Socio-demographic characteristics by pre-cancerous cervical lesion status among women of reproductive age in Tigray, (n=883).

**Socio-demographic Variables**	**Groups**	**VIA results**			**Total** N (%)
		**Negative** N (%)	**Positive** N (%)	**Suspicious** N (%)	
Residence	Rural	345(89.1)	28(7.2)	14(3.6)	387(43.8)
	Urban	424(85.5)	51(10.3)	21(4.2)	496(56.2)
Age (yr)	15-30	257(86.5)	30(10.1)	10(3.4)	297 (33.6)
	31-45	416(87.6)	44(9.3)	15(3.2)	475 (53.8)
	>=46	96(86.5)	5(4.5)	10(9.0)	111 (12.6)
Marital status	Single	208(92.0)	14(6.2)	4(1.8)	226(25.6)
	Married	472(88.7)	42(7.9)	18(3.4)	532(60.2)
	Divorced/Others	89(71.2)	23(18.4)	13(10.4)	125(14.2)
Religion	Orthodox	627(86.7)	64(8.9)	32(4.4)	723(81.9)
	Muslim and others	142(88.8)	15(9.4)	3(1.9)	160(18.1)
Educational status	No formal education	240(83.9)	25(8.7)	21(7.3)	286(32.4)
	Primary education	325(89.0)	30(8.2)	10(2.7)	365(41.3)
	Secondary education	116(85.3)	17(12.5)	3(2.2)	136(15.4)
	Tertiary education	88(91.7)	7(7.3)	1(1.0)	96(10.9)
Occupation	Civil servant	209(93.3)	11(4.9)	4(1.8)	224(25.4)
	Farmer	141(81.5)	21(12.1)	11(6.4)	173(19.6)
	Merchant	172(84.3)	18(8.8)	14(6.9)	204(23.1)
	No formal work	247(87.6)	29(10.3)	6(2.1)	282(31.9)
Monthly income	0-2000	439(85.2)	51(9.9)	25(4.9)	515(58.3)
	2001-4000	158(86.3)	16(8.7)	9(4.9)	183(20.7)
	4001-6000	105(92.1)	8(7.0)	1(0.9)	114(12.9)
	6001-8000	16(88.9)	2(11.1)	0(0.0)	18(2.0)
	8001-10000	51(96.2)	2(3.8)	0(0.0)	53(6.0)
Parity	0-4 children	628(87.6)	67(9.3)	22(3.1)	717(81.2)
	5-12 children	141(84.9)	12(7.2)	13(7.8)	166(18.79)

Risk factrs of cervical cancer:

A total of 782 (88.6%) had visible SCJ. Among all the participants, 79 (8.9%) were positive for VIA, 35 (4.0%) were suspicious for cancer, and 769 (87.1%) were negative for pre-cancer lesions. A total of 776 (87.9%) reported that their age at first sexual contact was less than 20 years. Of the total, 344 (39.0%) respondents had more than two sexual partners in their lifetime and 199 (22.5%) experienced STIs. Thirty-two (3.6%) were smokers and one-third of respondents consumed alcohol 255 (28.9%). Corticosteroids were used in 97 (11.0%) women and 199 (22.5%) where current contraceptive users. Seventy (7.9%) had a family history of CC. Out of these women, nine (12.9%) were VIA-positive [Table 2].

Table. 2. Distribution of risk factors by pre-cancerous cervical lesion status among women of reproductive age in Tigray, (n=883).

**Risk factors of cervical cancer**		**VIA results**			**Total** N (%)
		**Negative** N (%)	**Positive** N (%)	**Suspicious** N (%)	
Age at first sexual contact	≤ 20 years age	675(87.0)	66(8.5)	35(4.5)	776(87.9)
	> 20 years age	94(87.9)	13(12.1)	0(0.0)	107(12.1)
Number Sexual Partner	Only one	461(88.3)	45(8.6)	16(3.1)	522(59.1)
	Two or more	293(85.2)	33(9.6)	18(5.2)	344(39.0)
	No or unspecified	15(88.2)	1(5.9)	1(5.9)	17(1.9)
History of STI	Yes	156(78.4)	32(16.1)	11(5.5)	199(22.5)
	No	613(89.6)	47(6.9)	24(3.5)	684(77.5)
Active smoking	Yes	29(90.6)	2(6.2)	1(3.1)	32(3.6)
	No	740(87.0)	77(9.0)	34(4.0)	851(96.4)
Alcohol consumption	Yes	232(91.0)	18(7.1)	5(2.0)	255(28.9)
	No	537(85.5)	61(9.7)	30(4.8)	628(71.1)
Chronic corticosteroids use	Yes	86(88.7)	8(8.2)	3(3.1)	97(11.0)
	No	683(86.9)	71(9.0)	32(4.1)	786(89.0)
Current contraceptive use	Yes	169(84.9)	21(10.6)	9(4.5)	199(22.5)
	No	600(87.7)	58(8.5)	26(3.8)	684(77.5)
Family history of cervical cancer	Yes	60(85.7)	9(12.9)	1(1.4)	70(7.9)
	No	709(87.2)	70(8.6)	34(4.2)	813(92.1)

NB: *STI – sexually transmitted infection*

Signs and symptoms of Cervical Cancer:

From the study participants, 183 (20.7%) women experienced bleeding between menstruation, 94 (10.6%) experienced heavy menstruation, 176 (19.9%) experienced contact bleeding and 152

(17.2%) women experienced pain during sex. Pelvic or back pain was reported by 267 (30.2%) women [Table 3].

Table 3. Distribution of signs and symptoms by pre-cancerous cervical lesion status among women of reproductive age in Tigray, (n=883).

Sign and symptoms and cervical cancer status		VIA results			N (%)
		Negative N (%)	Positive N (%)	Suspicious N (%)	
Bleeding between menstruation	Yes	149(81.4)	16(8.7)	18(9.8)	183(20.7)
	No	620(88.6)	63(9.0)	17(2.4)	700(79.3)
Heavy menstruation	Yes	70(74.5)	12(12.8)	12(12.8)	94(10.6)
	No	699(88.6)	67(8.5)	23(2.9)	789(89.4)
Bleeding after menopause	Yes	78(77.2)	14(13.9)	9(8.9)	101(11.4)
	No	691(88.4)	65(8.3)	26(3.3)	782(88.6)
Contact bleeding	Yes	143(81.2)	19(10.8)	14(8.0)	176(19.9)
	No	626(88.5)	60(8.5)	21(3.0)	707(80.1)
Pain during sex	Yes	120(78.9)	23(15.1)	9(5.9)	152(17.2)
	No	649(88.8)	56(7.7)	26(3.6)	731(82.8)
Pelvic or back pain	Yes	213(79.8)	33(12.4)	21(7.9)	267(30.2)
	No	556(90.3)	46(7.5)	14(2.3)	616(69.8)
Unusual vaginal discharge	Yes	239(79.9)	37(12.4)	23(7.7)	299(33.9)
	No	530(90.8)	42(7.2)	12(2.1)	584(66.1)

Factors associated with pre-cancerous Cervical Lession:

In the multinomial analysis "Negtive result of the dependent variable was used as a base reference against the positive and suspicious results; hence the relative risk is relative to those who have negative results.

Multinomial bivariate analysis revealed that age, marital and education status, occupation, symptoms of cervical cancer, parity, STI history of partner, and lower abdominal pain were significantly associated with positive or suspicious results (p-value < 0.05). Other variables were not significantly associated with pre-cancerous cervical lesion'. The multivariate logistic regression analysis showed that marital and educational status, occupation, history of STIs, bleeding between menstruation, and pelvic or back pain were significantly associated with the presence of pre-cancerous cervical lesion'. Divorced or widowed women were 2.5 and 4.7 times

more likely, to be VIA-positive and suspicious respectively than single women (rrr = 2.50, 95% confidence interval (CI) [1.13, 5.52]); (rrr=4.69, 95% CI [1.00, 21.84]). The risk of being suspicious was 68% lower for women with primary education than those with no formal education (rrr=0.32, 95% CI [1.00, 21.84]). The risk of being suspicious for cancer was 4.8 times higher among farmers (rrr=4.83, 95% CI [1.44, 16.13]) and merchants (rrr=4.85, 95% CI [1.52, 15.46]) than respondents with no formal work.

The risk of being VIA-positive was 90% higher for women with a history of STIs (rrr=1.91, 95% CI [1.11, 3.27]) than their counter parts. The risk of being suspicious for cancer was 3.26 times higher for women who experienced bleeding between periods (rrr =3.26, 95% CI [1.32, 8.04]) than their counter parts. Women with a history of pelvic or back pain were 2.7 times more likely to be suspicious for cancer (rrr =2.79, 95% CI [1.18, 6.58]) [Table 4] than their counter parts.

Table.4. Bivariate and Multivariable analysis of independent variables with pre-cancerous cervical lesion status among reproductive age women in Tigray, (n=883).

**Variables**			**VIA results**					
		**Negative**	**Positive**			**Suspicious**		
		N(%)	***N(%)***	***cRRR [95% CI]***	***aRRR [95% C.I.)***	***N(%)***	***cRRR [95% CI]***	***aRRR [95% C.I.)***
Age	15-30	257(86.5)	30(10.1)	1	1	14(3.6)	1	1
	31-45	416(87.6)	44(9.3)	0.91 (0.56, 1.48)	0.50(0.17, 1.45)	21(4.2)	0.93(0.410, 2.09)	2.34(0.74, 7.36)
	>=46	96(86.5)	5(4.5)	0.45(0.17, 1.18)	0.91(0.53, 1.55)	10(3.4)	2.68(1.08, 6.63) *	0.58(0.22, 1.55)
Marital status	Single	208(92.0)	14(6.2)	1	1	4(1.8)	1	1
	Married	472(88.7)	42(7.9)	1.32(0.71, 2.47)	0.94(0.46, 1.91)	18(3.4)	1.98(0.66, 5.93)	2.15(0.51, 8.97)
	Divorced/widowed	89(71.2)	23(18.4	3.84(1.89, 7.80)***	2.50(1.13, 5.52)*	13(10.4	7.59(2.41, 23.93)***	4.69(1.00, 21.84)*
Educational status	No formal education	240(83.9)	25(8.7)	1	1	21(7.3)	1	1
	Primary Education	325(89.0)	30(8.2)	0.89(0.51, 1.55)	0.78(0.41, 1.48)	10(2.7)	0.35(0.17, 0 .76) ***	0.32(0.11, 0.91)*
	Secondary Education	116(85.3)	17(12.5)	1.41(0.73, 2.71)	1.15(0.54, 2.43)	3(2.2)	0.29(0.09, 1.01)	0.25(0.05, 1.09)
	Tertiary Education	88(91.7)	7(7.3)	0.76(0.32, 1.83)	0.84(0.29, 2.39)	1(1.0)	0.13(0.02, 0.98)*	0.12(0.01, 1.41)
Occupation	Civil Servant	209(93.3)	11(4.9)	0.45(0.22, 0.92)*	0.78(0.41, 1.48)	4(1.8)	0.78(0.22, 2.82)	2.67(0.48, 14.68)
	Farmer	141(81.5)	21(12.1)	1.26(0.69, 2.30)	1.15(0.54, 2.43)	11(6.4)	3.21(1.16, 8.87)*	4.83(1.44, 16.13)*
	Merchant	172(84.3)	18(8.8)	0.89(0.47, 1.65)	0.84(0.29, 2.39)	14(6.9)	3.35(1.26, 8.89)*	4.85(1.52, 15.46)**
	No formal work	247(87.6)	29(10.3)	1	1	6(2.1)	1	1
Parity	0-4 children	628(87.6)	67(9.3)	1	1	22(3.1)	1	
	5-12 children	141(84.9)	12(7.2)	0.79(0.42, 1.51)	0.85(0.40, 1.79)	13(7.8)	2.63(1.29, 5.35)**	1.62(0.61, 4.25)
History of STI	Yes	156(78.4)	32(16.1)	2.67(1.65, 4.33)***	1.91(1.11, 3.27)*	11(5.5)	1.80(0.86, 3.75)	0.99(0.40, 2.42)
	No	613(89.6)	47(6.9)	1	1	24(3.5)	1	1
Blood between period	Yes	149(81.4)	16(8.7)	1.05(0.59, 1.88)	0.71(0.35, 1.45)	18(9.8)	4.40(2.21, 8.75)***	3.26(1.32, 8.04)*
	No	620(88.6)	63(9.0)	1	1	17(2.4)	1	1
Heavy menstruati[illegible]	Yes	70(74.5)	12(12.8)	1.78(0.92, 3.46)	1.23(0.55, 2.74)	12(12.8)	5.20(2.48, 10.9)***	2.45(0.92, 6.52)
	No	699(88.6)	67(8.5)	1	1	23(2.9)	1	1
Bleeding after [illegible]	Yes	78(77.2)	14(13.9)	1.90 (1.02, 3.55)*	1.25(0.57, 2.71)	9(8.9)	3.06 (1.38, 6.77) **	1.27(0.40, 3.99)
	No	691(88.4)	65(8.3)	1	1	26(3.3)	1	1
Contact bleeding	Yes	143(81.2)	19(10.8)	1.38(0.80, 2.39)	0.85(.42, 1.69)	14(8.0)	2.91(1.44, 5.87)**	0.81(0.30, 2.19)
	No	626(88.5)	60(8.5)	1	1	21(3.0)	1	1
Pain during sex	Yes	120(78.9)	23(15.1)	2.22(1.31, 3.74)**	1.39(0.70, 2.74)	9(5.9)	1.87(0.85, 4.09)	0.69(0.23, 2.01)
	No	649(88.8)	56(7.7)	1	1	26(3.6)	1	1
Pelvic or back pain	Yes	213(79.8)	33(12.4)	1.87(1.16, 3.00)*	1.60(0.90, 2.85)	21(7.9)	3.91(1.95, 7.84)***	2.79(1.18, 6.58)*
	No	556(90.3)	46(7.5)	1	1	14(2.3)	1	1
Unusual vagina [illegible]	Yes	239(79.9)	37(12.4)	1.95(1.22, 3.11)**	1.39(0.78, 2.49)	23(7.7)	4.26 (2.08, 8.68)***	2.22(0.91, 5.38)
	No	530(90.8)	42(7.2)	1	1	12(2.1)	1	1

NB: *P - value < 0.001 = ***, 0.001 - 0. 009 = ** and 0. 010 - 0. 05 = * and cRRR – crude relative risk ratio, aRRR - Adjusted relative risk ratio, C.I. - Confidence Interval*

Discussion

Ethiopia’s Federal Ministry of Health has prioritized cervical cancer prevention and treatment [23]; however, there is a scarcity of data related to the prevalence of pre-cancerous cervical lesions. This study explored the prevalence of pre-cancerous cervical lesions in Tigray, Northern Ethiopia.

Our study showed that 79 (8.9%) respondents were VIA-positive and 35 (4.0%) were suspicious for cervical cancer. The overall prevalence of VIA-positive was higher in this study than a study at Alameda Textile Factory in Tigray, which showed a VIA-positive rate of 6.7% [20]. A study at a Family Guidance Association (FGA) Clinic in Jimma, southern Ethiopia, showed that 12.9% of women were VIA-positive [19]. Studies in Swaziland, Cameroon, and Jakarta, Indonesia, showed VIA-positive rates of 15%, 3.33%, and 4.7%, respectively [10, 11, 12]. Our study also showed that 4.0% of women were suspicious for cancer compared to 1.2% of women in the study done in Jimma [19]. Though most are similar, the difference might be because of the skill difference of the care providers, and type acetic acid used for screening.

After adjusting for other variables, marital and educational status, occupation, history of STIs, bleeding between period, and pelvic or back pain were statistically significant risk factors for VIA-positive or suspicious for cancer.

Divorced or widowed women had a risk of being VIA-positive (p=0.023) and being suspicious (p=0.049) for cervical cancer than single women. A study done in Dar Es Salaam, Tanzania, showed thatdivorced women were more likely to be VIA positive [13], which is similar to this study. A study in Jakarta, Indonesia, revealed that the overall VIA positive rate was 4.7%; of which, 8.3% were married more than one timesin their life [12]. This implies that women who have started sexual practice and have divorced, widowed or separated and has no permanent

husband had a chance of exposing to multiple sexual partner; which increases the risk of exposure to HPV. Increasing outreach and screening intensity to divorced, separated, and widowed women could be a simple approach to streamlining the identification and treatment of women at risk for developing cervical cancer.

Women with primary education were approximately 70% less likely to be suspicious for cervical cancer (p=0.034) than women with no education, and education was not significantly correlated with VIA positivity. What's more, higher education levels, including secondary and tertiary, were not significantly associated with VIA-positive or suspicious. Similar to our study, a research findings from Dar Es Salaam, showed that women with low education had odds 4.3 higher of being VIA positive [13],. Studies in Gondar, Ethiopia, and India also showed that lack of awareness on cervical cancerr was a risk factor for cervical cancer [7,16]. This might be that increasing awareness including educational level could influence individuals to prevent themselves from any risk factors of cervical cancer.

In this study,women who are farmers and merchants in occupation had odds of 4.8 times higher of being suspicious for cervical cancer. Farmers may have less awareness of cervical cancer and lack of ability to participate in the early screening of cervical cancer. In the case of merchants, they could have the opportunity to visit different areas and stay out of houses. This may expose them to heightened risk due to increased chance of sexual practices that expose them to HPV.

Respondents with a history of STI had 1.91 times the risk of being VIA-positive (p=0.018), and though with history of STIs were not significantly associated with suspicious results. This was similar to studies done in Addis Ababa, Adama and Alamed Textile Factory, which revealted thatthe history of STI was a risk factor for pre-cancerous cervical lesion[17, 18, 20]. This could

be that women with STIs are more prone to aquire HPV infection, which is by far the dominant pathway for cervical cancer to initiate and progress.

Bleeding between period and pelvic or back pain were significantly associated with suspicious cance, which were 3.26 (p=0.010) and 2.79 (p=0.019) times higher risk respectively than their counter parts. Which was similar to a study done in Uganda revealed that the participants recognized inter-menstrual bleeding as a symptom of cervical cancer [9]. This might be mainly related to suspicious for cervical cancer;as the lesion advanced, the common sign and symptom are vaginal bleeding and pain.

In this study, 9.0% of 46 years or older women, were found suspicious for cervical cancer compared to approximately 9.5% for women less than 46 years of age. Age was not statistically significant.. Unlike this study, a study in India revealed that women 55 to 59 years of age had the highest risk of cervical cancer [8], this was also observed in one of the studies in Ethiopian[15]. This may implies that Ethiopian women may have earlier sexual initiation than Indian women that delaying introduction and progression of the disease to pre-cancerous cervical lesion.

In thisstudy, the presence of pre-cancerous cervical lesion'was not associated with current contraceptive use or age at first sexual contact. Unlike to this study, the age at first sexual contact was significant risk factor for pre-cancerous cervical lesion'in a study done in Jimma [19]. Unlike to this study, a research findings done in Jakarta, Addis Ababa and Adama, Uganda, and India showed that contraceptive use was a risk factor for pre-cancerous cervical lesions [9,8, 12,17,18]. Logically, long term contraceptive uses might be the risk factor for cervical cance. Hence, it might be because of that there are no more long time contraceptive users in the study area, which is evidenced by having high fertility rate. This might be preventive to pre-cancerous cervical lesion.

Strenngth and limitation of the study:

The primary data of pre-cancerous cervical lesion'was acertaned using VIA screening, rather than history taking. This method of data collection minimizes recall bias. However a case control study design could better estimate the association of independent variables with the outcome variable. This study did not include variables that have probability of being risk factors like HIV status and history of cervical cancer screening because of the absence of the services in some sites.

Conclusion

In this study, 8.9% and 4% of the women were positive and suspicious for pre-cancerous cervical lesion, respectively. The prevalence of pre-cancerous cervical lesion is high as compared to other regional prevalence in the country. Marital status, educational status, occupation, history of an STI, bleeding between period, and pelvic or back pain were risk factors for pre-cancerous cervical lesion. This also implies that cervical cancer prevention is better to be strengthened focusing on preventable risk factors, including exposure to sexually transmitted infections and specific vulnerable groups like divorced and widowed women. Further study of the age distribution of pre-cancerous cervical lesions is merited.

List of Abbreviations:

aRRR – Adjusted Relative Risk Ratio

cRRR - Crude Relative Risk Ratio

FMoH - Ethiopia’s federal ministry of health

HEWs - Health Extension Workers

HIC - High Income Countries

HIV - Human Immunodeficiency Virus

HPV - Human Papilloma Virus

LMICs - low-and-middle income countries

RRR - Relative Risk Ration

SCJ - Squamous-Columnar Junction

SD - Standard Deviation

STATA - STATistical Analysis

STI - Sexually Transmitted Infection

TRHB - Tigray Regional Health Bureau

VIA - Visual Inspection With Acetic acid

WDA - Women Development Army

Authors' contributions: GBA, SN, HH, and HGY Conceived, designed and developed this study as investigators. YB, GG, TA and GG: as senior advisors following, editing and recommending and providing constructive comments till finalization of this manuscript. All authors read and approved the final manuscript.

Data availability: All data generated and analyzed that are used to interpret the findings during this study are included in this manuscript. No multimedia data were collected.

Acknowledgments:

Our great appreciation goes to healthcare workers of the health facilities involved in the data collection and all study participants of the study. Moreover we would like to extend our acknowledgment for administrative and community health care workers for their support during data collection and facilitation of the project.

References

1. A report from WHO. A demonstration project, Prevention of cervical cancer through screening using visual inspection with acetic acid (VIA) and treatment with Cryotherapy.African population and health research center, 2012.
2. American cancer society report, a guideline for cervical cancer and its managements. www.cancer.org Last Medical Review: 4/11/2013 and Last Revised: 1/31/2014.
3. The Alliance for Cervical Cancer Prevention (ACCP), inspection with acetic acid (VIA): Evidence to date. www.alliance-cxca.org.
4. Data derived from official reports by WHO, United Nations, Cervical Cancer Global Crisis Card.The World Bank, IARC Globocan. http://www.who.int/hpvcentre/statistics/en/.
5. Campbell C. Assessment of Cervical Cancer Incidence, Histopathology, and Screening Practices Among Hispanic Women in Latin America and Michigan. A dissertation for the degree of Doctor of Philosophy, 2011.
6. Zohre Momenimovahed and Hamid Salehiniya, Incidence, mortality and risk factors of cervical cancer in the world. Biomedical Research and Therapy, BioMedPress, 2018, http://www.bmrat.org/index.php/BMRAT/article/view/386.
7. Rose I Anorlu, Cervical cancer: the sub-Saharan African perspective. Reproductive Health Matters, 2008;16(32).
8. Aswathy Sreedevi, Reshma Javed, and Avani Dinesh, Epidemiology of cervical cancer with special focus on India. Int J Womens Health. 2015; 7: 405–414, doi: 10.2147/IJWH.S50001.
9. Amos D. Mwaka, Christopher G. Orach, Edward M. Were, Georgios Lyratzopoulos, Henry Wabinga, Martin Roland, Awareness of cervical cancer risk factors and symptoms: cross-sectional community survey in post-conflict northern Uganda. Health Expect. 2016 Aug; 19(4): 854–867, doi: 10.1111/hex.12382.
10. Ginindza TG, Almonte M, Dlamini X, Sartorius B. Distribution of cervical abnormalities detected by visual inspection with acetic acid in Swaziland, 2011-2014: A retrospective study. African J Prim Heal care Fam Med. 2018;10(1):e1-e7, doi:10.4102/phcfm.v10i1.1773.

11. Nkfusai NC, Mubah TM, Yankam BM, Tambe TA, Cumber SN. Prevalence of precancerous cervical lesions in women attending Mezam Polyclinic Bamenda, Cameroon. Pan Afr Med J. 2019; 32:174. doi:10.11604/pamj.2019.32.174.16895.
12. Anggraeni TD, Peters AAW. Prevalence , Age Distribution , and Risk Factors of Visual Inspection With Acetic Acid-Positive From 2007 to 2011 in Jakarta. 2017;22(2):103-107.
13. Kahesa C, Kjaer SK, Ngoma T, et al. Risk factors for VIA positivity and determinants of screening attendances in Dar es Salaam , Tanzania. BMC Public Health. 2012; 12(1):1. doi:10.1186/1471-2458-12-1055.
14. Haile T. Beyene M. Berg A. prevention of cervical cancer in Ethiopia, a report from Women´s Health Center, Addis Ababa, Ethiopia and ESOG, Ethiopia, a model clinic in Addis Ababa, 2018.
15. Dye D., Solomon B., Claire H., et al, Complex care systems in developing countries. Breast Cancer Patient Navigation in Ethiopia,2009, 116:577–85.).
16. Birhanu Z. et al, Health seeking behavior for cervical cancer in Ethiopia: a qualitative study, International Journal for Equity in Health, BioMed center, 2012, 11:83.
17. Roza Teshome Kassa, Risk factors associated with precancerous cervical lesion among women screened at Marie Stops Ethiopia, Adama town, Ethiopia: a case control study, BMC Res Notes,2018, 11:145, https://doi.org/10.1186/s13104-018-3244-6.
18. Teame H, Addissie A, Ayele W, Hirpa S, Gebremariam A, Gebreheat G, et al. Factors associated with cervical precancerous lesions among women screened for cervical cancer in Addis Ababa, Ethiopia: A case control studyPLoS ONE, , 2018. 13(1): e0191506. https://doi.org/10.1371/journal.pone.0191506.
19. Deksissa ZM, Tesfamichael FA, Ferede HA. Prevalence and factors associated with VIA positive result among clients screened at Family Guidance Association of Ethiopia, south west area office, Jimma model clinic, Jimma, Ethiopia: A cross-sectional study, Womens Health. BMC Res Notes. 2015; 8(1):8-13. doi:10.1186/s13104-015-1594.
20. Kebede Haile Misgina, Hailay Seyoum Belay and Teklehaymanot Huluf Abraha, Prevalence of precancerous cervical lesion and associated factors among women in North Ethiopia, Journal of Public Health and Epidemiology, 2017, Vol. 9(3), pp. 46-50, DOI: 10.5897/JPHE2016.0891

21. Bayu H, Berhe Y, Mulat A, Alemu A Cervical Cancer Screening Service Uptake and Associated Factors among Age Eligible Women in Mekelle Zone, Northern Ethiopia,: A Community Based Study Using Health Belief Model. PLoS ONE, 2016, 11(3): e0149908. doi:10.1371/journal.pone.0149908
22. Yeneabeba Tilahun, National Cervical Cancer Prevention Training Package Participant Manual, Federal Ministry of Health Ethiopia, 2015.
23. Federal Democratic Republic of Ethiopia Ministry of Health. Guideline for Cervical Cancer Prevention and Control in Ethiopia.; 2015. https://www.iccp-portal.org/system/files/plans/Guideline Eth Final.pdf.
24. Seyoum T, Yesuf A, Kejela G, Gebremeskel F (2017) Utilization of Cervical Cancer Screening and Associated Factors among Female Health Workers in Governmental Health Institution of Arba Minch Town and Zuria District, Gamo Gofa Zone, Arba Minch, Ethiopia, 2016. Arch Cancer Res. Vol.5 No.4:165
25. Cecilia Chinemerem Nwabichie, Rosliza Abdul Manaf*, Suriani Binti Ismail, Factors Affecting Uptake of Cervical Cancer Screening Among African Women in Klang Valley, Malaysia, Asian Pac J Cancer Prev, 19 (3), 825-831

Click here to access/download
Supporting Information
Dataset Used for Analysis.xlsx

Epidemiology of ~~Cervical~~ Pre-cancerous Cervical Lesion and Risk Factors ~~Among~~ among Adult Women ~~I~~in Tigray, Ethiopia

Authors:

Gerezgiher B. Abera[1*], Henock G. Yebyo[2], Haftamu Hailekiros[3], Selam Niguse[3], Yibrah Berhe[3], Goitom Gigar[4], Tsehaye Asmelash[5], Gelila Goba[6]

Affiliation:

[1]School of Nursing, College of Health Sciences, Mekelle University, Mekelle, Ethiopia;

[2]School of Public Health, College of Health Sciences, Mekelle University, Mekelle, Ethiopia;

[3] School of Medicine, College of Health Sciences, Mekelle University, Mekelle, Ethiopia;

[4] Tigray Regional Health Bureau, Mekelle, Ethiopia;

[5]College of Health Sciences, Aksum University, Axum", Ethiopia;

[6]Department of Obstetrics and Gynecology, University of Illinois at Chicago, Chicago, USA

***Corresponding Author**: E-mail: gbamsc2002@gmail.com

ORCID ID https://orcid.org/0000-0003-4968-2885

Abstract

Background: Cervical cancer is a preventable disease if treated early, but remains the second leading cause of cancer-related mortality among women in low- and middle-income countries. Data on epidemiology and risk factors in these settings are scarce. This study aimed to assess the prevalence of precancerous lesions and risk factors in Tigray region, Ethiopia.

Methods: ~~A~~ This was a community-based, cross-~~-~~sectional study conducted among 900 women in Tigray region, Ethiopia, from March 2016 to June 2017.~~was used and 900 participants were recruited using multistage sampling and 883 were ressponeded correctly.~~ Data were collected using an interviewer administered questionnaire and screening with visual inspection with ascetic acid. Multinomial logistic regression analysis was conducted to estimate ~~predictors~~risk factors.

Results: Of the participants, 883 were responded correctly. Seventy-nine (8.95%) women were positive for pre-cancerous cervical lesion’~~pre-cancer lesion~~ and 35 (3.96%) were suspicious for cervical cancer. In the multivariable analysis, ~~D~~divorced or widowed women ~~had~~ had ~~2.5 and 4.7 times more risk~~ higher risk of ~~to be~~being positive for pre-cancerous cervical lesion ~~,~~’and or suspicious for cancer~~respectively, compared to~~than single women ~~(rrr=2.5, 95% CI [1.13, 5.52]); (rrr=4.69, 95% CI [1.00, 21.84])~~. The risk of having a suspicious for cancer result was ~~68%~~ lower for women with primary education ~~compared~~ than ~~to~~ those with no formal education ~~(rrr=0.32, 95% CI [1.00, 21.84])~~. ~~H~~Participants who had history of sexually transmitted infection ~~was~~ were ~~associated~~ at higher ~~with~~ of being positive for pre-cancerous cervical lesion’ result ~~pre-cancer lesion (rrr=1.91, 95% CI [1.11, 3.27]) whereas,~~. Participants who were ~~being~~ farmer ~~(rrr=4.83, 95% CI [1.44, 16.13]),~~ and merchant ~~(rrr=4.85, 95% CI [1.52, 15.46])~~in occupation, those who had bleeding between periods ~~(rrr =3.26, 95% CI [1.32, 8.04])~~ and had pelvic or back

Formatted: Heading 1, Line spacing: single, Adjust space between Latin and Asian text, Adjust space between Asian text and numbers

Formatted: Font: Times New Roman, 12 pt

Formatted: Font: Times New Roman, 12 pt

Formatted: Font: Times New Roman, 12 pt

Formatted: Font: Times New Roman, 12 pt

Formatted: Font: Times New Roman, 12 pt

pain ~~(rrr =2.79, 95% CI [1.18, 6.58])~~ were ~~associated with~~ at risk of having suspicious for cervical cancer results.

Conclusion: About 8.9% and 4% of the women were positive for pre-cancerous cervical lesion’and suspicious for ~~pre-cancer cervical lesion~~cancer, respectively. Marital status, education, sexually transmitted infection, bleeding, and pelvic pain were ~~predictors~~ risk factors of pre-cancerous cervical lesion’~~cervical precancerous lesions~~.

Key words: Epidemiology, Cervical, Pre-cancerous lesions.

Introduction

Cervical cancer ~~(CC)~~ is a public health problem worldwide [1], primarily in low and middle-income countries, though data are scarce [2, 3]. The disease is fatal, but preventable if treated early [4]. The World Health Organization (WHO) indicates that ~~CC~~ cervical cancer kills an estimated 275,000 women every year and 500,000 new cases reported worldwide [1]. Cervical cancer ~~CC~~ is the second largest killer among cancer-related death of women in low-and-middle income countries (LMICs) [4]. Reports from different countries showed that the incidence of ~~CC~~ cervical cancer is high in developing countries and sexually transmitted infections (STIs), hormonal influences, genetics and participant factors (risk taking behavior, substance abuse, alcohol use) are risk factors for cervical cancer ~~CC~~ [5, 6]. In Sub-Saharan Africa, an estimated 57,000 cases of cervical cancer ~~CC~~ occurred in 2000, comprising 22.2% of all female cancers [7].

A study from India revealed that late-stages reporting of disease was common with a peak age of cervical cancer ~~CC~~ incidence from 55 to 59 years and early marriage, multiple sexual partners, multiple pregnancies, oral contraceptives, and lack of awareness were significant risk factors [8].

Formatted: Font: Times New Roman, 12 pt

Formatted: Font: Times New Roman, 12 pt

Formatted: Heading 1, Line spacing: single

Formatted: Font: Times New Roman, 12 pt

A study in Uganda showed that 99% of women had heard about cervical cancer ~~CC~~ and 63% believed that family planning was a cause of cervical cancer ~~CC~~ as well as 85% recognizing inter-menstrual bleeding as a symptom of cervical cancer ~~CC~~ [9]. Other studies have also revealed the burden and public health importance of cervical cancer ~~CC~~ in LMICs [10-13].

In Ethiopia, cervical cancer is the most common cause of cancer death among female cancers with an overall mortality of 70% [14]. A study in Ethiopia showed that the mean age at diagnosis with cervical cancer was 48 years [15]. In a community based cross-sectional survey conducted in Gondar, northwest Ethiopia (2010), only 31% of respondents were knowledgeable about risk factors, symptoms, treatment options, prevention and early detection measures for cervical cancer [16]. Studies from Addis Ababa, Adama and Jimma showed that women 40 - 49 years of age, having a history of STIs, having two or more lifetime sexual partners and contraceptive use were risk factors of the cervical pre-cancerous lesion [17- 19]. Another institution based studies in Tigray revealed a prevalence of pre-cancerous cervical lesion of 6.7% and that STIs are predictors of the pre-cancerous cervical lesions [20, 21].

The Ethiopian Ministry of Health officially launched guideline for cervical cancer prevention and control in 2005, describes a “See and Treat approach” using visual inspection with acetic acid (VIA) screening and cryotherapy treatment for the reason of feasibility and affordability (22).

Although Ethiopia’s federal ministry of health (FMoH) has given priority for ~~CC~~ cervical cancer prevention and control, there is a scarcity of data related to pre-cancerous cervical lesions. Existing guidelines call for community based pre-cancerous cervical lesions epidemiology and risk factors [22]. Accordingly, this community-based research determines the prevalence and factors associated with pre-cancerous cervical lesions among adult women in Tigray, Ethiopia.

Materials and Methods ~~Method~~

Formatted: Heading 1 Char, Font: Calibri, 11 pt, Not Bold

Study setting and design

Formatted: Heading 2, Line spacing: single, Adjust space between Latin and Asian text, Adjust space between Asian text and numbers

We conducted a community-based, cross-sectional study in Tigray, Northern Ethiopia. Data were collected from March 2016 to June 2017. The study was conducted in Tigray region, which has seven zones, 34 rural and 18 urban districts ('Woredas' in local name) and 763 sub-districts ('Kebele/Tabia' in local name). In 2017, the Tigray Region had one specialized hospital, 14 general hospitals, 22 primary hospitals, 214 health centers, and 712 health posts as well as more than 500 private health facilities.

Study Population

Formatted: Heading 2, Left, Line spacing: single, Adjust space between Latin and Asian text, Adjust space between Asian text and numbers

Study participants: All adult women living in Tigray region for about six months or more were considered as a source population, and those who were voluntary and selected as study subjects were the study population.

Eligibility criteria*:* All adult women, who were sexually active and voluntarily consented to participate were included in the study. Those with history of hysterectomy were excluded from the study.

Formatted: Heading 2 Char, Font: Calibri, 11 pt, Not Bold, Not Italic

Sample size and sampling technique:

Formatted: Heading 2, Line spacing: single

The sample size was calculated with the aim of estimating a precise population prevalence of pre-cancerous cervical lesions in the Tigray Region. As there is no baseline study of prevalence of pre-cancerious cervical lesion, we assumed a 0.5 probability of the disease, 0.05 probability of alpha testing, 80% power and design effect of 2 accounting for two-stage sampling. Previous studies, the response rate ranges from 77.1% to 98.2%, which implies a 1.8% to 2.9.9% contingency(24, 25). We added 10% to the calculated sample size to account for non-responses, yielding a sample size of 846, which we rounded to 900 for convenience.

A multistage sampling was used to select the study subjects. Considering 40% of the 52 districts in the region, 21 of the districts which were distributed proportionally to zones were selected randomly. Since there are 315 sub-districts in the selected districts, 20% of the sub-districts (a total of 63 sub-districts) were selected randomly. Study participants were allocated proportionally to sub-districts based on the number of participants fulfilling the eligibility criteria in each sites. This was taken from the health extension workers registration book.

Recruitment process: A large mobilization was preceded the screening. This effort entailed training of Health Extension Workers (HEWs) from selected sub-districts. HEWs in turn oriented women in their communities. They also mobilized Women Development Army (WDA) leaders and members as well as sub-district leaders by providing brief orientations about cervical cancer ~~(CC)~~ as well as upcoming screening dates and locations. Grassroots mobilization was

complimented by promotion on regional radio stations by the Tigray Regional Health Bureau (TRHB).

Following community mobilization, participants were invited for screening to a total of 17 primary and general hospitals in the sub districts which have cervical cancer screening cencers using visual inspection with acetic acid (VIA). As per the estimated number of expected participants, a key value of 4 was calculated. Every forth women arriving at the hospitals following grassroots mobilization were selected and invited to participate in the study. Finally, those who fulfill the eligibility criteria were recruited as a study subject.

Data collection procedure:

Formatted: Heading 2, Line spacing: single, Adjust space between Latin and Asian text, Adjust space between Asian text and numbers

Women providing consent were interviewed using an interviewer administered questionnaire to obtain socio-demographic and risk-factors information, including health related behaviors and lifestyle, knowledge, attitude, and clinical attributes.

Clinical testing was conducted by certified nurses trained by the TRHB according to the national cervical cancer prevention guidelines [23]. A ~~Visual Inspection with Acetic Acid (~~VIA~~)~~ screening protocol was developed based on the national guideline, The protocol included private exam area, examination table, trained health professionals, adequate light source, sterile vaginal speculum, new examination or surgical gloves, large cotton swabs, diluted (3-5%) acetic acid, small bowl, containers with 0.5% chlorine solution, plastic bucket with a plastic bag and quality assurance system to maximize accuracy.

Pre-cancerous cervical lesions was determined by vaginal speculum exam during which provider applied diluted acetic acid to the cervix. Visual confirmation of color change by the naked eye was used to determine the presence or absence of lesions. The test was considered positive if a sharp, distinct, well-defined, dense (opaque/dull or oyster) Aceto-white area was present with

raised margins touching the Squamo-columnar junction (SCJ). The test was considered negative if no or faint Aceto-white lesions and/or smooth surface was present. The test was cinsudered suspicious for CC cervical cancer where clinically visible ulcerative, cauliflower-like growth or ulcer, oozing, and/or bleeding on touch were observed.

The history of sexually transmited infection (STI) was determined if any one of the sign and symptoms (discharge, offensive secretion, itching, dysuria, lower abdominal pain, and fever) were present. The variable 'history of cervical cancer screening' was not included in our study, since most of our sites did not have the screening modality previously and we may get no participants with previous history of cervical cancer screening.

Data analysis

Data was were analyzed using statistical analysis (STATA) version14. Data entry and clearance was done using descriptive analysis to check if any missing and inappropriate categories. Descriptive statistics were presented in texts and tables. The dependent variable was pre-cancerous cervical lesion'cervical pre-cancer status, in which the screening result had three possible outcomes: Negative, Positive and Suspicious for VIA. A multinomial regression model was used to check the influence of socio-demographic, risk factors and reproductive health characteristics on CC pre-cancerous cervical lesion 'statuscervical cancer screening outcomes. Unadjusted multinomial regression was used to examine the separate bivariate relationship between each independent variables and the dependent variable. The variables with p-value < 0.05 in the unadjusted multinomial regression was considered as significant and used in the multivariable multinomial logistic regression. Relative risk ratios (rrr) were used to assess the multivariate association of the predictor variables with the dependent variable.

Formatted: Font: Times New Roman, 12 pt

Formatted: Heading 2, Line spacing: single

Formatted: Font: Times New Roman, 12 pt

Formatted: Font: Times New Roman, 12 pt

Formatted: Justified

Ethical consideration: Before beginning data collection, ethical clearance was obtained from M~~m~~ekelle U~~u~~niversity, ~~collage~~ Collage of ~~health~~ Health ~~sience~~ Siences ~~research~~ Research and ~~community~~ Community ~~service~~ Service ~~commiyyee~~ Committee (Registration number – ERC 0597/2015) and support letter was obtained from Tigray regional health bureau. Participants were oriented the general objective of the study, procedure of data collection, privacy and confidenciality of the information obtained, it was also informed that participation is voluntary based and the possibility to withdraw at any time when they need. Finally written informed consent was obtained from the participants prior to the actual data collection.

Formatted: Heading 2 Char, Font: Calibri, 11 pt, Not Bold

Formatted: Heading 1, Line spacing: single

Results

Socio-demographic c~~C~~haracteristics of the study participants

A total of 883 questionnaires was collected with complete information, equating to a response rate of 98.1%. More than half of the participants 496 (56.2%) came from urban districts. Mean age of participants was 35.69 years (standard deviation (SD) ±8.45, range 16 - 62). Among all respondents, 532 (60.2%) were married, 723 (81.9%) were Orthodox Christian, 286 (32.4%) had no formal education, 282 (31.9%) had no formal work, and 717 (81.2%) had zero to four children. Median income was Ethiopian Birr 1,500 and 515 (58.3%) women earned between Ethiopian Birr 0 and 2,000 per month [Table 1].

Table 1. Distribution of Socio-demographic characteristics by pre-cancerous cervical lesion status ~~VIA results~~ among women of reproductive age in Tigray, (n=883)

Formatted: Font: 12 pt

Formatted: Font: 12 pt

Socio-demographic Variables	Groups	VIA results			
		**Negative** N (%)	**Positive** N (%)	**Suspicious** N (%)	**Total** N (%)
Residence	Rural	345(89.1)	28(7.2)	14(3.6)	387(43.8)
	Urban	424(85.5)	51(10.3)	21(4.2)	496(56.2)
Age (yr)	15-30	257(86.5)	30(10.1)	10(3.4)	297 (33.6)
	31-45	416(87.6)	44(9.3)	15(3.2)	475 (53.8)
	>=46	96(86.5)	5(4.5)	10(9.0)	111 (12.6)
Marital status	Single	208(92.0)	14(6.2)	4(1.8)	226(25.6)
	Married	472(88.7)	42(7.9)	18(3.4)	532(60.2)
	Divorced/Others	89(71.2)	23(18.4)	13(10.4)	125(14.2)
Religion	Orthodox	627(86.7)	64(8.9)	32(4.4)	723(81.9)
	Muslim and others	142(88.8)	15(9.4)	3(1.9)	160(18.1)
Educational status	No formal education	240(83.9)	25(8.7)	21(7.3)	286(32.4)
	Primary education	325(89.0)	30(8.2)	10(2.7)	365(41.3)
	Secondary education	116(85.3)	17(12.5)	3(2.2)	136(15.4)
	Tertiary education	88(91.7)	7(7.3)	1(1.0)	96(10.9)
Occupation	Civil servant	209(93.3)	11(4.9)	4(1.8)	224(25.4)
	Farmer	141(81.5)	21(12.1)	11(6.4)	173(19.6)
	Merchant	172(84.3)	18(8.8)	14(6.9)	204(23.1)
	No formal work	247(87.6)	29(10.3)	6(2.1)	282(31.9)
Monthly income	0-2000	439(85.2)	51(9.9)	25(4.9)	515(58.3)
	2001-4000	158(86.3)	16(8.7)	9(4.9)	183(20.7)
	4001-6000	105(92.1)	8(7.0)	1(0.9)	114(12.9)
	6001-8000	16(88.9)	2(11.1)	0(0.0)	18(2.0)
	8001-10000	51(96.2)	2(3.8)	0(0.0)	53(6.0)
Parity	0-4 children	628(87.6)	67(9.3)	22(3.1)	717(81.2)

	5-12 children	141(84.9)	12(7.2)	13(7.8)	166(18.79)

Risk factors of Cervical Cancer ’~~Cervical precancerous lesion~~:

A total of 782 (88.6%) had visible SCJ. Among all the participants, 79 (8.9%) were positive for VIA, 35 (4.0%) were suspicious for cancer, and 769 (87.1%) were negative for pre-cancer lesions. A total of 776 (87.9%) reported that their age at first sexual contact was less than 20 years. Of the total, 344 (39.0%) respondents had more than two sexual partners in their lifetime and 199 (22.5%) experienced STIs. Thirty-two (3.6%) were smokers and one-third of respondents consumed alcohol 255 (28.9%). Corticosteroids were used in 97 (11.0%) women and 199 (22.5%) where current contraceptive users. Seventy (7.9%) had a family history of CC. Out of these women, nine (12.9%) were VIA-positive [Table 2].

Table. 2. Distribution of risk factors by pre-cancerous cervical lesion status ~~VIA results~~ among women of reproductive age in Tigray, (n=883).

Risk factors of cervical cancer		VIA results Negative N (%)	Positive N (%)	Suspicious N (%)	Total N (%)
Age at first sexual contact	≤ 20 years age	675(87.0)	66(8.5)	35(4.5)	776(87.9)
	> 20 years age	94(87.9)	13(12.1)	0(0.0)	107(12.1)
Number Sexual Partner	Only one	461(88.3)	45(8.6)	16(3.1)	522(59.1)
	Two or more	293(85.2)	33(9.6)	18(5.2)	344(39.0)
	No or unspecified	15(88.2)	1(5.9)	1(5.9)	17(1.9)
History of STI	Yes	156(78.4)	32(16.1)	11(5.5)	199(22.5)
	No	613(89.6)	47(6.9)	24(3.5)	684(77.5)
Active smoking	Yes	29(90.6)	2(6.2)	1(3.1)	32(3.6)
	No	740(87.0)	77(9.0)	34(4.0)	851(96.4)
Alcohol consumption	Yes	232(91.0)	18(7.1)	5(2.0)	255(28.9)
	No	537(85.5)	61(9.7)	30(4.8)	628(71.1)
Chronic corticosteroids use	Yes	86(88.7)	8(8.2)	3(3.1)	97(11.0)
	No	683(86.9)	71(9.0)	32(4.1)	786(89.0)
Current contraceptive use	Yes	169(84.9)	21(10.6)	9(4.5)	199(22.5)
	No	600(87.7)	58(8.5)	26(3.8)	684(77.5)
Family history of cervical cancer	Yes	60(85.7)	9(12.9)	1(1.4)	70(7.9)
	No	709(87.2)	70(8.6)	34(4.2)	813(92.1)

NB: *STI – sexually transmitted infection*

Formatted: Font: Times New Roman, 12 pt, Not Italic

Formatted: Font: 12 pt

Formatted: Font: 12 pt

Formatted: Justified, Space After: 6 pt, Line spacing: single

Formatted: Superscript

Formatted: Superscript

Signs and symptoms of Cervical Cancer:

From the study participants, 183 (20.7%) women experienced bleeding between menstruation, 94 (10.6%) experienced heavy menstruation, 176 (19.9%) experienced contact bleeding and 152 (17.2%) women experienced pain during sex. Pelvic or back pain was reported by 267 (30.2%) women [Table 3].

Table 3. Distribution of signs and symptoms by pre-cancerous cervical lesion status ~~VIA results~~ among women of reproductive age in Tigray, (n=883).

Sign and symptoms and cervical cancer status		VIA results			
		Negative N (%)	Positive N (%)	Suspicious N (%)	N (%)
Bleeding between menstruation	Yes	149(81.4)	16(8.7)	18(9.8)	183(20.7)
	No	620(88.6)	63(9.0)	17(2.4)	700(79.3)
Heavy menstruation	Yes	70(74.5)	12(12.8)	12(12.8)	94(10.6)
	No	699(88.6)	67(8.5)	23(2.9)	789(89.4)
Bleeding after menopause	Yes	78(77.2)	14(13.9)	9(8.9)	101(11.4)
	No	691(88.4)	65(8.3)	26(3.3)	782(88.6)
Contact bleeding	Yes	143(81.2)	19(10.8)	14(8.0)	176(19.9)
	No	626(88.5)	60(8.5)	21(3.0)	707(80.1)
Pain during sex	Yes	120(78.9)	23(15.1)	9(5.9)	152(17.2)
	No	649(88.8)	56(7.7)	26(3.6)	731(82.8)
Pelvic or back pain	Yes	213(79.8)	33(12.4)	21(7.9)	267(30.2)
	No	556(90.3)	46(7.5)	14(2.3)	616(69.8)
Unusual vaginal discharge	Yes	239(79.9)	37(12.4)	23(7.7)	299(33.9)
	No	530(90.8)	42(7.2)	12(2.1)	584(66.1)

Factors associated with pre-cancerous Cervical Lession:~~cervical screening VIA result~~:

In the multinomial analysis "Negtive result of the dependent variable was used as a base reference against the positive and suspicious results; hence the relative risk is relative to those who have negative results.

Multinomial bivariate analysis revealed that age, marital and education status, occupation, symptoms of ~~CC~~cervical cancer, parity, STI history of partner, and lower abdominal pain were significantly associated with positive or suspicious results (p-value < 0.05). Other variables were not significantly associated with pre-cancerous cervical lesion'~~cervical precancerous lesions~~. The

Formatted: Font: Bold, English (United States)

Formatted: Font: 12 pt

Formatted: Font: 12 pt

Formatted: Heading 2, Line spacing: single

Formatted: Font: Times New Roman, 12 pt

multivariate logistic regression analysis showed that marital and educational status, occupation, history of STIs, bleeding between menstruation, and pelvic or back pain were significantly associated with the presence of pre-cancerous cervical lesion’~~cervical precancerous lesions~~. Divorced or widowed women ~~had~~ were 2.5 and 4.7 times ~~risk~~more likely, to be VIA-positive and suspicious respectively ~~compared~~ than ~~to~~ single women (rrr = 2.50, 95% confidence interval (CI) [1.13, 5.52]); (rrr=4.69, 95% CI [1.00, 21.84]). The risk of being suspicious was 68% lower for women with primary education ~~compared~~ than ~~to~~ those with no formal education (rrr=0.32, 95% CI [1.00, 21.84]). The risk of being suspicious for cancer was 4.8 times higher among farmers (rrr=4.83, 95% CI [1.44, 16.13]) and merchants (rrr=4.85, 95% CI [1.52, 15.46]) ~~compared to~~than respondents with no formal work.

The risk of being VIA-positive was 90% higher ~~among~~ for women with a history of STIs (rrr=1.91, 95% CI [1.11, 3.27]) than their counter parts. The risk of being suspicious for cancer was 3.26 times higher ~~among~~ for women who experienced bleeding between periods (rrr =3.26, 95% CI [1.32, 8.04]) than their counter parts. Women with a history of pelvic or back pain were 2.7 times more likely to be suspicious for cancer (rrr =2.79, 95% CI [1.18, 6.58]) [Table 4] than their counter parts.

Formatted: Font: Times New Roman, 12 pt

Formatted: Font: 14 pt

Table.4. Bivariate and Multivariable analysis of independent variables with pre-cancerous cervical lesion ~~cervical cancer~~ status among reproductive age women in Tigray, (n=883).

Variables		VIA results						
		Negative	Positive			Suspicious		
		N(%)	*N(%)*	*cRRR [95% CI]*	*aRRR [95% C.I.)*	*N(%)*	*~~CRRR~~ cRRR [95% CI]*	*~~ARRR~~ aRRR [95% C.I.)*
Age	15-30	257(86.5)	30(10.1)	1	~~0.91(0.53, 1.55)~~1	14(3.6)	1	~~0.58(0.22, 1.55)~~1
	31-45	416(87.6)	44(9.3)	0.91 (0.56, 1.48)	0.50(0.17, 1.45)	21(4.2)	0.93(0.410, 2.09)	2.34(0.74, 7.36)
	>=46	96(86.5)	5(4.5)	0.45(0.17, 1.18)	0.91(0.53, 1.55)	10(3.4)	2.68(1.08, 6.63) *	0.58(0.22, 1.55)
Marital status	Single	208(92.0)	14(6.2)	1	1	4(1.8)	1	1
	Married	472(88.7)	42(7.9)	1.32(0.71, 2.47)	0.94(0.46, 1.91)	18(3.4)	1.98(0.66, 5.93)	2.15(0.51, 8.97)
	Divorced/widowed	89(71.2)	23(18.4	3.84(1.89, 7.80)***	2.50(1.13, 5.52)*	13(10.4	7.59(2.41, 23.93)***	4.69(1.00, 21.84)*
Educational status	No formal education	240(83.9)	25(8.7)	1	1	21(7.3)	1	1
	Primary Education	325(89.0)	30(8.2)	0.89(0.51, 1.55)	0.78(0.41, 1.48)	10(2.7)	0.35(0.17, 0 .76) ***	0.32(0.11, 0.91)*
	Secondary Education	116(85.3)	17(12.5)	1.41(0.73, 2.71)	1.15(0.54, 2.43)	3(2.2)	0.29(0.09, 1.01)	0.25(0.05, 1.09)
	Tertiary Education	88(91.7)	7(7.3)	0.76(0.32, 1.83)	0.84(0.29, 2.39)	1(1.0)	0.13(0.02, 0.98)*	0.12(0.01, 1.41)
Occupation	Civil Servant	209(93.3)	11(4.9)	0.45(0.22, 0.92)*	0.78(0.41, 1.48)	4(1.8)	0.78(0.22, 2.82)	2.67(0.48, 14.68)
	Farmer	141(81.5)	21(12.1)	1.26(0.69, 2.30)	1.15(0.54, 2.43)	11(6.4)	3.21(1.16, 8.87)*	4.83(1.44, 16.13)*
	Merchant	172(84.3)	18(8.8)	0.89(0.47, 1.65)	0.84(0.29, 2.39)	14(6.9)	3.35(1.26, 8.89)*	4.85(1.52, 15.46)**
	No formal work	247(87.6)	29(10.3)	1	1	6(2.1)	1	1
Parity	0-4 children	628(87.6)	67(9.3)	1	1	22(3.1)	1	
	5-12 children	141(84.9)	12(7.2)	0.79(0.42, 1.51)	0.85(0.40, 1.79)	13(7.8)	2.63(1.29, 5.35)**	1.62(0.61, 4.25)
History of STI	Yes	156(78.4)	32(16.1)	2.67(1.65, 4.33)***	1.91(1.11, 3.27)*	11(5.5)	1.80(0.86, 3.75)	0.99(0.40, 2.42)
	No	613(89.6)	47(6.9)	1	1	24(3.5)	1	1
Blood between period	Yes	149(81.4)	16(8.7)	1.05(0.59, 1.88)	0.71(0.35, 1.45)	18(9.8)	4.40(2.21, 8.75)***	3.26(1.32, 8.04)*
	No	620(88.6)	63(9.0)	1	1	17(2.4)	1	1
Heavy menstruati	Yes	70(74.5)	12(12.8)	1.78(0.92, 3.46)	1.23(0.55, 2.74)	12(12.8)	5.20(2.48, 10.9)***	2.45(0.92, 6.52)
	No	699(88.6)	67(8.5)	1	1	23(2.9)	1	1
Bleeding after	Yes	78(77.2)	14(13.9)	1.90 (1.02, 3.55)*	1.25(0.57, 2.71)	9(8.9)	3.06 (1.38, 6.77) **	1.27(0.40, 3.99)
	No	691(88.4)	65(8.3)	1	1	26(3.3)	1	1
Contact bleeding	Yes	143(81.2)	19(10.8)	1.38(0.80, 2.39)	0.85(.42, 1.69)	14(8.0)	2.91(1.44, 5.87)**	0.81(0.30, 2.19)
	No	626(88.5)	60(8.5)	1	1	21(3.0)	1	1
Pain during sex	Yes	120(78.9)	23(15.1)	2.22(1.31, 3.74)**	1.39(0.70, 2.74)	9(5.9)	1.87(0.85, 4.09)	0.69(0.23, 2.01)
	No	649(88.8)	56(7.7)	1	1	26(3.6)	1	1
Pelvic or back pain	Yes	213(79.8)	33(12.4)	1.87(1.16, 3.00)*	1.60(0.90, 2.85)	21(7.9)	3.91(1.95, 7.84)***	2.79(1.18, 6.58)*
	No	556(90.3)	46(7.5)	1	1	14(2.3)	1	1
Unusual vagina	Yes	239(79.9)	37(12.4)	1.95(1.22, 3.11)**	1.39(0.78, 2.49)	23(7.7)	4.26 (2.08, 8.68)***	2.22(0.91, 5.38)
	No	530(90.8)	42(7.2)	1	1	12(2.1)	1	1

NB: *P - value < 0.001 = ***, 0.001 - 0. 009 = ** and 0. 010 - 0. 05 = * and cRRR – crude relative risk ratio, aRRR - Adjusted relative risk ratio, C.I. - Confidence Interval*

Formatted: Font: Times New Roman, 12 pt

Formatted: Superscript

Formatted: Space Before: 24 pt, Line spacing: Multiple 1.15 li

Discussion

Ethiopia’s Federal Ministry of Health has prioritized CC cervical cancer prevention and treatment [23]; however, there is a scarcity of data related to the prevalence of pre-cancerous cervical lesions and abnormal screening results. This study explored the prevalence of pre-cancerous cervical lesions and abnormal results in Tigray, Northern Ethiopia.

Our study showed that 79 (8.9%) respondents were VIA-positive and 35 (4.0%) were suspicious for CCcervical cancer. The overall prevalence of VIA-positive was higher in this study than a study at Alameda Textile Factory in Tigray, which showed a VIA-positive rate of 6.7% [20]. A study at a Family Guidance Association of Ethiopia (FGAE) Clinic in Jimma, southern Ethiopia, showed that 12.9% of women were VIA-positive [19]. Studies in Swaziland, Cameroon, and Jakarta, Indonesia, showed VIA-positive rates of 15%, 3.33%, and 4.7%, respectively [10, 11, 12]. Our study also showed that 4.0% of women were suspicious for cancer compared to 1.2% of women in the study done in Jimma [19]. Though most are similar, the difference might be because of the skill difference of the care providers, and type acetic acid used for screening.

After adjusting for other variables, marital and educational status, occupation, history of STIs, bleeding between period, and pelvic or back pain were statistically significant risk factors for VIA-positive or suspicious for cancer.

Divorced or widowed women had a risk of being VIA-positive (p=0.023) and being suspicious (p=0.049) for CC cervical cancer than single women. In a A study done in Dar Es Salaam, Tanzania, showed that divorced/separated women were more likely to be VIA -positive [13], which is similar to this study. A study iIn Jakarta, Indonesia, revealed that the overall VIA

Formatted: Font: Cambria, Font color: Custom Color(RGB(54,95,145))

positive rate was 4.7%; of which, 8.3% ~~of~~ ~~VIA-positive women~~ were married more than one times~~once compared to~~in their life ~~4.7% of overall women~~ [12]. This implies that ~~W~~women who have started sexual practice and have divorced, widowed or separated and has no permanent husband had a chance of exposing to multiple sexual partner; which increases the risk of exposure to HPV. Increasing outreach and screening intensity to divorced, separated, and widowed women could be a simple approach to streamlining the identification and treatment of women at risk for developing ~~CC~~cervical cancer.

Women ~~in our study~~ with primary education were approximately 70% less likely to be suspicious for ~~CC~~ cervical cancer (p=0.034) than women with no education, ~~though~~ and education was not significantly correlated with VIA~~-~~positivity~~e~~. What's more, higher education levels, including secondary and tertiary, were not significantly associated with VIA-positive or suspicious. ~~In~~ Similar to our study, a research findings from Dar Es Salaam, showed that women with low education had odds 4.3 higher of being VIA~~-~~positive [13],. Studies in Gondar, Ethiopia, and India also showed that lack of awareness ~~of~~ on ~~CC~~ cervical cancerr was a risk factor for ~~CC~~ cervical cancer [7,16]. This might be that i~~I~~ncreasing awareness including educational level could influence individuals to prevent themselves from any risk factors of cervical cancer.

In this study,w~~W~~omen who are farmers and merchants in occupation had odds of 4.8 times higher of being suspicious for ~~CC~~ cervical cancer~~in our study~~. Farmers may ~~Farmers may~~ have less awareness of ~~CC~~ cervical cancer and lack ~~the~~ of ability to ~~obtain~~ participate in the early screening of cervical cancer. In the case of merchants, ~~these women are~~they could have the opportunity to visit different areas and stay out of houses. ~~wealthier than farmers in the study~~This may expose them ~~area and may have~~to heightened risk due to ~~more~~increased chance of– sexual practices that expose them to HPV.

Respondents with a history of STI had 1.91 times the risk of being VIA-positive (p=0.018), and though with history of STIs were not ~~statistically~~ significantly associated with suspicious results. This was similar to studies done in Addis Ababa, Adama and ~~at the~~ Alamed Textile Factory, which revealted that~~where~~ the history of STI was a risk factor for pre-cancerous cervical lesion~~,~~ ~~cervical precancerous lesions~~ [17, 18, 20]. ~~One explanation for this is~~This could be that women with STIs are more prone to aquire HPV infection, which is by far the dominant pathway for ~~CC~~ cervical cancer to initiate and progress.

Bleeding between period and pelvic or back pain were significantly associated with suspicious ~~results~~cance, which were 3.26 (p=0.010) and 2.79 (p=0.019) times higher risk respectively than their counter parts. ~~A~~ Which was similar to a study ~~from~~ done in Uganda revealed that the participants recognized inter-menstrual bleeding as a symptom of ~~CC~~ cervical cancer [9]. ~~This is~~This might be mainly related to suspicious for cervical cancer~~; that~~ as the lesion advanced, the common sign and symptom are vaginal bleeding and pain.

~~Among~~ In this study, 9.0% of 46 years or older women ~~46 years of age or older~~, ~~9.0%~~ were found ~~to be~~ suspicious for ~~CC~~ cervical cancer compared to approximately 9.5% for women less than 46 years of age. ~~Though a~~Age was not statistically significant.~~, further inquiry is needed to confirm and explain these variations~~. Unlike this study,~~In~~ a study in India~~,~~ revealed that women 55 to 59 years of age had the highest risk of ~~CC~~ cervical cancer [8], this was also observed in one of the studies in Ethiopian ~~study~~ [15]. This may implies that Ethiopian women may have earlier sexual initiation than Indian women that delaying introduction and progression of the disease to pre-cancerous cervical lesion.

In thi~~s~~se ~~present~~ study, the presence of pre-cancerous cervical lesion~~,~~ ~~cervical precancerous lesion~~ was not associated with current contraceptive use or age at first sexual contact. Unlike to this

Formatted: Font: Times New Roman, 12 pt

Formatted: Font: Times New Roman, 12 pt

study, ~~T~~the age at first sexual contact was significant ~~predictor~~ risk factor for pre-cancerous cervical lesion~~cervical precancerous lesion~~ in ~~the~~ a study done in Jimma [19]. Unlike to this study, ~~A study in Jakarta showed that current use of hormonal contraceptives was associated with odds of VIA positive [12], whereas~~ a research findings ~~studies~~ done ~~in~~ in Jakarta, Addis Ababa and Adama ~~(Ethiopia)~~, Uganda, and India showed that contraceptive use was a risk-factor for pre-cancerous cervical lesions [9,8, 12,17,18]. Logically, long term contraceptive uses might be the risk factor for cervical cance. ~~In Ethiopia,~~Hence, it might be because of that ~~long time contraceptive users are few~~there are no more long time contraceptive users in the study area, which is evidenced by having high fertility rate. This ~~may~~ might ~~not predispose~~be preventive to pre-cancerous cervical lesion~~cervical precancerious lesion~~.

Formatted: Font: Times New Roman, 12 pt

Formatted: Font: Times New Roman, 12 pt

Strenngth and limitation of the study:

The primary data of pre-cancerous cervical lesion~~cervical precancerous lesion~~ was acertaned using VIA screening, rather than history taking. This method of data collection minimizes recall bias. However a case control study design could better estimate the association of independent variables with the outcome variable. This study did not include variables that have probability of being risk factors like HIV status and history of cervical cancer screening because of the absence of the services in some sites.

Formatted: Font: Times New Roman, 12 pt

Formatted: Font: 14 pt

Formatted: Heading 1, Line spacing: single, Adjust space between Latin and Asian text, Adjust space between Asian text and numbers

Conclusion

In this study, 8.9% and 4% of the women were positive and suspicious for pre-cancerous cervical lesion, respectively. The prevalence of pre-cancerous cervical lesion is high as compared to other regional prevalence in the country. Marital status, educational status, occupation, history of an STI, bleeding between period, and pelvic or back pain were risk factors for pre-cancerous cervical lesion. ~~Cervical~~ This also implies that cervical cancer prevention is better to be strengthened focusing on preventable risk factors, including exposure to sexually transmitted infections and specific vulnerable groups like divorced and widowed women. Further study of the age distribution of pre-cancerous cervical lesions ~~and suspicious results~~ is merited.

List of Abbreviations

~~ARRR~~ aRRR – Adjusted Relative Risk Ratio

~~cRRR~~ cRRR - Crude Relative Risk Ratio

FMoH - Ethiopia's federal ministry of health

HEWs - Health Extension Workers

HIC - High Income Countries

HIV - Human Immunodeficiency Virus

HPV - Human Papilloma Virus

LMICs - low-and-middle income countries ~~Low Income Countries~~

RRR - Relative Risk Ration

SD - Standard Deviation

STATA - STATistical Analysis

STI - Sexually Transmitted Infection

~~SD - Standard Deviation~~

SCJ - Squamous-Columnar Junction

TRHB - Tigray Regional Health Bureau

VIA - Visual Inspection With Acetic acid

WDA - Women Development Army

Declarations

Ethics Approval and Consent to Participate:

The study protocol was evaluated and approved by the Research Ethics Review Committee of College of Health Sciences, Mekelle University. Official cooperation letters were obtained from Mekelle University, Tigray Regional Health Bureau, and zonal health offices. Moreover, the purpose, advantage and disadvantage of the study were described to the study participants. The consent involves permission to disseminate the findings of the study through scientific workshop presentations and publications. The study participants were told that they have full right to discontinue the study. Confidentiality and any special data security requirements were maintained and assured. The participants were informed that results of the screening that have a direct benefit to their health would be informed to physicians working in the nearby health facility. Finally written informed consent was obtained from the participants prior to the actual data collection.

Consent for publications: Not applicable

Data availability: All the data sets used and/or analyzed during the current study are included in the manuscript. Data set has been uploaded with this revised documents.

Authors' contribution

GBA, HY, SN and HH involved during the conception and designing of the study. GBA, SN, HH carried out data collection and supervision of the data collection. GBA, HY, SN and HH performed statistical analysis. YB, GG1, AT and GG2 were participated in following, editing, recommending and providing constructive comments till finalization of this manuscript. All authors read and approved the final manuscript.

Authors' information:

GBA is an instructor and researcher as associate professors in maternity and reproductive health, at College of Health sciences, Mekelle

YB is a physician gynecologist and instructor as associate professors and researchers at College of Health sciences, Mekelle University;

AT is a microbiologist instructor, and Presidant of the University and PhD holder as associate professor and researcher at college of Health Sciences, Axum University;

SN and HH are a microbiologist instructors as assistant professors and researchers at College of health Sciences, Mekelle University;

HY is an instructor, assistant professor in biostatistics and researcher in Public Health, College of Health Sciences, Mekelle University. Currently living in Germen.

GG1 is a public health in professiona and currently deputy general director of FMHACA, Addis Ababa

GG2 is a Female physician GYNOBS and global women fellowship program director at University of Illinoise, Chicago.

Competing Interests: The authors have no competing interests to declare.

Funding: Tigray Regional Health Bureau and Mekelle University funded the data collection prosess. "The author(s) did not receive any funding for this paperwork or manuscript publication from any source." "The Mekelle University had no role in study design, data collection and analysis, decision to publish, or preparation of the manuscript."

ORCID iD https://orcid.org/0000-0003-4968-2885

Acknowledgments:

We aknowledge the Tigray Regional Health Bureau and Mekelle University for their support in the data collection process. Our great appreciation goes to healthcare workers of the health facilities involved in the data collection and all study participants of the study for their commitment and geniun participation. Moreover we would like to extend our acknowledgment for administrative and community health care workers for their support during data collection and facilitation of the project.

Formatted: Justified

References

1. A report from WHO. A demonstration project, Prevention of cervical cancer through screening using visual inspection with acetic acid (VIA) and treatment with ~~cryotherapy~~Cryotherapy.~~, ~~African population and health research center, 2012.
2. American cancer society report, a guideline for cervical cancer and its managements.~~,~~ www.cancer.org Last Medical Review: 4/11/2013 and Last Revised: 1/31/2014.
3. The Alliance for Cervical Cancer Prevention (ACCP), inspection with acetic acid (VIA): Evidence to date.~~,~~ www.alliance-cxca.org.
4. Data derived from official reports by WHO, United Nations, Cervical Cancer Global Crisis Card.~~, ~~The World Bank, IARC Globocan. http://www.who.int/hpvcentre/statistics/en/.
5. Campbell C. Assessment of Cervical Cancer Incidence, Histopathology, and Screening Practices Among Hispanic Women in Latin America and Michigan. ~~,~~ ~~a~~ A dissertation for the degree of Doctor of Philosophy, 2011.
6. Zohre Momenimovahed and Hamid Salehiniya, Incidence, mortality and risk factors of cervical cancer in the world.~~,~~ Biomedical Research and Therapy, BioMedPress, 2018, http://www.bmrat.org/index.php/BMRAT/article/view/386.
7. Rose I Anorlu, Cervical cancer: the sub-Saharan African perspective.~~,~~ Reproductive Health Matters, 2008;16(32).
8. Aswathy Sreedevi, Reshma Javed, and Avani Dinesh, Epidemiology of cervical cancer with special focus on India.~~,~~ Int J Womens Health. 2015; 7: 405–414, doi: 10.2147/IJWH.S50001.
9. Amos D. Mwaka, Christopher G. Orach, Edward M. Were, Georgios Lyratzopoulos, Henry Wabinga, Martin Roland, Awareness of cervical cancer risk factors and symptoms: cross-sectional community survey in post-conflict northern Uganda.~~,~~ Health Expect. 2016 Aug; 19(4): 854–867, doi: 10.1111/hex.12382.
10. Ginindza TG, Almonte M, Dlamini X, Sartorius B. Distribution of cervical abnormalities detected by visual inspection with acetic acid in Swaziland, 2011-2014: A retrospective study.~~,~~ African J Prim Heal care Fam Med. 2018;10(1):e1-e7.~~,~~ doi:10.4102/phcfm.v10i1.1773.

Formatted: Line spacing: 1.5 lines

Field Code Changed

Field Code Changed

Field Code Changed

Field Code Changed

Formatted: Font: Not Italic

11. Nkfusai NC, Mubah TM, Yankam BM, Tambe TA, Cumber SN. Prevalence of precancerous cervical lesions in women attending Mezam Polyclinic Bamenda, Cameroon~~.~~. Pan Afr Med J. 2019; 32:174. doi:10.11604/pamj.2019.32.174.16895.

12. Anggraeni TD, Peters AAW. Prevalence , Age Distribution , and Risk Factors of Visual Inspection With Acetic Acid-Positive From 2007 to 2011 in Jakarta. 2017;22(2):103-107.~~.~~

13. Kahesa C, Kjaer SK, Ngoma T, et al. Risk factors for VIA positivity and determinants of screening attendances in Dar es Salaam , Tanzania. BMC Public Health. 2012; 12(1):1. doi:10.1186/1471-2458-12-1055.

14. Haile T. Beyene M. Berg A. prevention of cervical cancer in Ethiopia, ~~Senamoksh~~ a report from Women´s Health Center, Addis Ababa, Ethiopia and ESOG, Ethiopia, a model clinic in Addis Ababa, 2018.

15. ~~Dye D et al., 2009)~~ Dye D., Solomon B., Claire H., ~~Yared T., Vanessa H., Teshome D., Marion B., Anne R., (2009)~~ et al, Complex care systems in developing countries, Breast Cancer Patient Navigation in Ethiopia,2009, 116:577–85.).

16. Birhanu Z. et al, Health seeking behavior for cervical cancer in Ethiopia: a qualitative study, International Journal for Equity in Health, BioMed center, 2012, 11:83.

17. Roza Teshome Kassa, Risk factors associated with precancerous cervical lesion among women screened at Marie Stops Ethiopia, Adama town, Ethiopia: a case control study, BMC Res Notes, ~~(~~2018,~~)~~ 11:145, https://doi.org/10.1186/s13104-018-3244-6.

18. Teame H, Addissie A, Ayele W, Hirpa S, Gebremariam A, Gebreheat G, et al. ~~(2018)~~ Factors associated with cervical precancerous lesions among women screened for cervical cancer in Addis Ababa, Ethiopia: A case control study~~.~~ PLoS ONE, , 2018. 13(1): e0191506. https://doi.org/10.1371/journal.pone.0191506.

19. Deksissa ZM, Tesfamichael FA, Ferede HA. Prevalence and factors associated with VIA positive result among clients screened at Family Guidance Association of Ethiopia, south west area office, Jimma model clinic, Jimma, Ethiopia ~~2013~~: A cross-sectional study, Womens Health. BMC Res Notes. 2015; 8(1):8-13. doi:10.1186/s13104-015-1594.~~x~~

20. Kebede Haile Misgina, Hailay Seyoum Belay and Teklehaymanot Huluf Abraha, Prevalence of precancerous cervical lesion and associated factors among women in North

Formatted: Font: Not Italic

Formatted: Font: Not Italic

Formatted: Font: Not Italic

Formatted: Font: Not Bold

Formatted: Font: Not Italic

Field Code Changed

Field Code Changed

Formatted: Font: Not Italic

Ethiopia, Journal of Public Health and Epidemiology, 2017, Vol. 9(3), pp. 46-50, DOI: 10.5897/JPHE2016.0891

21. Bayu H, Berhe Y, Mulat A, Alemu A (2016) Cervical Cancer Screening Service Uptake and Associated Factors among Age Eligible Women in Mekelle Zone, Northern Ethiopia, 2015:: A Community Based Study Using Health Belief Model. PLoS ONE, 2016, 11(3): e0149908. doi:10.1371/journal.pone.0149908

22. Yeneabeba Tilahun, National Cervical Cancer Prevention Training Package Participant Manual, Federal Ministry of Health Ethiopia, 2015.

23. Federal Democratic Republic of Ethiopia Ministry of Health. *Guideline for Cervical Cancer Prevention and Control in Ethiopia*.; 2015. https://www.iccp-portal.org/system/files/plans/Guideline Eth Final.pdf.

24. *Seyoum T, Yesuf A, Kejela G, Gebremeskel F (2017) Utilization of Cervical Cancer Screening and Associated Factors among Female Health Workers in Governmental Health Institution of Arba Minch Town and Zuria District, Gamo Gofa Zone, Arba Minch, Ethiopia, 2016. Arch Cancer Res. Vol.5 No.4:165*

23.25. *Cecilia Chinemerem Nwabichie, Rosliza Abdul Manaf*, Suriani Binti Ismail, Factors Affecting Uptake of Cervical Cancer Screening Among African Women in Klang Valley, Malaysia, Asian Pac J Cancer Prev, 19 (3), 825-831*

Formatted: Font: Not Bold

Formatted: Font: (Default) Times New Roman, 12 pt, Not Italic, Font color: Auto

Epidemiology of Cervical ~~precancerous~~ Precancerous ~~cervical lesion~~ Lesion and Risk Factors Among Adult Women In Tigray, Ethiopia

Authors:

Gerezgiher B. Abera[1*], Henock G. Yebyo[2], Haftamu Hailekiros[3], Selam Niguse[3], Yibrah Berhe[3], Goitom Gigar[4], Tsehaye Asmelash[5], Gelila Goba[6]

Affiliation:

[1]School of Nursing, College of Health Sciences, Mekelle University, Mekelle, Ethiopia;

[2]School of Public Health, College of Health Sciences, Mekelle University, Mekelle, Ethiopia;

[3] School of Medicine, College of Health Sciences, Mekelle University, Mekelle, Ethiopia;

[4] Tigray Regional Health Bureau, Mekelle, Ethiopia;

[5]College of Health Sciences, Aksum University, Axum", Ethiopia;

[6]Department of Obstetrics and Gynecology, University of Illinois at Chicago, Chicago, USA

*__Corresponding Author__: E-mail: gbamsc2002@gmail.com

ORCID ~~iD~~ ID https://orcid.org/0000-0003-4968-2885

Abstract

Background: Cervical cancer is a preventable disease if ~~detected~~ treated early, but remains the second leading cause of cancer-related mortality among women in low- and middle-income countries. Data on epidemiology and risk factors in these settings are scarce. This study aimed to assess the ~~magnitude~~ prevalence of precancerous lesions and risk factors in Tigray region, Ethiopia.

Methods: A community-based cross sectional study was used and 900 participants were ~~selected~~ recruited using multistage sampling and 883 were ressponeded correctly. Data were collected using an interviewer administered questionnaire and screening with visual inspection with ascetic acid. Multinomial logistic regression analysis was conducted to estimate predictors.

Results: Seventy-nine (8.95%) women were positive for pre-cancer lesion and 35 (3.96%) were suspicious for cervical cancer. Divorced or widowed women had ~~risked~~ 2.5 and 4.7 times more ~~likely~~ risk to be positive and suspicious~~,~~ respectively, compared to single women (rrr=2.5, 95% CI [1.13, 5.52]); (rrr=4.69, 95% CI [1.00, 21.84]). The risk of having a suspicious result was 68% lower for women with primary education compared to those with no formal education (rrr=0.32, 95% CI [1.00, 21.84]). History of sexually transmitted infection was associated with positive pre-cancer lesion (rrr=1.91, 95% CI [1.11, 3.27]) whereas, being farmer (rrr=4.83, 95% CI [1.44, 16.13]), merchant (rrr=4.85, 95% CI [1.52, 15.46]), bleeding between periods (rrr =3.26, 95% CI [1.32, 8.04]) and pelvic or back pain (rrr =2.79, 95% CI [1.18, 6.58]) were associated with suspicious for cancer.

Conclusion: About 8.9% and 4% of the women were positive and suspicious for pre-cancer cervical lesion, respectively. Marital status, education, sexually transmitted infection, bleeding, and pelvic pain were predictors of cervical precancerous lesions.

Key words: Epidemiology, Cervical, Pre-cancerous lesions.

Introduction

Cervical cancer (CC) is a public health problem worldwide [1], primarily in low and middle-income countries, though data are scarce [2, 3]. The disease is fatal, but preventable if ~~detected~~ treated early [4]. The World Health Organization (WHO) indicates that CC kills an estimated 275,000 women every year and 500,000 new cases reported worldwide [1]. CC is the second largest ~~cancer~~ killer among cancer-related death of women in low-and-middle income countries (LMICs) [~~5~~4]. Reports from different countries show that the incidence of CC is high in developing countries and sexually transmitted infections (STIs), hormonal influences, genetics and ~~host~~ participant factors are risk factors for CC [5, 6]. In Sub-Saharan Africa, an estimated 57,000 cases of CC occurred in 2000, comprising 22.2% of all female cancers [7].

A study from India revealed that late-stages reporting of disease was common with a peak age of CC incidence from 55 to 59 years and early marriage, multiple sexual partners, multiple pregnancies, oral contraceptives, and lack of awareness were significant risk factors [8]. A study in Uganda showed that 99% of women had heard about CC and 63% believed that family planning was a cause of CC as well as 85% recognizing inter-menstrual bleeding as a symptom of CC [9]. Other studies have also revealed the burden and public health importance of CC in LMICs [10-13].

In Ethiopia, cervical cancer is the most common cause of cancer death among female cancers with an overall mortality of 70% [14]. A study in Ethiopia showed that the mean age at diagnosis with cervical cancer was 48 years [15]. In a community based cross-sectional survey conducted in Gondar, northwest Ethiopia (2010), only 31% of respondents were knowledgeable about risk factors, symptoms, treatment options, prevention and early detection measures for cervical

cancer [16]. Studies from Addis Ababa, Adama and Jimma showed that women 40 - 49 years of age, having a history of STIs, having two or more lifetime sexual partners and contraceptive use were risk factors of the cervical pre-cancerous lesion [17- 19]. Another institution based studies in Tigray revealed a prevalence of precancerous cervical lesion of 6.7% and that STIs are predictors of the precancerous cervical lesions [20, 21].

The Ethiopian Ministry of Health officially launched guideline for cervical cancer prevention and control in 2005, describes a "See and Treat approach" using visual inspection with acetic acid (VIA) screening and cryotherapy treatment for the reason of feasibility and affordability (22).

Although Ethiopia's federal ministry of health (FMoH) has given priority for CC prevention and control, there is a scarcity of data related to precancerous cervical lesions. Existing guidelines call for community based precancerous cervical lesions epidemiology and risk factors [22]. Accordingly, this community-based research determines the prevalence and factors associated with precancerous cervical lesions among adult women in Tigray, Ethiopia.

Method

Study setting and design

We conducted a community-based, cross-sectional study in Tigray, Northern Ethiopia. Data ~~was~~ were collected from March 2016 to June 2017. The study was conducted in Tigray region, which has ~~covered~~ seven zones, 34 rural and 18 urban districts ('Woredas' in local name) and 763 sub-districts ('Kebele/Tabia' in local name). In 2017, the Tigray Region had one specialized hospital, 14 general hospitals, 22 primary hospitals, 214 health centers, and 712 health posts as well as more than 500 private health facilities.

~~**Sample size and sampling technique:**~~

~~The sample size was calculated with the aim of estimating a precise population prevalence of precancerous cervical lesions in the Tigray Region. As there is no baseline prevalence study, we assumed a 0.5 probability of the disease, 0.05 probability of alpha testing, 80% power and design effect of 2 accounting for two-stage sampling. We added 10% to the calculated sample size to account for non-responses, yielding a sample size of 846, which we rounded to 900 for convenience.~~

~~A multistage sampling was used to select the study subjects. Considering 40% of the 52 districts in the region, 21 of the districts which were distributed proportionally to zones were selected randomly. Since there are 315 sub-districts in the selected districts, 20% of the sub-districts (that were 63 sub-districts) were distributed proportionally to the selected districts, and were selected randomly. Study participants were allocated proportionally to sub-districts based on the number of participants fulfilling the eligibility criteria. The study participants were finally selected using~~

~~systematic random sampling and invited for screening to a total of 17 primary and general hospitals in the sub districts.~~

Study Population

Study participants: All adult women living in Tigray region for about six months or more were considered as a source population, and those who were voluntary and selected as study subjects were the study population.

***Eligibility criteria**: All adult women, who were sexually active and voluntarily consented to participate were included in the study. Those with history of hysterectomy were excluded from the study.*

Sample size and sampling technique:

The sample size was calculated with the aim of estimating a precise population prevalence of precancerous cervical lesions in the Tigray Region. As there is no baseline study of prevalence of precancerious cervical lesion, we assumed a 0.5 probability of the disease, 0.05 probability of alpha testing, 80% power and design effect of 2 accounting for two-stage sampling. We added 10% to the calculated sample size to account for non-responses, yielding a sample size of 846, which we rounded to 900 for convenience.

A multistage sampling was used to select the study subjects. Considering 40% of the 52 districts in the region, 21 of the districts which were distributed proportionally to zones were selected randomly. Since there are 315 sub-districts in the selected districts, 20% of the sub-districts (a total of 63 sub-districts) were selected randomly. Study participants were allocated proportionally to sub-districts based on the number of participants fulfilling the eligibility criteria in each sites. This was taken from the health extension workers registration book.

Formatted: No bullets or numbering, Don't adjust space between Latin and Asian text, Don't adjust space between Asian text and numbers

Formatted: Font: Bold

Formatted: Font: Bold

Formatted: Justified, Don't adjust space between Latin and Asian text, Don't adjust space between Asian text and numbers

Formatted: Font: Not Bold, Italic

Formatted: Font: Italic

Recruitment process: A large mobilization was preceded the screening. This effort entailed training of Health Extension Workers (HEWs) from selected sub-districts. HEWs in turn oriented women in their communities. They also mobilized Women Development Army (WDA) leaders and members as well as sub-district leaders by providing brief orientations about cervical cancer (CC) as well as upcoming screening dates and locations. Grassroots mobilization was complimented by promotion on regional radio stations by the Tigray Regional Health Bureau (TRHB).

Following community mobilization, participants were invited for screening to a total of 17 primary and general hospitals in the sub districts which have cervical cancer screening cencers using visual inspection with acetic acid (VIA). As per the estimated number of expected participants, a key value of 4 was calculated. Every forth women arriving at the hospitals following grassroots mobilization were selected and invited to participate in the study. Finally, those who fulfill the eligibility criteria were recruited as a study subject.

Formatted: Font: Bold

Data collection procedure:

~~A large mobilization effort preceded screening. This effort entailed training Health Extension Workers (HEWs) from selected sub-districts. HEWs in turn oriented women in their communities. They also mobilized Women Development Army (WDA) leaders and members as well as sub-district leaders by providing brief orientations about CC as well as upcoming screening dates and locations. Grassroots mobilization was complimented by promotion on regional radio stations by the Tigray Regional Health Bureau (TRHB).~~

~~Women arriving at selected hospitals following grassroots mobilization were invited to participate in the study. Consent was voluntary and women were given the right of refusal or withdrawal at any point.~~ Women providing consent were interviewed using an interviewer

administered questionnaire to obtain socio-demographic and risk-factors information, including health related behaviors and lifestyle, knowledge, attitude, and clinical attributes.

Clinical testing was conducted by certified nurses trained by the TRHB according to the national cervical cancer prevention guidelines [23]. A Visual Inspection with Acetic Acid (VIA) screening protocol was developed based on the national guideline, The protocol included private exam area, examination table, trained health professionals, adequate light source, sterile vaginal speculum, new examination or surgical gloves, large cotton swabs, diluted (3-5%) acetic acid, small bowl, containers with 0.5% chlorine solution, plastic bucket with a plastic bag and quality assurance system to maximize accuracy.

P~~Epidemiology of p~~recancerous cervical lesions was determined by vaginal speculum exam during which provider applied diluted acetic acid to the cervix. Visual confirmation of color change by the naked eye was used to determine the presence or absence of lesions. The test was considered positive if a sharp, distinct, well-defined, dense (opaque/dull or oyster) Aceto-white area was present with raised margins touching the Squamo-columnar junction (SCJ). The test was considered negative if no or faint Aceto-white lesions and/or smooth surface was present. The test was cinsudered suspicious for CC where clinically visible ulcerative, cauliflower-like growth or ulcer, oozing, and/or bleeding on touch were observed.

Data analysis

Data was analyzed using STATA version14. Data entry and clearance was done using descriptive analysis to check if any missing and inappropriate categories. Descriptive statistics were presented in texts and tables. The dependent variable was cervical pre-cancer status, in which the screening result had three possible outcomes: Negative, Positive and Suspicious for VIA. A multinomial regression model was used to check the influence of socio-demographic,

risk factors and reproductive health characteristics on CC screening outcomes. Unadjusted multinomial regression was used to examine the separate bivariate relationship between each ~~predictor~~ independent variables and the dependent variable. Relative risk ratios (rrr) were used to assess the multivariate association of the predictor variables ~~on~~ with the dependent variable.

Ethical consideration: Before beginning data collection ethicalclearance was obtained from mekelle university, collage of health sience research and community service commiyyee and support letter was obtained from Tigray regional health bureau. Participants were oriented the general objective of the study, procedure of data collection, privacy and confidenciality of the information obtained, it was also informed that participation is voluntary based and the possibility to withdraw at any time when they need. Finally written informed consent was obtained from the participants prior to the actual data collection.

Formatted: Font: Bold

Formatted: Justified, Space After: 10 pt, Line spacing: 1.5 lines

Results

Characteristics of the study participants

A total of 883 questionnaires was collected with complete information, equating to a response rate of 98.1%. More than half of the participants 496 (56.2%) came from urban districts. Mean age of participants was 35.69 years (SD ±8.45, range 16 - 62). Among all respondents, 532 (60.2%) were married, 723 (81.9%) were Orthodox Christian, 286 (32.4%) had no formal education, 282 (31.9%) had no formal work, and 717 (81.2%) had zero to four children. Median income was Ethiopian Birr 1,500 and 515 (58.3%) women earned between Ethiopian Birr 0 and 2,000 per month [Table 1].

Table 1. Distribution of Socio-demographic characteristics by VIA results among women of reproductive age in Tigray, (n=883)

**Variables**	**Groups**	**VIA results**			
		**Negative** N (%)	**Positive** N (%)	**Suspicious** N (%)	**Total** N (%)
	Rural	345(89.1)	28(7.2)	14(3.6)	387(43.8)
Residence	Urban	424(85.5)	51(10.3)	21(4.2)	496(56.2)
	15-30	257(86.5)	30(10.1)	10(3.4)	297 (33.6)
Age (yr)	31-45	416(87.6)	44(9.3)	15(3.2)	475 (53.8)
	>=46	96(86.5)	5(4.5)	10(9.0)	111 (12.6)
	Single	208(92.0)	14(6.2)	4(1.8)	226(25.6)
Marital status	Married	472(88.7)	42(7.9)	18(3.4)	532(60.2)
	Divorced/Others	89(71.2)	23(18.4)	13(10.4)	125(14.2)
	Orthodox	627(86.7)	64(8.9)	32(4.4)	723(81.9)
Religion	Muslim and others	142(88.8)	15(9.4)	3(1.9)	160(18.1)
	No formal education	240(83.9)	25(8.7)	21(7.3)	286(32.4)
Educational status	Primary education	325(89.0)	30(8.2)	10(2.7)	365(41.3)
	Secondary education	116(85.3)	17(12.5)	3(2.2)	136(15.4)
	Tertiary education	88(91.7)	7(7.3)	1(1.0)	96(10.9)
	Civil servant	209(93.3)	11(4.9)	4(1.8)	224(25.4)
Occupation	Farmer	141(81.5)	21(12.1)	11(6.4)	173(19.6)
	Merchant	172(84.3)	18(8.8)	14(6.9)	204(23.1)
	No formal work	247(87.6)	29(10.3)	6(2.1)	282(31.9)
	0-2000	439(85.2)	51(9.9)	25(4.9)	515(58.3)
	2001-4000	158(86.3)	16(8.7)	9(4.9)	183(20.7)
	4001-6000	105(92.1)	8(7.0)	1(0.9)	114(12.9)
	6001-8000	16(88.9)	2(11.1)	0(0.0)	18(2.0)
Monthly income	8001-10000	51(96.2)	2(3.8)	0(0.0)	53(6.0)
	0-4 children	628(87.6)	67(9.3)	22(3.1)	717(81.2)
Parity	5-12 children	141(84.9)	12(7.2)	13(7.8)	166(18.79)

Cervical ~~Precancerous~~ precancerous ~~cervical~~ lesion:

A total of 782 (88.6%) had visible SCJ. Among all the participants, 79 (8.9%) were positive for VIA, 35 (4.0%) were suspicious for cancer, and 769 (87.1%) were negative for pre-cancer lesions. A total of 776 (87.9%) reported that their age at first sexual contact was less than 20 years. Of the total, 344 (39.0%) respondents had more than two sexual partners in their lifetime and 199 (22.5%) experienced STIs. Thirty-two (3.6%) were smokers and one-third of respondents consumed alcohol 255 (28.9%). Corticosteroids were used in 97 (11.0%) women and 199 (22.5%) where current contraceptive users. Seventy (7.9%) had a family history of CC. Out of these women, nine (12.9%) were VIA-positive [Table 2].

Table. 2. Distribution of risk factors by VIA results among women of reproductive age in Tigray, (n=883).

Risk factors of cervical cancer		VIA results			Total N (%)
		Negative N (%)	Positive N (%)	Suspicious N (%)	
Age at first sexual contact	≤ 20 years age	675(87.0)	66(8.5)	35(4.5)	776(87.9)
	> 20 years age	94(87.9)	13(12.1)	0(0.0)	107(12.1)
Number Sexual Partner	Only one	461(88.3)	45(8.6)	16(3.1)	522(59.1)
	Two or more	293(85.2)	33(9.6)	18(5.2)	344(39.0)
	No or unspecified	15(88.2)	1(5.9)	1(5.9)	17(1.9)
History of STI	Yes	156(78.4)	32(16.1)	11(5.5)	199(22.5)
	No	613(89.6)	47(6.9)	24(3.5)	684(77.5)
Active smoking	Yes	29(90.6)	2(6.2)	1(3.1)	32(3.6)
	No	740(87.0)	77(9.0)	34(4.0)	851(96.4)
Alcohol consumption	Yes	232(91.0)	18(7.1)	5(2.0)	255(28.9)
	No	537(85.5)	61(9.7)	30(4.8)	628(71.1)
Chronic corticosteroids use	Yes	86(88.7)	8(8.2)	3(3.1)	97(11.0)
	No	683(86.9)	71(9.0)	32(4.1)	786(89.0)
Current contraceptive use	Yes	169(84.9)	21(10.6)	9(4.5)	199(22.5)
	No	600(87.7)	58(8.5)	26(3.8)	684(77.5)
Family history of cervical cancer	Yes	60(85.7)	9(12.9)	1(1.4)	70(7.9)
	No	709(87.2)	70(8.6)	34(4.2)	813(92.1)

From the study participants, 183 (20.7%) women experienced bleeding between menstruation, 94 (10.6%) experienced heavy menstruation, 176 (19.9%) experienced contact bleeding and 152

(17.2%) women experienced pain during sex. Pelvic or back pain was reported by 267 (30.2%) women [Table 3].

Table 3. Distribution of signs and symptoms by VIA results among women of reproductive age in Tigray, (n=883).

Sign and symptoms and cervical cancer status		VIA results			
		Negative N (%)	Positive N (%)	Suspicious N (%)	N (%)
Bleeding between menstruation	Yes	149(81.4)	16(8.7)	18(9.8)	183(20.7)
	No	620(88.6)	63(9.0)	17(2.4)	700(79.3)
Heavy menstruation	Yes	70(74.5)	12(12.8)	12(12.8)	94(10.6)
	No	699(88.6)	67(8.5)	23(2.9)	789(89.4)
Bleeding after menopause	Yes	78(77.2)	14(13.9)	9(8.9)	101(11.4)
	No	691(88.4)	65(8.3)	26(3.3)	782(88.6)
Contact bleeding	Yes	143(81.2)	19(10.8)	14(8.0)	176(19.9)
	No	626(88.5)	60(8.5)	21(3.0)	707(80.1)
Pain during sex	Yes	120(78.9)	23(15.1)	9(5.9)	152(17.2)
	No	649(88.8)	56(7.7)	26(3.6)	731(82.8)
Pelvic or back pain	Yes	213(79.8)	33(12.4)	21(7.9)	267(30.2)
	No	556(90.3)	46(7.5)	14(2.3)	616(69.8)
Unusual vaginal discharge	Yes	239(79.9)	37(12.4)	23(7.7)	299(33.9)
	No	530(90.8)	42(7.2)	12(2.1)	584(66.1)

Factors associated with cervical cervical precancerous lesions screening VIA result:

In the multinomial analysis "Negtive result of the dependent variable was used as a base reference against the positive and suspicious results; hence the relative risk is relative to those who have negative results.

Multinomial bivariate analysis revealed that age, marital and education status, occupation, symptoms of CC (multiple), parity, STI history of partner, and lower abdominal pain were significantly associated with positive or suspicious results (p-value < 0.05). Other variables were not significantly associated with cervical precancerous cervical lesions. The multivariate logistic regression analysis showed that marital and educational status, occupation, history of STIs, bleeding between menstruation, and pelvic or back pain were significantly associated with the presence of cervical precancerous cervical lesions. Divorced and or widowed women were had

2.5 and 4.7 times ~~as likely,~~risk ~~respectively~~, to be VIA-positive ~~or~~ and suspicious respectively compared to single women (rrr=2.50, 95% CI [1.13, 5.52]); (rrr=4.69, 95% CI [1.00, 21.84]). The risk of being suspicious was 68% lower for women with primary education compared to those with no formal education (rrr=0.32, 95% CI [1.00, 21.84]). The risk of being suspicious for cancer was 4.8 times higher among farmers (rrr=4.83, 95% CI [1.44, 16.13]) and merchants (rrr=4.85, 95% CI [1.52, 15.46]) compared to respondents with no formal work.

The risk of being VIA-positive was 90% higher among women with a history of STIs (rrr=1.91, 95% CI [1.11, 3.27]). The risk of being suspicious for cancer was 3.26 times higher among women who experienced bleeding between periods (rrr =3.26, 95% CI [1.32, 8.04]). Women with a history of pelvic or back pain were 2.7 times more likely to be suspicious for cancer (rrr =2.79, 95% CI [1.18, 6.58]) [Table 4].

Table.4. Bivariate and Multivariable analysis of independent variables with cervical cancer status among reproductive age women in Tigray, (n=883).

~~Independent~~	~~VIA result~~	~~Indicators~~	~~CRRR [95%~~	~~P-value~~	~~ARRR [95%~~	~~P-value~~
~~Age~~	~~Positive~~	~~15-30~~	~~1~~			
		~~31-45~~	~~0.91 (0.56, 1.48)~~	~~0.693~~	~~0.91(0.53,~~	~~0.737~~
		~~>=46~~	~~0.45(0.17, 1.18)~~	~~0.105~~	~~0.50(0.17,~~	~~0.205~~
	~~Suspicious~~	~~15-30~~	~~1~~			
		~~31-45~~	~~0.93(0.410, 2.09)~~	~~0.855~~	~~0.58(0.22,~~	~~0.285~~
		~~>=46~~	~~2.68(1.08, 6.63)~~	~~0.033~~	~~2.34(0.74,~~	~~0.143~~
~~Marital status~~	~~Positive~~	~~Single~~	~~1~~		~~1~~	
		~~Married~~	~~1.32(0.71, 2.47)~~	~~0.382~~	~~0.94(0.46,~~	~~0.867~~
		~~Divorced/widowed~~	~~3.84(1.89, 7.80)~~	~~0.000~~	~~2.50(1.13,~~	~~0.023~~
	~~Suspicious~~	~~Single~~	~~1~~		~~1~~	
		~~Married~~	~~1.98(0.66, 5.93)~~	~~0.221~~	~~2.15(0.51,~~	~~0.292~~
		~~Divorced/widowed~~	~~7.59(2.41, 23.93)~~	~~0.001~~	~~4.69(1.00,~~	~~0.049~~
~~Educational status~~	~~Positive~~	~~No formal education~~	~~1~~		~~1~~	
		~~Primary Education~~	~~0.89(0.51, 1.55)~~	~~0.67~~	~~0.78(0.41,~~	~~0.462~~
		~~Secondary~~	~~1.41(0.73, 2.71)~~	~~0.307~~	~~1.15(0.54,~~	~~0.708~~
		~~Tertiary Education~~	~~0.76(0.32, 1.83)~~	~~0.545~~	~~0.84(0.29,~~	~~0.753~~
	~~Suspicious~~	~~No formal education~~	~~1~~		~~1~~	
		~~Primary Education~~	~~0.35(0.17, 0 .76)~~	~~0.000~~	~~0.32(0.11, 0.91)~~	~~0.034~~
		~~Secondary~~	~~0.29(0.09, 1.01)~~	~~0.052~~	~~0.25(0.05,~~	~~0.066~~
		~~Tertiary Education~~	~~0.13(0.02, 0.98)~~	~~0.048~~	~~0.12(0.01,~~	~~0.093~~
~~Occupation~~	~~Positive~~	~~Civil Servant~~	~~0.45(0.22, 0.92)~~	~~0.029~~	~~0.78(0.41,~~	~~0.462~~
		~~Farmer~~	~~1.26(0.69, 2.30)~~	~~0.436~~	~~1.15(0.54,~~	~~0.708~~

		Merchant	0.89(0.47, 1.65)	0.716	0.84(0.29,	0.753
		No formal work	1		1	
	Suspicious	Civil Servant	0.78(0.22, 2.82)	0.715	2.67(0.48,	0.257
		Farmer	3.21(1.16, 8.87)	0.024	4.83(1.44,	0.011
		Merchant	3.35(1.26, 8.89)	0.015	4.85(1.52,	0.008
		No formal work	1		1	
Parity	Positive	0-4 children	1		1	
		5-12 children	0.79(0.42, 1.51)	0.490	0.85(0.40,	0.680
	Suspicious	0-4 children	1		1	
		5-12 children	2.63(1.29, 5.35)	0.008	1.62(0.61,	0.327
History of STI	Positive	Yes	2.67(1.65, 4.33)	0.000	1.91(1.11,	0.018
		No	1		1	
	Suspicious	Yes	1.80(0.86, 3.75)	0.117	0.99(0.40,	0.992
		No	1		1	
Blood between period	Positive	Yes	1.05(0.59, 1.88)	0.851	0.71(0.35,	0.353
		No	1		1	
	Suspicious	Yes	4.40(2.21, 8.75)	0.000	3.26(1.32,	0.010
		No	1		1	
Heavy menstruation	Positive	Yes	1.78(0.92, 3.46)	0.085	1.23(0.55,	0.612
		No	1		1	
	Suspicious	Yes	5.20(2.48, 10.9)	0.000	2.45(0.92,	0.072
		No	1		1	
Bleeding after menopause	Positive	Yes	1.90 (1.02, 3.55)	0.042	1.25(0.57,	0.572
		No	1		1	
	Suspicious	Yes	3.06 (1.38, 6.77)	0.006	1.27(0.40,	0.682
		No	1		1	
Contact bleeding	Positive	Yes	1.38(0.80, 2.39)	0.242	0.85(.42, 1.69)	0.644
		No	1		1	
	Suspicious	Yes	2.91(1.44, 5.87)	0.003	0.81(0.30,	0.688
		No	1		1	
Pain during sex	Positive	Yes	2.22(1.31, 3.74)	0.003	1.39(0.70,	0.338
		No	1		1	
	Suspicious	Yes	1.87(0.85, 4.09)	0.116	0.69(0.23,	0.501
		No	1		1	
Pelvic or back pain	Positive	Yes	1.87(1.16, 3.00)	0.010	1.60(0.90,	0.109
		No	1		1	
	Suspicious	Yes	3.91(1.95, 7.84)	0.000	2.79(1.18,	0.019
		No	1		1	
Unusual vagina discharge	Positive	Yes	1.95(1.22, 3.11)	0.005	1.39(0.78,	0.260
		No	1		1	
	Suspicious	Yes	4.26 (2.08, 8.68)	0.000	2.22(0.91,	0.076
		No	1		1	

Variables			VIA results					
		Negati	Positive			Suspicious		
		N(%)	N(%)	cRRR [95% CI]	aRRR [95%	N(%)	CRRR [95% CI]	ARRR [95% C.I.)
Age	15-30	257(86.5)	30(10.1)	1	0.91(0.53, 1.55)	14(3.6)	1	0.58(0.22, 1.55)
	31-45	416(87.6)	44(9.3)	0.91 (0.56, 1.48)	0.50(0.17, 1.45)	21(4.2)	0.93(0.410, 2.09)	2.34(0.74, 7.36)
	>=46	96(86.5)	5(4.5)	0.45(0.17, 1.18)	0.91(0.53, 1.55)	10(3.4)	2.68(1.08, 6.63) *	0.58(0.22, 1.55)
Marital status	Single	208(92.0)	14(6.2)	1	1	4(1.8)	1	1
	Married	472(88.7)	42(7.9)	1.32(0.71, 2.47)	0.94(0.46, 1.91)	18(3.4)	1.98(0.66, 5.93)	2.15(0.51, 8.97)
	Divorced/widowed	89(71.2)	23(18.4	3.84(1.89, 7.80)***	2.50(1.13, 5.52)*	13(10.4)	7.59(2.41, 23.93)***	4.69(1.00, 21.84)*
Educational	No formal education	240(83.9)	25(8.7)	1	1	21(7.3)	1	1

status	Primary Education	325(89.0)	30(8.2)	0.89(0.51, 1.55)	0.78(0.41, 1.48)	10(2.7)	0.35(0.17, 0 .76) ***	0.32(0.11, 0.91)*
	Secondary Education	116(85.3)	17(12.5)	1.41(0.73, 2.71)	1.15(0.54, 2.43)	3(2.2)	0.29(0.09, 1.01)	0.25(0.05, 1.09)
	Tertiary Education	88(91.7)	7(7.3)	0.76(0.32, 1.83)	0.84(0.29, 2.39)	1(1.0)	0.13(0.02, 0.98)*	0.12(0.01, 1.41)
Occupation	Civil Servant	209(93.3)	11(4.9)	0.45(0.22, 0.92)*	0.78(0.41, 1.48)	4(1.8)	0.78(0.22, 2.82)	2.67(0.48, 14.68)
	Farmer	141(81.5)	21(12.1)	1.26(0.69, 2.30)	1.15(0.54, 2.43)	11(6.4)	3.21(1.16, 8.87)*	4.83(1.44, 16.13)*
	Merchant	172(84.3)	18(8.8)	0.89(0.47, 1.65)	0.84(0.29, 2.39)	14(6.9)	3.35(1.26, 8.89)*	4.85(1.52, 15.46)**
	No formal work	247(87.6)	29(10.3)	1	1	6(2.1)	1	1
Parity	0-4 children	628(87.6)	67(9.3)	1	1	22(3.1)	1	
	5-12 children	141(84.9)	12(7.2)	0.79(0.42, 1.51)	0.85(0.40, 1.79)	13(7.8)	2.63(1.29, 5.35)**	1.62(0.61, 4.25)
History of STI	Yes	156(78.4)	32(16.1)	2.67(1.65, 4.33)***	1.91(1.11, 3.27)*	11(5.5)	1.80(0.86, 3.75)	0.99(0.40, 2.42)
	No	613(89.6)	47(6.9)	1	1	24(3.5)	1	1
Blood between period	Yes	149(81.4)	16(8.7)	1.05(0.59, 1.88)	0.71(0.35, 1.45)	18(9.8)	4.40(2.21, 8.75)***	3.26(1.32, 8.04)*
	No	620(88.6)	63(9.0)	1	1	17(2.4)	1	1
Heavy menstruation	Yes	70(74.5)	12(12.8)	1.78(0.92, 3.46)	1.23(0.55, 2.74)	12(12.8)	5.20(2.48, 10.9)***	2.45(0.92, 6.52)
	No	699(88.6)	67(8.5)	1	1	23(2.9)	1	1
Bleeding after menopause	Yes	78(77.2)	14(13.9)	1.90 (1.02, 3.55)*	1.25(0.57, 2.71)	9(8.9)	3.06 (1.38, 6.77) **	1.27(0.40, 3.99)
	No	691(88.4)	65(8.3)	1	1	26(3.3)	1	1
Contact bleeding	Yes	143(81.2)	19(10.8)	1.38(0.80, 2.39)	0.85(.42, 1.69)	14(8.0)	2.91(1.44, 5.87)**	0.81(0.30, 2.19)
	No	626(88.5)	60(8.5)	1	1	21(3.0)	1	1
Pain during sex	Yes	120(78.9)	23(15.1)	2.22(1.31, 3.74)**	1.39(0.70, 2.74)	9(5.9)	1.87(0.85, 4.09)	0.69(0.23, 2.01)
	No	649(88.8)	56(7.7)	1	1	26(3.6)	1	1
Pelvic or back pain	Yes	213(79.8)	33(12.4)	1.87(1.16, 3.00)*	1.60(0.90, 2.85)	21(7.9)	3.91(1.95, 7.84)***	2.79(1.18, 6.58)*
	No	556(90.3)	46(7.5)	1	1	14(2.3)	1	1
Unusual vagina discharge	Yes	239(79.9)	37(12.4)	1.95(1.22, 3.11)**	1.39(0.78, 2.49)	23(7.7)	4.26 (2.08, 8.68)***	2.22(0.91, 5.38)
	No	530(90.8)	42(7.2)	1	1	12(2.1)	1	1

Discussion

Ethiopia's Federal Ministry of Health has prioritized CC prevention and treatment [23]; however, there is a scarcity of data related to the prevalence of precancerous cervical lesions and abnormal screening results. This study explored the prevalence of precancerous lesions and abnormal results in Tigray, Northern Ethiopia.

Our study showed that 79 (8.9%) respondents were VIA-positive and 35 (4.0%) were suspicious for CC. The overall prevalence of VIA-positive was higher in this study than ~~in~~ a study ~~of women~~ at ~~the~~ Alameda Textile Factory in Tigray, which showed a VIA-positive rate of 6.7% [20]. A study at a Family Guidance Association of Ethiopia (FGAE) Clinic in Jimma, southern Ethiopia, showed that 12.9% of women were VIA-positive [19]. Studies in Swaziland, Cameroon, and Jakarta, Indonesia, showed VIA-positive rates of 15%, 3.33%, and 4.7%, respectively [10, 11, 12]. Our study also showed that 4.0% of women were suspicious for cancer compared to 1.2% of women in the study done in Jimma [19]. Though most are similar, the difference might be because of the skill difference of the care providers, and type acetic acid used for screening.

After adjusting for other variables, marital and educational status, occupation, history of STIs, ~~HIV status,~~ bleeding between period, and pelvic or back pain were statistically significant risk factors for VIA-positive or suspicious for cancer. Divorced or widowed women had ~~2.5 times the~~a risk of being VIA-positive (p=0.023) and ~~had 4.69 times the risk of~~ being suspicious (p=0.049) for CC than single women. In a study in Dar Es Salaam, Tanzania, divorced/separated women were ~~1.41 times~~ more likely to be VIA-positive [13]. In Jakarta, Indonesia, 8.3% of VIA-positive women were married more than once compared to 4.7% of overall women ~~overall~~ [12]. Women who have started sexual practice and have no permanent husband had a chance of

exposing to multiple sexual partner; which increases the risk of exposure to HPV. Increasing outreach and screening intensity to divorced, separated, and widowed women could be a simple approach to streamlining the identification and treatment of women at risk for developing CC.

Women in our study with primary education were approximately 70% less likely to be suspicious for CC (p=0.034) than women with no education, though education was not significantly correlated with VIA-positive. What's more, higher education levels, including secondary and tertiary, were not significantly associated with ~~lower rates of~~ VIA-positive or suspicious. In Dar Es Salaam, women with low education had odds 4.3~~0~~ higher of being VIA-positive [13]. Studies in Gondar, Ethiopia, and India showed that lack of awareness of CC was a risk factor for CC [7,16]. Increasing a~~A~~wareness including educational level could influence individuals to prevent themselves from any risk factors of cancer.

Women who are farmers and merchants had odds 4.8~~5~~ times higher of being suspicious for CC in our study. Farmers may have less awareness of CC and lack the ability to obtain early screening. In the case of merchants, these women are wealthier than farmers in the study area and may have heightened risk due to more ~~or riskier~~ sexual practices that expose them to HPV.

Respondents with a history of STI had 1.91 times the risk of being VIA-positive (p=0.018), though STIs were not statistically significantly associated with suspicious results. This was similar to studies done in Addis Ababa, Adama and at the Alamed Textile Factory where the history of STI was a risk factor for cervical precancerous ~~cervical~~ lesions [17, 18, 20]. One explanation for this is that women with STIs are more prone to HPV infection, which is by far the dominant pathway for CC to initiate and progress.

Bleeding between period and pelvic or back pain were significantly associated with suspicious results, ~~in our study. The odds of having a suspicious result~~which were 3.26 (p=0.010) and 2.79

(p=0.019) times higher~~, ~~ risk respectively~~, among women with bleeding between periods and pelvic/back pain~~than their counter parts. A study from Uganda revealed that the participants recognized inter-menstrual bleeding as a symptom of CC [9]. This is mainly related to suspicious for cancer that as the lesion advanced, the common sign and symptom are vaginal bleeding and pain.

Among women 46 years of age or older, 9.0% were found to be suspicious for CC ~~and 4.5% were VIA negative~~ compared to approximately 9.5% ~~and 3.3%, respectively,~~ for women less than 46 years of age. Though age was not statistically significant, further inquiry is needed to confirm and explain these variations. In a study in India, women 55 to 59 years of age had the highest risk of CC [8], this was also observed in one Ethiopian study [15]. ~~Indian~~ Ethiopian women may have ~~later~~ earlier sexual initiation than Indian women that delaying introduction and progression of the disease to pre-cancer lesion.

In the present study, the presence of cervical precancerous ~~cervical lesions~~ lesion was not associated with current contraceptive use or age at first sexual contact. ~~, the~~ The age at first sexual contact ~~latter of which~~ was significant predictor for cervical precancerous lesion in the study done in Jimma [19]. A study in Jakarta showed that current use of hormonal contraceptives was associated with ~~lower~~ odds of VIA-positive [12], whereas studies in Addis Ababa and Adama (Ethiopia), Uganda, and India showed that contraceptive use was a risk-factor for precancerous cervical lesions [9,8,17,18]. In Ethiopia, long time contraceptive users are few, which is evidenced by having high fertility rate. This may not predispose to cervical precancerious lesion.

Formatted: Space After: 10 pt, Adjust space between Latin and Asian text, Adjust space between Asian text and numbers

Formatted: Font: Calibri, 11 pt

Strenngth and limitation of the study:

The primary data of cervical precancerous lesion was acertaned using VIA screening, rather than history taking. This method of data collection minimizes recall bias. However a case control study design could better estimate the association of independent variables with the outcome variable.

Formatted: Font: 12 pt, Not Bold

Formatted: Font: 12 pt, Not Bold

Conclusion

In this study, 8.9% and 4% of the women were positive and suspicious for pre-cancer cervical lesion, respectively. Marital status, educational status, occupation, history of an STI, bleeding between period, and pelvic or back pain were risk factors for pre-cancer cervical lesion. Cervical cancer prevention is better to be strengthened focusing on preventable risk factors, including exposure to sexually transmitted infections and specific vulnerable groups like divorced and widowed women. Further study of the age distribution of precancerous lesions and suspicious results is merited.

List of Abbreviations

ARRR – Adjusted Relative Risk Ratio

cRRR - Crude Relative Risk Ratio

HIC - High Income Countries

HIV - Human Immunodeficiency Virus

HPV - Human Papilloma Virus

LMIC - Low Income Countries

RRR - Relative Risk Ration

STI - Sexually Transmitted Infection

SD - Standard Deviation

SCJ - Squamous-Columnar Junction

VIA - Visual Inspection With Acetic acid

Acknowledgments:

Our great appreciation goes to healthcare workers of the health facilities involved in the data collection and all study participants of the study. Moreover we would like to extend our acknowledgment for administrative and community health care workers for their support during data collection and facilitation of the project.

References

1. A report from WHO. A demonstration project, Prevention of cervical cancer through screening using visual inspection with acetic acid (VIA) and treatment with cryotherapy, African population and health research center, 2012.
2. American cancer society report, a guideline for cervical cancer and its managements, www.cancer.org Last Medical Review: 4/11/2013 and Last Revised: 1/31/2014
3. The Alliance for Cervical Cancer Prevention (ACCP), inspection with acetic acid (VIA): Evidence to date, www.alliance-cxca.org
4. Data derived from official reports by WHO, United Nations, Cervical Cancer Global Crisis Card, The World Bank, IARC Globocan http://www.who.int/hpvcentre/statistics/en/
5. Campbell C. Assessment of Cervical Cancer Incidence, Histopathology, and Screening Practices Among Hispanic Women in Latin America and Michigan, a dissertation for the degree of Doctor of Philosophy, 2011.
6. Zohre Momenimovahed and Hamid Salehiniya, Incidence, mortality and risk factors of cervical cancer in the world, Biomedical Research and Therapy, BioMedPress, 2018, http://www.bmrat.org/index.php/BMRAT/article/view/386
7. Rose I Anorlu, Cervical cancer: the sub-Saharan African perspective, Reproductive Health Matters, 2008;16(32)
8. Aswathy Sreedevi, Reshma Javed, and Avani Dinesh, Epidemiology of cervical cancer with special focus on India, Int J Womens Health. 2015; 7: 405–414, doi: 10.2147/IJWH.S50001

9. Amos D. Mwaka, Christopher G. Orach, Edward M. Were, Georgios Lyratzopoulos, Henry Wabinga, Martin Roland, Awareness of cervical cancer risk factors and symptoms: cross-sectional community survey in post-conflict northern Uganda, Health Expect. 2016 Aug; 19(4): 854–867, doi: 10.1111/hex.12382
10. Ginindza TG, Almonte M, Dlamini X, Sartorius B. Distribution of cervical abnormalities detected by visual inspection with acetic acid in Swaziland, 2011-2014: A retrospective study. *African J Prim Heal care Fam Med*. 2018;10(1):e1-e7. doi:10.4102/phcfm.v10i1.1773
11. Nkfusai NC, Mubah TM, Yankam BM, Tambe TA, Cumber SN. Prevalence of precancerous cervical lesions in women attending Mezam Polyclinic Bamenda, Cameroon. *Pan Afr Med J*. 2019;32:174. doi:10.11604/pamj.2019.32.174.16895
12. Anggraeni TD, Peters AAW. Prevalence , Age Distribution , and Risk Factors of Visual Inspection With Acetic Acid-Positive From 2007 to 2011 in Jakarta. 2017;22(2):103-107.
13. Kahesa C, Kjaer SK, Ngoma T, et al. Risk factors for VIA positivity and determinants of screening attendances in Dar es Salaam , Tanzania. *BMC Public Health*. 2012;12(1):1. doi:10.1186/1471-2458-12-1055
14. Haile T. Beyene M. Berg A. prevention of cervical cancer in Ethiopia, Senamoksh a report from Women´s Health Center, Addis Ababa, Ethiopia and ESOG, Ethiopia, a model clinic in Addis Ababa, 2018.
15. Dye D *et al*., 2009) Dye D., Solomon B., Claire H., Yared T., Vanessa H., Teshome D., Marion B., Anne R., **(2009**) Complex care systems in developing countries. *Breast Cancer Patient Navigation in Ethiopia* 116:577–85.)

16. Birhanu Z. et al, Health seeking behavior for cervical cancer in Ethiopia: a qualitative study, International Journal for Equity in Health, BioMed center, 2012, 11:83
17. Roza Teshome Kassa, Risk factors associated with precancerous cervical lesion among women screened at Marie Stops Ethiopia, Adama town, Ethiopia: a case control study, BMC Res Notes (2018) 11:145, https://doi.org/10.1186/s13104-018-3244-6.
18. Teame H, Addissie A, Ayele W, Hirpa S, Gebremariam A, Gebreheat G, et al. (2018) Factors associated with cervical precancerous lesions among women screened for cervical cancer in Addis Ababa, Ethiopia: A case control study. PLoS ONE 13(1): e0191506. https://doi.org/10.1371/journal.pone.0191506
19. Deksissa ZM, Tesfamichael FA, Ferede HA. Prevalence and factors associated with VIA positive result among clients screened at Family Guidance Association of Ethiopia, south west area office, Jimma model clinic, Jimma, Ethiopia 2013: A cross-sectional study Womens Health. *BMC Res Notes*. 2015;8(1):8-13. doi:10.1186/s13104-015-1594-x
20. Kebede Haile Misgina, Hailay Seyoum Belay and Teklehaymanot Huluf Abraha, Prevalence of precancerous cervical lesion and associated factors among women in North Ethiopia, Journal of Public Health and Epidemiology,2017, Vol. 9(3), pp. 46-50, DOI: 10.5897/JPHE2016.0891
21. Bayu H, Berhe Y, Mulat A, Alemu A (2016) Cervical Cancer Screening Service Uptake and Associated Factors among Age Eligible Women in Mekelle Zone, Northern Ethiopia, 2015: A Community Based Study Using Health Belief Model. PLoS ONE 11(3): e0149908. doi:10.1371/journal.pone.0149908
22. Yeneabeba Tilahun, National Cervical Cancer Prevention Training Package Participant Manual, Federal Ministry of Health Ethiopia, 2015.

23. Federal Democratic Republic of Ethiopia Ministry of Health. *Guideline for Cervical Cancer Prevention and Control in Ethiopia*.; 2015. https://www.iccp-portal.org/system/files/plans/Guideline Eth Final.pdf.

To: PLOSOne Editor-in-Chief
Subject: -Point by point response for the comments and recommendations of our reviewers
Title: "Epidemiology of precancerous cervical lesion and risk factors among adult women in Tigray, Ethiopia."
Reference: PONE-D-20-24267

Dear Editor-in-Chief
We are very grateful for the consideration of the manuscript. In accordance with the reviewers' valuable comments and recommendations, we have revised the manuscript and we hereby submit the revised work for your consideration.

Comments from the Editor:

1. Please ensure that your manuscript meets PLOS ONE's style requirements, including those for file naming. The PLOS ONE style templates can be found at

https://journals.plos.org/plosone/s/file?id=wjVg/PLOSOne_formatting_sample_main_body.pdf
and
https://journals.plos.org/plosone/s/file?id=ba62/PLOSOne_formatting_sample_title_authors_affiliations.pdf

- Response: This manuscript is prepared based on the information for author format from website of PLOSOne as much as possible

2. In your Methods section, please provide additional information about the participant recruitment method and the demographic details of your participants. Please ensure you have provided sufficient details to replicate the analyses such as: a) a description of any inclusion/exclusion criteria that were applied to participant recruitment, and b) a statement as to whether your sample can be considered representative of a larger population.

- Response: based on your valuable comment, we made clarification about the recruitment procedure, mentioned under the method section of the main document.
- The eligibility criteria specify who to include in the study. As mentioned under the multistage sampling technique, areas and participants were allocated proportionally and were selected using probability sampling method. We assume that this selection approach suffice the representativeness of the study participants.

3. Please provide additional details regarding participant consent. In the ethics statement in the Methods , please ensure that you have specified (1) whether consent was informed and (2) what type you obtained (for instance, written or verbal, and if verbal, how it was documented and witnessed).

    - Response: We accept the comment and written informed consent was obtained as mentioned under ethical consideration subsection of the method section.

4. You indicated that you had ethical approval for your study. In your Methods section, please ensure you have also stated whether you obtained consent from parents or guardians of the minors included in the study or whether the research ethics committee or IRB specifically waived the need for their consent.

- Responses: Since most participants were village dwellers, married and live independently, those above 15 years married and live independently were considered to sign the consent by their selves. This was mentioned in the protocol before obtaining the ethical clearance.

5. Please include additional information regarding the survey or questionnaire used in the study and ensure that you have provided sufficient details that others could replicate the analyses. For instance, if you developed a questionnaire as part of this study and it is not under a copyright more restrictive than CC-BY, please include a copy, in both the original language and English, as Supporting Information.

- Response: We agree with your concern and we can assure that we prepared the questionnaire from different literatures. We can submit our questionnaire up on request.

6. Please include a discussion of the limitations of your study in the Discussion section of your manuscript.

- Response: We accept the comment and correction was made in the main text of the discussion section.

Additional Editor Comments (if provided):
Editor's comments to the authors
Title: Epidemiology of precancerous cervical lesion and risk factors among adult women in Tigray, Ethiopia
1. In general, I agree that there are grammatical errors throughout the paper. I think the authors may find it helpful to consult an English Language Specialist or a native English Language speaker to edit the entire paper.

- Response: We accept the comment and we have received a copy edit of English language from English language professional.

2. The covering letter is not well structured. The authors should note that this is a ‘letter’ to the Editor; as such, it should be structured as a letter, addressed to the Editor, PLoS ONE, and signed off by the corresponding author. It should be clearly dated and state why the paper should be considered by the journal.

- Response: We accept the comment and we have made revision of the cover.

3. The authors should write in the language of research.
* “pre-cancer lesion” – should be written as ‘precancerous lesion’
* “Divorced or widowed women had risked 2.5 and 4.7 times more likely to be positive and

suspicious" – the language used: '…women had risked 2.5 and 4.7 times…' is not proper research language.
* "… were associated with suspicious for cancer" – associated with suspicious for cancer is not proper English
* "Data was 95 collected from March 2016 to June 2017". The word 'data' is plural; the authors should use 'were' after the word 'data'

- Response: We accept the comments above and we have made corrections in the abstract and pages 5 and 12 in the main txt.

4. The authors write: 'Data were collected using an interviewer administered questionnaire…' What data were actually collected? This is neither provided in the abstract nor in the main text of the paper.

- Response: The content of the questionnaire comprises information on socio-demographic and risk-factors, including health related behaviors, lifestyle, knowledge, attitude, and clinical attributes With due resection, we can assure that it has been mentioned under data collection section, in the previous version

5. The authors write, 'We added 10% to the calculated sample size to account for non-responses'. I don't think it is just 'adding' for the sake of adding. Can the authors explain why they adjusted the sample size by 10% and not any other percentage? Can they include a citation to back this up? How was the decision to adjust the sample size by 10% reached?

- Response: We accept that it needs justification. Sample size determination gives a minimum possible sample and maximizing sample size provides good representativeness, while more cot. After we determine the sample size, we assume that there will be non-respondents because of the cervical screening procedure using VIA. But we did not know how many of the recruited participants may refuse the procedure. Therefore 10% as compared to 5% would better compensate the number of non-respondents.

6. The authors should provide a little more detail on how the multistage sampling procedures were conducted. The information provided is not sufficient to explain what exactly happened at each stage.

- Response: We accept the comments and we have made corrections under the sampling technique section of the main text.

7. In Table 4, the authors should include n/N to guide interpretation of the findings from the bivariate and multivariable models. Besides, in principle, the primary outcome is not included in the table. So, I am surprised that Table 4 includes a column for 'VIA result'. In my view, this inclusion points to a serious error in the way the regression models were constructed.

- Response: The analysis model used is multinomial logistic regression with “Negative” result as base reference. Based on your valuable recommendation, we have made correction in the format of table 4.

8. The ARRR (95%CI) column within Table 4 is not well structured. The authors should ensure that all results are visible to the reader to aid interpretation.

- Response: Based on your valuable recommendation, we have made correction in the format of table 4.

9. The authors should ensure that all references are written in line with the journal’s referencing style and there should be consistency in the way all the references are presented.

- Response: We accept the comment and all published journals are written in Vancouver style as per the PLOSOne information for authors. Reference number 23 is used in the method section page 8.

[Note: HTML markup is below. Please do not edit.]

Reviewers' comments:

Reviewer's Responses to Questions

Comments to the Author

1. Is the manuscript technically sound, and do the data support the conclusions?

The manuscript must describe a technically sound piece of scientific research with data that supports the conclusions. Experiments must have been conducted rigorously, with appropriate controls, replication, and sample sizes. The conclusions must be drawn appropriately based on the data presented.

Reviewer #1: No

Reviewer #2: Partly

---

2. Has the statistical analysis been performed appropriately and rigorously?

Reviewer #1: No

Reviewer #2: I Don't Know

---

3. Have the authors made all data underlying the findings in their manuscript fully available?

The PLOS Data policy requires authors to make all data underlying the findings described in their manuscript fully available without restriction, with rare exception (please refer to the Data Availability Statement in the manuscript PDF file). The data should be provided as part of the manuscript or its supporting information, or deposited to a public repository. For example, in addition to summary statistics, the data points behind means, medians and variance measures should be available. If there are restrictions on publicly sharing data—e.g. participant privacy or use of data from a third party—those must be specified.

Reviewer #1: No

Reviewer #2: No

---

4. Is the manuscript presented in an intelligible fashion and written in standard English?

PLOS ONE does not copyedit accepted manuscripts, so the language in submitted articles must be clear, correct, and unambiguous. Any typographical or grammatical errors should be corrected at revision, so please note any specific errors here.

Reviewer #1: No

Reviewer #2: No

---

5. Review Comments to the Author

Please use the space provided to explain your answers to the questions above. You may also include additional comments for the author, including concerns about dual publication, research ethics, or publication ethics. (Please upload your review as an attachment if it exceeds 20,000 characters)

Reviewer #1: Abstract
1. Rewrite this sentence -Divorced or widowed women had risked 2.5 and 4.7 times more 35 likely to be positive for pre-cancer lesion and suspicious, respectively, compared to single women (rrr=2.5, 95% CI [1.13, 5.52]); (rrr=4.69, 95% CI [1.00, 21.84]).

- Response: We accept the comment and correction has been made under abstract and result sections.

2. How is the formal education suspicious result is (rrr=0.32 below zero , and 95% CI [1.00, 21.84]) is above one? Please justify it?

- Response: Dear/Sir/Madam, if we did not mistaken, rrr = 0.32 is for the Primary Education, while and 95% CI [1.00, 21.84] is for divorced or widowed variable. The C.I. for primary education variable with rrr = 0.32 is 0.32(0.11, 0.91), which does not include 1.

3. The associated risk factor is pre-cancer lesion or/and suspicious, please clear it.
generally -the sentence is incomplete and incorrect and grammatically wrong
the analysis is wrong .

- Response: We accept the comment and we have a concern on that VIA could not diagnose a cancer but it is good to understand that precancerous cervical lesion screening will come up with the results of negative, positive, and suspicious for cancer. In this sense, auspicious means not a cancer but pre-cancer till confirmed with diagnosis.

Reviewer #2: Summary: In their manuscript, Abera and colleagues present the findings of a study assessing the prevalence of precancerous cervical lesions and associated risk factors in Tigray, Ethiopia. Based on multistage sampling they recruited 900 women from the Tigray region, who then underwent visual inspection with acetic acid (VIA) and completed a questionnaire. The study question is interesting, as limited data on prevalence on cervical pre-cancer in Ethiopia are available. However, some aspects of the methodology and the results need to be clarified. Furthermore, the article would benefit from some language editing to improve comprehensibility.

Please find below specific comments for each section.

Data availability:

1. The authors state that the data are fully available and included in the manuscript. Is there an appendix with the individual patient data, or where can they be found?

- Response: only data in table 1, 2, and 3 were collected in this paper. Except the identifiers (like ID), there is no additional information collected than tables 1, 2, and 3. If the format of the empty questionnaire is needed, we can upload when requested.

1. In the background section the authors state that cervical cancer is the leading cause of mortality in women in LMIC. Are they referring to cancer-related mortality, or overall mortality?

- Response: We accept the comment and correction is made in the main text

2. The study aim was to assess the magnitude of precancerous lesions. May be better to use the term "prevalence" instead of "magnitude", as magnitude could also refer to the size of the lesions.

- Response: We accept the comment and correction is made in the main text

3. In the methods part the authors state that they estimated predictors. Predictors for what?

- Response: We accept the comment and it is to mention the predictors for cervical precancerous lesion.

4. The abbreviation rrr should be introduced.

- Response: The abbreviation rrr has been introduced in the “list of abbreviations” section.

5. In the conclusion, please state the implications of your findings.

- Response: We accept the comment and correction is made accordingly

Introduction:

1. Page 3, line 49: It’s important to highlight that CC is preventable only if treated early (not only detected early).

- Response: We accept the comment and correction is made under abstract and introduction sections

2. Page 3, line 52: Reference 5 does not fit that statement well. Rather cite Globocan?

- Response: We accept the comment and after checking the references, correction is made for the references

3. Page 3, line 54: Which host factors are the authors referring to?

- Response: We accept the comment and we refer the participants’ factor as the host factors.

4. Reference 14 seems incomplete. Can this report be found online?

- Response: We accept the comment and correction is made. It is an annual report filed as government document.

Methods:

1. Page 5, line 102: Why did the authors assume a cervical precancer prevalence of 50% for the sample size calculation when a previous institution-based study had found a prevalence of 6.7% in the Tigray region?

- Response: In Ethiopia, VIA screening was not available for non HIV women until 2015. It was only three hospitals providing VIA screening for HIV patents in the stufdy area, Tigray. Therefore the above mentioned prevalence is for HIV patients. Moreover, the above prevalence would provide us less number of participants and it is advisable to increases sample size if affordable. Therefore, we assume increasing participants would improve the representativeness of the eligible participants

.

2. What were the eligibility criteria for women to participate?

- Response: It was not elaborated earlier, that all adult women, who were sexually active, voluntarily consented to participate, and those did not undergo hysterectomy were the inclusion criteria, as now mentioned in the main text.

3. How did the systematic random sampling work? The process should be described in more detail. Under "data collection procedure" it sounds like a convenience sample from the selected sub-districts was used.
   - Response: Dear/Sir/Madam: about 3500 participants were estimated to attend the screening campaign (in fact 3866 were attending the screening campaign). Based on this source population, a key value of 4 was calculated (3500/900). Based on their arrival every forth was invited to participate in the study.
4. Please state which variables were included in the multivariable model finally.
   - Response: Dear/Sir/Madam: the model variables are mentioned under the "factors associated---," sub section, in paragraph two.

Results:

1. How did the authors determine the categorization of the variables, e.g. of parity (0-4. 5-12 children) and monthly income? Some of the categories contain few participants.
   - Response: Related to the parity, in Ethiopia fertility rate is 4.4; hence we categorized those below fertility rate and above fertility rate. But for the income category, though we could merge it, in the suspicious category it is still below 5% because of the suspicious cases are few. Therefore, if it is advisable, we think it will be better to remove up on recommendation.
2. How was history of STI determined? Symptoms? Treated STI?
   - Response: We determine the STI history based on items related to symptom of the infections taken from the national syndrome approach of the STI guideline.

3. Why was history of cervical cancer screening not included as a potential predictor? It would have been interesting to see how many women had been screened previously and whether that was associated with having cervical precancer.
   - Response: we respect your valuable comment. First of all, there was no routine cervical screening mechanism in the country; except for HIV cases started 2011. In the absence of service, asking utilization is not logical.

4. Table 4: Please report p-values for whole variables not individual categories of variables.
   - Response: We accept the concern, but this may not specify which category or level of a variable is associated with the outcome variable.

Discussion:

1. Page 12, line 232: HIV status mentioned here, but not in the results section?
   - Response: We accept the comment and correction is made accordingly
2. The authors should discuss the limitations of their study.
   - Response: We accept the comment and correction is made accordingly

3. The discussion is mainly a comparison with results from other studies. It would be helpful if the authors could also expand on hypotheses and explanations of why certain factors may predict cervical precancer. For example, why would divorced and widowed women be at increased risk of precancer after adjustment for age? Any hypotheses?
   - Response: We accept the comment and correction is made accordingly

---

6. PLOS authors have the option to publish the peer review history of their article (what does this mean?). If published, this will include your full peer review and any attached files.

If you choose "no", your identity will remain anonymous but your review may still be made public.

Do you want your identity to be public for this peer review? For information about this choice, including consent withdrawal, please see our Privacy Policy.

Reviewer #1: No

Reviewer #2: No

[NOTE: If reviewer comments were submitted as an attachment file, they will be attached to this email and accessible via the submission site. Please log into your account, locate the manuscript record, and check for the action link "View Attachments". If this link does not appear, there are no attachment files.]

While revising your submission, please upload your figure files to the Preflight Analysis and Conversion Engine (PACE) digital diagnostic tool, https://pacev2.apexcovantage.com/. PACE helps ensure that figures meet PLOS requirements. To use PACE, you must first register as a user. Registration is free. Then, login and navigate to the UPLOAD tab, where you will find detailed instructions on how to use the tool. If you encounter any issues or have any questions when using PACE, please email PLOS at figures@plos.org. Please note that Supporting Information files do not need this step.

**Date:**	February 12 2022
**To:**	PLOS ONE" plosone@plos.org
**From:**	Gerezgiher Buruh Abera" gbamsc2002@gmail.com (PI)
**Subject:**	Point by Point response to editor and reviewer(s).
**Title**	Epidemiology of Pre-cancerous Cervical Lesion and Risk Factors among Adult Women in Tigray, Ethiopia
**Reference**	PONE-D-20-24267R1

Dear/Sir/ professors: editor and reviewer:

We apologize for the late response! It is because of the internet lockdown in Tigray, North Ethiopia, due to crises. We are responding through the internet from NGOs that allow to use for publications, international projects and PhD students only. We are very grateful for the consideration of the manuscript. In accordance with the editors and reviewers' valuable comments and recommendations, we have revised the manuscript and we are hereby submitting the revised work for your consideration. Pages referred in this letter are based on the manuscript with track change.

Additional Editor Comments (if provided):

The authors have tried to address some of my comments but the paper still requires a lot of work before a decision can be made. I would like to request the authors to take time to think about the comments raised and try to offer appropriate responses to ALL the comments raised. We can't continue to return the same comments forever. So, the authors should look at the comments returned by reviewer No. 2 as well as myself and endeavor to fully address all the areas that were inadequately addressed. For instance, I raised issues on the grammatical errors which the authors indicated were addressed but to the contrary, the same errors as before still exist in the paper. I had a comment on the formatting of the references and requested the authors to revise them according to the journal's formatting requirements. However, the references were returned with the same errors as before. Besides, the explanation provided for adjusting the sample size by 10% is not sufficient. What is needed is to include a citation that backs up the use of this percentage. This was not done. Here are other additional comments for the authors to consider.

- Response*:* Sorry for the inconvenience! We were not to refuse comments. To be genuine, I am not competent in English, since it is my second language. In the previous time, we provide it to colleagues in Illinois University, a native English speaker (through the last listed co-author in this manuscript, She is from Chicago), and at that time we received the copy of the language edited version. Now we tried to go through the manuscript to correct grammatical errors as much as possible based on the direction given.
- We accept the comment on the format of the reference and we tried to correct especially reference 15 based on the old version of the paper, but we could not able to Google to recheck the citation style.
- A different contingency are commonly used from 5% to up to 30% (as in trials), based on the study design. In our cross sectional study, VIA screening was used, which is among the factors that could increase nonresponse rate. Now, we had added sample references to our manuscript. Based on previous studies, response rates ranges from 77.1% (24) to 98.2%, (25). This implies a non-response rate of 1.8% to 29.9% Therefore, based on the average of the references; we assume that 10% would enough to maximize the sample size to compensate the non-response rate expected.

1. The paper STILL has many grammatical errors that would benefit from being reviewed by an English Specialist. I raised similar comments earlier but these aspects are not yet fully addressed. For instance, within the abstract, instead of writing, '*Divorced or widowed women had risked 2.5 and 4.7 times more likely to be positive and suspicious, respectively, compared to single women…*', the authors should write: 'Divorced or widowed women were 2.5 and 4.7 times more likely to be positive and suspicious… than single women' The expressed: "had risked" is wrongly used. In addition, the use of 'more likely' begs the use of 'than' rather than 'compared to'. Similar statements exist in the abstract and in the main manuscript that should be addressed.

- Response - We accept the comments and it is corrected accordingly, as it can be seen in abstract, page 2 and result sections.

Please also fix the following errors/issues in the abstract:
*Expand 'rrr' at first mention, and then abbreviate thereafter
*The word 'In' in the title should be changed to 'in', while 'Among' should be written as 'among'.
*95%CI should be written in expanded form at first mention, and then abbreviate thereafter
'…68% lower for women with primary education compared to those with no formal education*' – use 'than' instead of 'compared to'.
'…History of sexually transmitted infection was associated with positive pre-cancer lesion*' – change 'pre-cancer lesion' to 'pre-cancerous cervical lesion'.
*'*Seventy-nine (8.95%) women were positive for pre-cancer lesion*' – "pre-cancer lesion" should be written as 'pre-cancerous cervical lesions'
'…were associated with suspicious for cancer*' – rewrite as: '… were associated with having a suspicious cervical cancer result'
*In the title, the authors refer to 'risk factors' but in the conclusion, they refer to 'predictors'. Do these two terms mean the same?

- Response: We accept the comment and rrr is written in expanded form (relative risk ratio) under the title of "data analysis" at last paragraph. In abstract, we prefer modification, to delete the values of regression including the rrr and represents using summarized statements. This minimizes the presentation of abstract and did not compromise the meaning, since it is already described under the result in detail.
- We accept the comment and the initial letter of 'In' 'and 'Among' in the title has been changed to small letters.
- We accept the comment and CI is written in expanded form (confidence interval) under the title of "Factors associated with cervical screening VIA result" at first paragraph of page 12.
- We accept the comment and the 'compared to' has been change to "than" as can be seen under the title of "Factors associated with cervical screening VIA result" at page 12, and under abstract.
- We accept the comment and the word "pre-cancerous cervical lesion' is used in the whole manuscript to replace all miss spelled "cervical precancerous lesion.
- We accept the comment and the meaning of 'risk factors' (something that contributes or indicates to illness: a features or habits, or personal history that increases the probability of disease) and 'predictors' (something explains what is going to happen in the future, often on the basis of present). We corrected as 'risk factors' as it can be seen in the conclusion section of the abstract and result titles.

2. In the abstract, the expression: '*A community-based cross sectional study was used…*' should be written as: 'This was a community-based, cross-sectional study conducted among 900 women in Tigray region, Ethiopia, from --- to ----'. In other words, the opening statement on the 'Methods' sub-section should tell the reader what the study design was, where the study was conducted, and when the study was done. Please note the use of the 'comma' after the word 'community-based' and the 'hyphen' inserted in the words 'cross sectional' to turn them into one word.

- Response: We accept the comment and corrected accordingly, as it can be seen in the abstract

3. General formatting requirements. The authors should ensure that they follow the general formatting requirements of the journal. For instance, the formatting for the sections indicates how the headings and sub-headings should be formatted, etc. For instance, under the 'Methods' section (the authors write it as 'Method'), this should be formatted as:

Materials and Methods (Level 1: bold type; 18pt font)

xxx

Study setting and design (Level 2: for sub-sections of major sections; Bold type, 16pt font)

- Response: We accept the comment and the titles and subtitles has been formatted as level 1 and level 2 respectively and Method has been changed to Materials and Methods.

4. Other corrections
*Use of abbreviations: Please ensure that ALL abbreviations are presented in expanded form at first mention in the abstract or body of the manuscript. Even if these abbreviations have been listed in the list of

abbreviations at the end of the paper, this does not take away the need to expand all abbreviations at first mention.

- Response: We accept the comments, and tried to correct accordingly through the whole manuscript.

*Line 153, page 7: '*Data was analysed…*' Data is a plural word; so, revise as: 'Data were analysed…'
Lines 163-164, page 8: the authors write: '…ethicalclearance was obtained from mekelle university, collage of health sience research and community service commiyyee*'. The word 'ethicalclearance' should be written as two words (ethical clearance). Names of places should always begin with a capital letter, e.g. Mekelle University… The word 'sience' should be edited to 'Sciences'. This also applies to 'collage' which should be written as 'College'. The word 'commiyyee' does not exist in English. I think the authors meant to write: 'committee'. In general, the entire paper should be edited to address any other areas that are not included in this report. The entire sentence should be revised as follows:
'… ethical clearance was obtained from Mekelle University College of Health Sciences Research and Community Service Committee …'

- Response: We accept the comments, and it is corrected accordingly under the 'data analysis and ethical consideration sub sections.

*Please note that should include the study protocol clearance numbers (e.g. Protocol#: 001/2019') as per your country's national research management guidelines.

- Response: We accept the comments, and the registration number has been included in the manuscript, under "Ethical consideration sub section.

*Ensure consistency in writing the word 'pre-cancerous'. In some sections of the paper, the word is written as 'precancerous' while in others, it is written as 'pre-cancerous'. Please choose one form and maintain it throughout the paper.

- Response: We accept the comments, and we tried to correct throughout the manuscript accordingly.

*Check ALL the references used against the journal's referencing guidelines. I can see that some journal names are italicized while others are not; I can also see that the referenced paper's title ends with a comma instead of a full stop. Other references have the journal name written in full. Please check the journal's referencing style (https://journals.plos.org/plosone/s/submission-guidelines) and fix all the errors in the reference section. When you click on the link, scroll down and look for 'references'. There is a table that shows you how different references should be written.

- Response: We accept the comments. As per our understanding, some journals specify citation preference with some abbreviations (example – BMC Res Notes is for BMC research Notes e.tc.), while others not, for those journals we took as it is. Because we may miss spell it when we abbreviate ourselves. The big challenge is that I am now in Tigray, North Ethiopia where the crisis found and all way of communication are locked. I am responding this Email through NGOs, which allow us to use internet for project and PhD publication only. I am sorry to say that I could not get internet access to either research the journals or to contact my most co-authors to recheck the reference (since they are living abroad). Anyway, I had tried to address some errors as I could.

*Reference 15 reads: '*Dye D et al., 2009) Dye D., Solomon B., Claire H., Yared T., Vanessa H., Teshome D., 384 Marion B., Anne R., (2009) Complex care systems in developing countries. Breast 385 Cancer Patient Navigation in Ethiopia 116:577–85.)*' This is an example of bad referencing style.

- Response: We accept the comments, and we tried to correct reference 15 in the revised manuscript by referring the old version.

In general, please ensure that the references are well formatted based on the journal style.
Please note that:
- Where there are more than six authors, please cite three of them followed by the word 'et al.'
- Where there are six authors or less, list all the authors.
- A reference cannot have two years of publication in the same reference, as shown in the example above
- The year of publication should come at the end, after the journal's name
- PLoS ONE does not use the author version (Anne R., or Marion B., ...). Click on the link above for guidance on how to write authors' names in the references section.

- PLoS ONE uses abbreviated journal names. Please ensure that the journal names are abbreviated but not italicized.

- Response: We accept the comments. For the name of authors in the references, it is difficult to differentiate the surname to be abbreviated, example - Amos D. Mwaka. We understand D. could be the first name. Therefore, when we use initial letter of last name first, we could mistake the first name rather than last name; for that, we prefer to use as it has been published in the journals.

5. Supplementary information. The journal requires that: "**Authors can submit essential supporting files and multimedia files along with their manuscripts. All supporting information will be subject to peer review. All file types can be submitted, but files must be smaller than 20 MB in size**." I did not see any supplementary information provided. Please click on the link above and check for 'supplementary information'. Here are examples of supplementary information that should be included:
*Data collection tools
*Dataset used during the analysis
* Please check the journal's guidelines on how supplementary information should be formatted.

- Response: We accept the comments, and we had uploaded the dataset in excel used during the analysis. There is no multimedia file which did not incorporated in the analysis and all of the relevant information is included in the manuscript. Data uploaded in the form of tables are used to analyze and develop the manuscript. For the confidentiality issue, we did not upload filled questionnaires.

6. Additional information requested at submission: All manuscripts should carry the following sub-section (please click on the link above for guidance on how to include this sub-section):
- Authors' contributions
[Note: HTML markup is below. Please do not edit.]

- Response: We accept the comments. Since it is short time allowed to use internet, we could not check the link to recheck requirements, but we used the link when we were preparing the manuscript for PLoS ONE. We understand that declaration is important and we added the declaration section as additional information including Authors' contributions and others at the end of this manuscript above acknowledgement sub section.

Reviewers' comments:
Reviewer's Responses to Questions
Comments to the Author

1. If the authors have adequately addressed your comments raised in a previous round of review and you feel that this manuscript is now acceptable for publication, you may indicate that here to bypass the "Comments to the Author" section, enter your conflict of interest statement in the "Confidential to Editor" section, and submit your "Accept" recommendation.

Reviewer #1: All comments have been addressed

Reviewer #2: (No Response)

---

2. Is the manuscript technically sound, and do the data support the conclusions?

The manuscript must describe a technically sound piece of scientific research with data that supports the conclusions. Experiments must have been conducted rigorously, with appropriate controls, replication, and sample sizes. The conclusions must be drawn appropriately based on the data presented.

Reviewer #1: Yes

Reviewer #2: No

3. Has the statistical analysis been performed appropriately and rigorously?

Reviewer #1: Yes

Reviewer #2: No

4. Have the authors made all data underlying the findings in their manuscript fully available?

The PLOS Data policy requires authors to make all data underlying the findings described in their manuscript fully available without restriction, with rare exception (please refer to the Data Availability Statement in the manuscript PDF file). The data should be provided as part of the manuscript or its supporting information, or deposited to a public repository. For example, in addition to summary statistics, the data points behind means, medians and variance measures should be available. If there are restrictions on publicly sharing data—e.g. participant privacy or use of data from a third party—those must be specified.

Reviewer #1: Yes

Reviewer #2: No

5. Is the manuscript presented in an intelligible fashion and written in standard English?

PLOS ONE does not copyedit accepted manuscripts, so the language in submitted articles must be clear, correct, and unambiguous. Any typographical or grammatical errors should be corrected at revision, so please note any specific errors here.

Reviewer #1: Yes

Reviewer #2: No

6. Review Comments to the Author

Please use the space provided to explain your answers to the questions above. You may also include additional comments for the author, including concerns about dual publication, research ethics, or publication ethics. (Please upload your review as an attachment if it exceeds 20,000 characters)

Reviewer #1: (No Response)

Reviewer #2: Summary: The comments have been incompletely addressed, and there are still numerous spelling/grammatical errors.

Please find below specific comments for each section.
Data availability:
1. Previous comment: The authors state that the data are fully available and included in the manuscript. Is there an appendix with the individual patient data, or where can they be found?
Author reply: only data in table 1, 2, and 3 were collected in this paper. Except the identifiers (like ID), there is no additional information collected than tables 1, 2, and 3.
If the format of the empty questionnaire is needed, we can upload when requested.
Comment: Data in table 1, 2, and 3 are aggregate data, not individual patient data. It is not possible to replicate the analyses with these data. Can the dataset with the individual patient data be access somewhere? If not, the data availability statement needs to be addressed accordingly.

- As per your good recommendation, the data availability sub section had been added in the manuscript under declaration section, describing all data are included in the manuscript.

Abstract:
1. Previous comment: The abbreviation rrr should be introduced.
Author reply: The abbreviation rrr has been introduced in the "list of abbreviations" section.
Comment: All abbreviations should be introduced in the manuscript text, not only in the list of abbreviations.

- Response: We accept the comment and rrr is written in expanded form (relative risk ratio) under the title of "data analysis" at last paragraph in its first use. In abstract, since the result section is better to be summary of the findings, we prefer to summarize and to delete the values of regression including the rrr. This could not change the meaning of the results.

2. Previous comment: In the conclusion, please state the implications of your findings.
Author reply: We accept the comment and correction is made accordingly.
Comment: What changes were made? I don't see any in the conclusion.

- Response: We accept the comment and implications for the prevalence and risk factors has been specified as seen under conclusion section.

Introduction:
1. Previous comment: Page 3, line 54: Which host factors are the authors referring to?
Author reply: We accept the comment and we refer the participants' factor as the host factors.
Comment: It is not enough to refer to participants' factors instead of host factors. Which participant factors may be relevant?

- Response: We understand your concern and we are referring to the life style (habits) of participants like risk taking behavior, substance abuse, alcohol use e.tc.

Methods:
1. Previous comment: Page 5, line 102: Why did the authors assume a cervical precancer prevalence of 50% for the sample size calculation when a previous institution-based study had found a prevalence of 6.7% in the Tigray region?
Author reply: In Ethiopia, VIA screening was not available for non HIV women until 2015. It was only three hospitals providing VIA screening for HIV patents in the study area, Tigray. Therefore the above mentioned prevalence is for HIV patients. Moreover, the above prevalence would provide us less number of participants and it is advisable to increases sample size if affordable. Therefore, we assume increasing participants would improve the representativeness of the eligible participants.
Comment: I do not understand this reply. Women living with HIV are at higher risk of developing precancerous cervical lesions. If the estimate of 6.7% stems from a study among women living with HIV, then a lower prevalence should be assumed for the reported study including HIV-negative women.

- Response: Yes, this is logically true. But to initiate our study, we base on a 4 years report from the regional health bureau and PATHFINDER, Ethiopia, Sep, 2014. In the report, 111 HIV unknown status were counseled and all were screened. Based on the result found, 29(26%) were VIA +ve for pre-cancerous cervical lesion with no specified reason. Therefore, we thought that it could be more and taking the 6.7% may provide less number of samples that could not represent the region and we decide to use if there is greater prevalence. We did not find any recent community based study in the region that could give us large sample size; for that reason we consider the prevalence that can provide us maximum sample size (50%). The drawback of this may be maximizing the cost of the study.

2. Previous comment: Please state which variables were included in the multivariable model finally.
Author reply: Dear/Sir/Madam: the model variables are mentioned under the "factors associated---," sub section, in paragraph two.
Response: From that paragraph it is not clear which variables were included in the final multivariable model. Furthermore, the authors should explain how they chose variables for inclusion in the multivariable model. This information should be given in the methods section.

- Response: We apologize for our previous response. As now included under the analysis sub section, the variables with p-value < 0.05 in the unadjusted multinomial regression was considered as significant and used in the multivariable multinomial logistic regression.

3. Eligibility criteria: "all adult women" – please state an age range. Were women eligible for screening from the age of 15 already? What do the national cervical cancer screening guidelines recommend?

- Response: We understand and accept the concern. When we depend on the national guideline, it recommends 30 – 49 years old female to be eligible for screening. This manual is developed based on different developing countries. Meaning, in our set up, there are 9 years old married (starting of sexual contact). The range of 30-49 ages implies that they started sex in the least 5 (immune-compromised) to 10 years (which is at 20 years to 44 years). In our set up, it is different, (which may be 14 - 19 years); for that we can find at least 15 to 19 years old married women stayed 5 to 10 yeast after sexual initiation on the ground. Moreover, this guideline is focusing on the HPV caused cervical cancer screening. But PHV is not the only factor that can cause cervical cancer. As part of our professional activities, we had faced with 16 years old women who had advanced cervical CA. This may be caused by other factors (not HPV) that are not related to early sexual initiation. Therefore, we consider 16 to 65 years old women who are with sexual experience (at least in the last 5 years) provided that they are living independent of their family or with husband for the justification of Ethical clearance. This provides a good coverage of eligible women in the community.

  Results:
  1. Previous comment: How was history of STI determined? Symptoms? Treated STI?
  Author response: We determine the STI history based on items related to symptom of the infections taken from the national syndrome approach of the STI guideline.
  Comment: This information should be added to the manuscript.

  - Response: We accept the comment and we used - having history of STI, if any one of the S/S is present (discharge, offensive secretion, itching, dysuria, lower abdominal pain, and fever). We also added it to the manuscript at the last statement of the data collection procedure sub section.

  2. Previous comment: Why was history of cervical cancer screening not included as a potential predictor? It would have been interesting to see how many women had been screened previously, and whether that was associated with having cervical precancer.
  Author response: we respect your valuable comment. First of all, there was no routine cervical screening mechanism in the country; except for HIV cases started 2011. In the absence of service, asking utilization is not logical.
  Comment: This background information should be added to the manuscript as readers may not be aware of this.
  - Response: We accept the concern, and we added it to the manuscript at the last statement of the data collection procedure sub section.

  3. Table 4: For some of the variables (e.g., age) there is no reference category for aRRR. The analysis looks wrong.
  - Response: We accept the concern, and 15-30 is the reference and we corrected it in the table (wrongly, last values of the category is pasted to first category in both positive and suspicious outcome variables).

  4. Table 4: explanations for *, **, and *** are missing.
  - Response: We accept your concern, it is included under the table as **NB:** P - value < 0.001 = ***, 0.001 - 0. 009 = ** and 0. 010 - 0. 05 = * and cRRR – crude relative risk ratio, aRRR - Adjusted relative risk ratio, C.I. - Confidence Interval

Discussion:

1. Previous comment: Page 12, line 232: HIV status mentioned here, but not in the results section?
Author response: We accept the comment and correction is made accordingly.
Comment: What is the correction? That HIV status is removed entirely? Does that mean that no information was available on HIV status?

- Response: At first, it was included as variable, and the statistician was used the raw data to analyze the draft result. But at that time when we evaluate the data collection process, we addressed that the

collected data on the variable 'HIV' status was incomplete (some sites has no HIV testing clinic and filled as unknown, which could not be logically true to treat those sites with sites that have HIV clinic). Hence we agreed to discard this variable to prevent error. The problem that we included it at the first draft was because of all Co-authors did not meet in person, since they live at different countries (Addis Ababa, Mekelle - Tigray, German, and Chicago (USA).

2. Previous comment: The authors should discuss the limitations of their study.
Author response: We accept the comment and correction is made accordingly.
Comment: Discussion of study limitations is insufficient.

- Response: We accept the concern, and we have tried to incorporate more limitations as much as we can. We believe limitations beyond the study may not be explanatory for our findings.

3. Previous comment: The discussion is mainly a comparison with results from other studies. It would be helpful if the authors could also expand on hypotheses and explanations of why certain factors may predict cervical pre-cancer. For example, why would divorced and widowed women be at increased risk of precancer after adjustment for age? Any hypotheses?
Author response: We accept the comment and correction is made accordingly.
Comment: This comment has been inadequately addressed, and the discussion section is difficult to understand sometimes. E.g., "In Ethiopia, long time contraceptive users are few, which is evidenced by having high fertility rate. This may not predispose to cervical pre-cancerious lesion." – what does that mean? What does not predispose to precancerous cervical lesions? The discussion section needs to be restructured and improved.

- Response: We accept the comment and tried to go through again to incorporate some hypothesis. It can be seen from track changes file.

---

7. PLOS authors have the option to publish the peer review history of their article (what does this mean?). If published, this will include your full peer review and any attached files.

If you choose "no", your identity will remain anonymous but your review may still be made public.

Do you want your identity to be public for this peer review? For information about this choice, including consent withdrawal, please see our Privacy Policy.

Reviewer #1: No

Reviewer #2: No

[NOTE: If reviewer comments were submitted as an attachment file, they will be attached to this email and accessible via the submission site. Please log into your account, locate the manuscript record, and check for the action link "View Attachments". If this link does not appear, there are no attachment files.]

While revising your submission, please upload your figure files to the Preflight Analysis and Conversion Engine (PACE) digital diagnostic tool, https://pacev2.apexcovantage.com/. PACE helps ensure that figures meet PLOS requirements. To use PACE, you must first register as a user. Registration is free. Then, login and navigate to the UPLOAD tab, where you will find detailed instructions on how to use the tool. If you encounter any issues or have any questions when using PACE, please email PLOS at figures@plos.org. Please note that Supporting Information files do not need this step.

In compliance with data protection regulations, you may request that we remove your personal registration details at any time. (Remove my information/details). Please contact the publication office if you have any questions.
