# Supplementary material for: Epidemiology of pre-cancerous cervical lesion and risk factors among adult women in Tigray, Ethiopia
Source: PLoS One. 2023 Jan 6;18(1):e0280191. doi: 10.1371/journal.pone.0280191 (PMC9821489; doi:10.1371/journal.pone.0280191)
Supplement: Attachment — Submitted filename: Rebuttal letter - Response for the reviewers.docx [file pone.0280191.s002.docx]

**To**: PLOSOne Editor-in-Chief

**Subject: -**Point by point response for the comments and recommendations of our reviewers

**Title:** “Epidemiology of precancerous cervical lesion and risk factors among adult women in Tigray, Ethiopia**."**

**Reference**: PONE-D-20-24267

Dear Editor-in-Chief

We are very grateful for the consideration of the manuscript. In accordance with the reviewers’ valuable comments and recommendations, we have revised the manuscript and we hereby submit the revised work for your consideration.

**Comments from the Editor:**

1. Please ensure that your manuscript meets PLOS ONE's style requirements, including those for file naming. The PLOS ONE style templates can be found at

<https://journals.plos.org/plosone/s/file?id=wjVg/PLOSOne_formatting_sample_main_body.pdf> and

<https://journals.plos.org/plosone/s/file?id=ba62/PLOSOne_formatting_sample_title_authors_affiliations.pdf>

- Response: This manuscript is prepared based on the information for author format from website of PLOSOne as much as possible

2. In your Methods section, please provide additional information about the participant recruitment method and the demographic details of your participants. Please ensure you have provided sufficient details to replicate the analyses such as: a) a description of any inclusion/exclusion criteria that were applied to participant recruitment, and b) a statement as to whether your sample can be considered representative of a larger population.

- Response: based on your valuable comment, we made clarification about the recruitment procedure, mentioned under the method section of the main document.
- The eligibility criteria specify who to include in the study. As mentioned under the multistage sampling technique, areas and participants were allocated proportionally and were selected using probability sampling method. We assume that this selection approach suffice the representativeness of the study participants.

3. Please provide additional details regarding participant consent. In the ethics statement in the Methods , please ensure that you have specified (1) whether consent was informed and (2) what type you obtained (for instance, written or verbal, and if verbal, how it was documented and witnessed).

- Response: We accept the comment and written informed consent was obtained as mentioned under ethical consideration subsection of the method section.

4. You indicated that you had ethical approval for your study. In your Methods section, please ensure you have also stated whether you obtained consent from parents or guardians of the minors included in the study or whether the research ethics committee or IRB specifically waived the need for their consent.

- Responses: Since most participants were village dwellers, married and live independently, those above 15 years married and live independently were considered to sign the consent by their selves. This was mentioned in the protocol before obtaining the ethical clearance.

5. Please include additional information regarding the survey or questionnaire used in the study and ensure that you have provided sufficient details that others could replicate the analyses. For instance, if you developed a questionnaire as part of this study and it is not under a copyright more restrictive than CC-BY, please include a copy, in both the original language and English, as Supporting Information.

- Response: We agree with your concern and we can assure that we prepared the questionnaire from different literatures. We can submit our questionnaire up on request.

6. Please include a discussion of the limitations of your study in the Discussion section of your manuscript.

- Response: We accept the comment and correction was made in the main text of the discussion section.

Additional Editor Comments (if provided):
Editor's comments to the authors
Title: Epidemiology of precancerous cervical lesion and risk factors among adult women in Tigray, Ethiopia
1. In general, I agree that there are grammatical errors throughout the paper. I think the authors may find it helpful to consult an English Language Specialist or a native English Language speaker to edit the entire paper.

- Response: We accept the comment and we have received a copy edit of English language from English language professional.

2. The covering letter is not well structured. The authors should note that this is a ‘letter’ to the Editor; as such, it should be structured as a letter, addressed to the Editor, PLoS ONE, and signed off by the corresponding author. It should be clearly dated and state why the paper should be considered by the journal.

- Response: We accept the comment and we have made revision of the cover.

3. The authors should write in the language of research.
* “pre-cancer lesion” – should be written as ‘precancerous lesion’
* “Divorced or widowed women had risked 2.5 and 4.7 times more likely to be positive and suspicious” – the language used: ‘…women had risked 2.5 and 4.7 times…’ is not proper research language.
* “… were associated with suspicious for cancer” – associated with suspicious for cancer is not proper English
* “Data was 95 collected from March 2016 to June 2017”. The word ‘data’ is plural; the authors should use ‘were’ after the word ‘data’

- Response: We accept the comments above and we have made corrections in the abstract and pages 5 and 12 in the main txt.

4. The authors write: ‘Data were collected using an interviewer administered questionnaire…’ What data were actually collected? This is neither provided in the abstract nor in the main text of the paper.

- Response: The content of the questionnaire comprises information on socio-demographic and risk-factors, including health related behaviors, lifestyle, knowledge, attitude, and clinical attributes With due resection, we can assure that it has been mentioned under data collection section, in the previous version

5. The authors write, ‘We added 10% to the calculated sample size to account for non-responses’. I don’t think it is just ‘adding’ for the sake of adding. Can the authors explain why they adjusted the sample size by 10% and not any other percentage? Can they include a citation to back this up? How was the decision to adjust the sample size by 10% reached?

- Response: We accept that it needs justification. Sample size determination gives a minimum possible sample and maximizing sample size provides good representativeness, while more cot. After we determine the sample size, we assume that there will be non-respondents because of the cervical screening procedure using VIA. But we did not know how many of the recruited participants may refuse the procedure. Therefore 10% as compared to 5% would better compensate the number of non-respondents.

6. The authors should provide a little more detail on how the multistage sampling procedures were conducted. The information provided is not sufficient to explain what exactly happened at each stage.

- Response: We accept the comments and we have made corrections under the sampling technique section of the main text.

7. In Table 4, the authors should include n/N to guide interpretation of the findings from the bivariate and multivariable models. Besides, in principle, the primary outcome is not included in the table. So, I am surprised that Table 4 includes a column for ‘VIA result’. In my view, this inclusion points to a serious error in the way the regression models were constructed.

- Response: The analysis model used is multinomial logistic regression with “Negative” result as base reference. Based on your valuable recommendation, we have made correction in the format of table 4.

8. The ARRR (95%CI) column within Table 4 is not well structured. The authors should ensure that all results are visible to the reader to aid interpretation.

- Response: Based on your valuable recommendation, we have made correction in the format of table 4.

9. The authors should ensure that all references are written in line with the journal’s referencing style and there should be consistency in the way all the references are presented.

- Response: We accept the comment and all published journals are written in Vancouver style as per the PLOSOne information for authors. Reference number 23 is used in the method section page 8.

[Note: HTML markup is below. Please do not edit.]

**Reviewers' comments:**
Reviewer's Responses to Questions

**Comments to the Author**
1. Is the manuscript technically sound, and do the data support the conclusions?
The manuscript must describe a technically sound piece of scientific research with data that supports the conclusions. Experiments must have been conducted rigorously, with appropriate controls, replication, and sample sizes. The conclusions must be drawn appropriately based on the data presented.

Reviewer #1: No

Reviewer #2: Partly

2. Has the statistical analysis been performed appropriately and rigorously?

Reviewer #1: No

Reviewer #2: I Don't Know

3. Have the authors made all data underlying the findings in their manuscript fully available?

The [PLOS Data policy](http://www.plosone.org/static/policies.action#sharing) requires authors to make all data underlying the findings described in their manuscript fully available without restriction, with rare exception (please refer to the Data Availability Statement in the manuscript PDF file). The data should be provided as part of the manuscript or its supporting information, or deposited to a public repository. For example, in addition to summary statistics, the data points behind means, medians and variance measures should be available. If there are restrictions on publicly sharing data—e.g. participant privacy or use of data from a third party—those must be specified.

Reviewer #1: No

Reviewer #2: No

4. Is the manuscript presented in an intelligible fashion and written in standard English?

PLOS ONE does not copyedit accepted manuscripts, so the language in submitted articles must be clear, correct, and unambiguous. Any typographical or grammatical errors should be corrected at revision, so please note any specific errors here.

Reviewer #1: No

Reviewer #2: No

5. Review Comments to the Author

Please use the space provided to explain your answers to the questions above. You may also include additional comments for the author, including concerns about dual publication, research ethics, or publication ethics. (Please upload your review as an attachment if it exceeds 20,000 characters)

**Reviewer #1: Abstract**
1. Rewrite this sentence -Divorced or widowed women had risked 2.5 and 4.7 times more 35 likely to be positive for pre-cancer lesion and suspicious, respectively, compared to single women (rrr=2.5, 95% CI [1.13, 5.52]); (rrr=4.69, 95% CI [1.00, 21.84]).

- Response: We accept the comment and correction has been made under abstract and result sections.

2. How is the formal education suspicious result is (rrr=0.32 below zero , and 95% CI [1.00, 21.84]) is above one? Please justify it?

- Response: Dear/Sir/Madam, if we did not mistaken, rrr = 0.32 is for the Primary Education, while and 95% CI [1.00, 21.84] is for divorced or widowed variable. The C.I. for primary education variable with rrr = 0.32 is 0.32(0.11, 0.91), which does not include 1.

3. The associated risk factor is pre-cancer lesion or/and suspicious, please clear it.
generally -the sentence is incomplete and incorrect and grammatically wrong
the analysis is wrong .

- Response: We accept the comment and we have a concern on that VIA could not diagnose a cancer but it is good to understand that precancerous cervical lesion screening will come up with the results of negative, positive, and suspicious for cancer. In this sense, auspicious means not a cancer but pre-cancer till confirmed with diagnosis.

Reviewer #2: Summary: In their manuscript, Abera and colleagues present the findings of a study assessing the prevalence of precancerous cervical lesions and associated risk factors in Tigray, Ethiopia. Based on multistage sampling they recruited 900 women from the Tigray region, who then underwent visual inspection with acetic acid (VIA) and completed a questionnaire. The study question is interesting, as limited data on prevalence on cervical pre-cancer in Ethiopia are available. However, some aspects of the methodology and the results need to be clarified. Furthermore, the article would benefit from some language editing to improve comprehensibility.
Please find below specific comments for each section.
Data availability:
1. The authors state that the data are fully available and included in the manuscript. Is there an appendix with the individual patient data, or where can they be found?

- Response: only data in table 1, 2, and 3 were collected in this paper. Except the identifiers (like ID), there is no additional information collected than tables 1, 2, and 3. If the format of the empty questionnaire is needed, we can upload when requested.

1. In the background section the authors state that cervical cancer is the leading cause of mortality in women in LMIC. Are they referring to cancer-related mortality, or overall mortality?

- Response: We accept the comment and correction is made in the main text

2. The study aim was to assess the magnitude of precancerous lesions. May be better to use the term “prevalence” instead of “magnitude”, as magnitude could also refer to the size of the lesions.

- Response: We accept the comment and correction is made in the main text

3. In the methods part the authors state that they estimated predictors. Predictors for what?

- Response: We accept the comment and it is to mention the predictors for cervical precancerous lesion.

4. The abbreviation rrr should be introduced.

- Response: The abbreviation rrr has been introduced in the “list of abbreviations” section.

5. In the conclusion, please state the implications of your findings.

- Response: We accept the comment and correction is made accordingly

Introduction:
1. Page 3, line 49: It’s important to highlight that CC is preventable only if treated early (not only detected early).

- Response: We accept the comment and correction is made under abstract and introduction sections

2. Page 3, line 52: Reference 5 does not fit that statement well. Rather cite Globocan?

- Response: We accept the comment and after checking the references, correction is made for the references

3. Page 3, line 54: Which host factors are the authors referring to?

- Response: We accept the comment and we refer the participants’ factor as the host factors.

4. Reference 14 seems incomplete. Can this report be found online?

- Response: We accept the comment and correction is made. It is an annual report filed as government document.

Methods:
1. Page 5, line 102: Why did the authors assume a cervical precancer prevalence of 50% for the sample size calculation when a previous institution-based study had found a prevalence of 6.7% in the Tigray region?

- Response: In Ethiopia, VIA screening was not available for non HIV women until 2015. It was only three hospitals providing VIA screening for HIV patents in the stufdy area, Tigray. Therefore the above mentioned prevalence is for HIV patients. Moreover, the above prevalence would provide us less number of participants and it is advisable to increases sample size if affordable. Therefore, we assume increasing participants would improve the representativeness of the eligible participants

.
2. What were the eligibility criteria for women to participate?

- Response: It was not elaborated earlier, that all adult women, who were sexually active, voluntarily consented to participate, and those did not undergo hysterectomy were the inclusion criteria, as now mentioned in the main text.

3. How did the systematic random sampling work? The process should be described in more detail. Under “data collection procedure” it sounds like a convenience sample from the selected sub-districts was used.

- Response: Dear/Sir/Madam: about 3500 participants were estimated to attend the screening campaign (in fact 3866 were attending the screening campaign). Based on this source population, a key value of 4 was calculated (3500/900). Based on their arrival every forth was invited to participate in the study.

4. Please state which variables were included in the multivariable model finally.

- Response: Dear/Sir/Madam: the model variables are mentioned under the “factors associated---,” sub section, in paragraph two.

Results:

1. How did the authors determine the categorization of the variables, e.g. of parity (0-4. 5-12 children) and monthly income? Some of the categories contain few participants.

- Response: Related to the parity, in Ethiopia fertility rate is 4.4; hence we categorized those below fertility rate and above fertility rate. But for the income category, though we could merge it, in the suspicious category it is still below 5% because of the suspicious cases are few. Therefore, if it is advisable, we think it will be better to remove up on recommendation.

2. How was history of STI determined? Symptoms? Treated STI?

- Response: We determine the STI history based on items related to symptom of the infections taken from the national syndrome approach of the STI guideline.

3. Why was history of cervical cancer screening not included as a potential predictor? It would have been interesting to see how many women had been screened previously and whether that was associated with having cervical precancer.

- Response: we respect your valuable comment. First of all, there was no routine cervical screening mechanism in the country; except for HIV cases started 2011. In the absence of service, asking utilization is not logical.

4. Table 4: Please report p-values for whole variables not individual categories of variables.

- Response: We accept the concern, but this may not specify which category or level of a variable is associated with the outcome variable.

Discussion:

1. Page 12, line 232: HIV status mentioned here, but not in the results section?

- Response: We accept the comment and correction is made accordingly

2. The authors should discuss the limitations of their study.

- Response: We accept the comment and correction is made accordingly

3. The discussion is mainly a comparison with results from other studies. It would be helpful if the authors could also expand on hypotheses and explanations of why certain factors may predict cervical precancer. For example, why would divorced and widowed women be at increased risk of precancer after adjustment for age? Any hypotheses?

- Response: We accept the comment and correction is made accordingly

6. PLOS authors have the option to publish the peer review history of their article ([what does this mean?](https://journals.plos.org/plosone/s/editorial-and-peer-review-process#loc-peer-review-history)). If published, this will include your full peer review and any attached files.
If you choose “no”, your identity will remain anonymous but your review may still be made public.
**Do you want your identity to be public for this peer review?** For information about this choice, including consent withdrawal, please see our [Privacy Policy](https://www.plos.org/privacy-policy).

Reviewer #1: No

Reviewer #2: No

[NOTE: If reviewer comments were submitted as an attachment file, they will be attached to this email and accessible via the submission site. Please log into your account, locate the manuscript record, and check for the action link "View Attachments". If this link does not appear, there are no attachment files.]
While revising your submission, please upload your figure files to the Preflight Analysis and Conversion Engine (PACE) digital diagnostic tool, <https://pacev2.apexcovantage.com/>. PACE helps ensure that figures meet PLOS requirements. To use PACE, you must first register as a user. Registration is free. Then, login and navigate to the UPLOAD tab, where you will find detailed instructions on how to use the tool. If you encounter any issues or have any questions when using PACE, please email PLOS at [figures@plos.org](mailto:figures@plos.org). Please note that Supporting Information files do not need this step.
