# Supplementary material for: Epidemiology of pre-cancerous cervical lesion and risk factors among adult women in Tigray, Ethiopia
Source: PLoS One. 2023 Jan 6;18(1):e0280191. doi: 10.1371/journal.pone.0280191 (PMC9821489; doi:10.1371/journal.pone.0280191)
Supplement: Attachment — Submitted filename: Response to Reviewers_February - 12 - 2022.docx [file pone.0280191.s003.docx]

| **Date:** | February 12 2022 |
| --- | --- |
| **To:** | PLOS ONE" plosone@plos.org |
| **From:** | Gerezgiher Buruh Abera" [gbamsc2002@gmail.com](mailto:gbamsc2002@gmail.com) (PI) |
| **Subject:** | Point by Point response to editor and reviewer(s). |
| **Title** | Epidemiology of Pre-cancerous Cervical Lesion and Risk Factors among Adult Women in Tigray, Ethiopia |
| **Reference** | PONE-D-20-24267R1 |

Dear/Sir/ professors: editor and reviewer:
We apologize for the late response! It is because of the internet lockdown in Tigray, North Ethiopia, due to crises. We are responding through the internet from NGOs that allow to use for publications, international projects and PhD students only. We are very grateful for the consideration of the manuscript. In accordance with the editors and reviewers’ valuable comments and recommendations, we have revised the manuscript and we are hereby submitting the revised work for your consideration. Pages referred in this letter are based on the manuscript with track change.

Additional Editor Comments (if provided):
The authors have tried to address some of my comments but the paper still requires a lot of work before a decision can be made. I would like to request the authors to take time to think about the comments raised and try to offer appropriate responses to ALL the comments raised. We can't continue to return the same comments forever. So, the authors should look at the comments returned by reviewer No. 2 as well as myself and endeavor to fully address all the areas that were inadequately addressed. For instance, I raised issues on the grammatical errors which the authors indicated were addressed but to the contrary, the same errors as before still exist in the paper. I had a comment on the formatting of the references and requested the authors to revise them according to the journal's formatting requirements. However, the references were returned with the same errors as before. Besides, the explanation provided for adjusting the sample size by 10% is not sufficient. What is needed is to include a citation that backs up the use of this percentage. This was not done. Here are other additional comments for the authors to consider.

- Response***:*** Sorry for the inconvenience! We were not to refuse comments. To be genuine, I am not competent in English, since it is my second language. In the previous time, we provide it to colleagues in Illinois University, a native English speaker (through the last listed co-author in this manuscript, She is from Chicago), and at that time we received the copy of the language edited version. Now we tried to go through the manuscript to correct grammatical errors as much as possible based on the direction given.
- We accept the comment on the format of the reference and we tried to correct especially reference 15 based on the old version of the paper, but we could not able to Google to recheck the citation style.
- A different contingency are commonly used from 5% to up to 30% (as in trials), based on the study design. In our cross sectional study, VIA screening was used, which is among the factors that could increase nonresponse rate. Now, we had added sample references to our manuscript. Based on previous studies, response rates ranges from 77.1% (24) to 98.2%, (25). This implies a non-response rate of 1.8% to 29.9% Therefore, based on the average of the references; we assume that 10% would enough to maximize the sample size to compensate the non-response rate expected.

1. The paper STILL has many grammatical errors that would benefit from being reviewed by an English Specialist. I raised similar comments earlier but these aspects are not yet fully addressed. For instance, within the abstract, instead of writing, ‘*Divorced or widowed women had risked 2.5 and 4.7 times more likely to be positive and suspicious, respectively, compared to single women…*’, the authors should write: ‘Divorced or widowed women were 2.5 and 4.7 times more likely to be positive and suspicious… than single women’ The expressed: “had risked” is wrongly used. In addition, the use of ‘more likely’ begs the use of ‘than’ rather than ‘compared to’. Similar statements exist in the abstract and in the main manuscript that should be addressed.

- Response - We accept the comments and it is corrected accordingly, as it can be seen in abstract, page 2 and result sections.

Please also fix the following errors/issues in the abstract:
*Expand ‘rrr’ at first mention, and then abbreviate thereafter
*The word ‘In’ in the title should be changed to ‘in’, while ‘Among’ should be written as ‘among’.
*95%CI should be written in expanded form at first mention, and then abbreviate thereafter
*’*…68% lower for women with primary education compared to those with no formal education*’ – use ‘than’ instead of ‘compared to’.
*’*…History of sexually transmitted infection was associated with positive pre-cancer lesion*’ – change ‘pre-cancer lesion’ to ‘pre-cancerous cervical lesion’.
*’*Seventy-nine (8.95%) women were positive for pre-cancer lesion*’ – “pre-cancer lesion” should be written as ‘pre-cancerous cervical lesions’
*’*…were associated with suspicious for cancer*’ – rewrite as: ‘… were associated with having a suspicious cervical cancer result’
*In the title, the authors refer to ‘risk factors’ but in the conclusion, they refer to ‘predictors’. Do these two terms mean the same?

- Response: We accept the comment and rrr is written in expanded form (relative risk ratio) under the title of “data analysis” at last paragraph. In abstract, we prefer modification, to delete the values of regression including the rrr and represents using summarized statements. This minimizes the presentation of abstract and did not compromise the meaning, since it is already described under the result in detail.
- We accept the comment and the initial letter of ‘In’ ‘and ‘Among’ in the title has been changed to small letters.
- We accept the comment and CI is written in expanded form (confidence interval) under the title of “Factors associated with cervical screening VIA result” at first paragraph of page 12.
- We accept the comment and the ‘compared to’ has been change to “than” as can be seen under the title of “Factors associated with cervical screening VIA result” at page 12, and under abstract.
- We accept the comment and the word “pre-cancerous cervical lesion’ is used in the whole manuscript to replace all miss spelled “cervical precancerous lesion.
- We accept the comment and the meaning of ‘risk factors’ (something that contributes or indicates to illness: a features or habits, or personal history that increases the probability of disease) and ‘predictors’ (something explains what is going to happen in the future, often on the basis of present). We corrected as ‘risk factors’ as it can be seen in the conclusion section of the abstract and result titles.

2. In the abstract, the expression: ‘*A community-based cross sectional study was used*…’ should be written as: ‘This was a community-based, cross-sectional study conducted among 900 women in Tigray region, Ethiopia, from --- to ----‘. In other words, the opening statement on the ‘Methods’ sub-section should tell the reader what the study design was, where the study was conducted, and when the study was done. Please note the use of the ‘comma’ after the word ‘community-based’ and the ‘hyphen’ inserted in the words ‘cross sectional’ to turn them into one word.

- Response: We accept the comment and corrected accordingly, as it can be seen in the abstract

3. General formatting requirements. The authors should ensure that they follow the general formatting requirements of the journal. For instance, the formatting for the sections indicates how the headings and sub-headings should be formatted, etc. For instance, under the ‘Methods’ section (the authors write it as ‘Method’), this should be formatted as:

**Materials and Methods** (Level 1: bold type; 18pt font)
xxx

**Study setting and design** (Level 2: for sub-sections of major sections; Bold type, 16pt font)

- Response: We accept the comment and the titles and subtitles has been formatted as level 1 and level 2 respectively and Method has been changed to Materials and Methods.

4. Other corrections
*Use of abbreviations: Please ensure that ALL abbreviations are presented in expanded form at first mention in the abstract or body of the manuscript. Even if these abbreviations have been listed in the list of abbreviations at the end of the paper, this does not take away the need to expand all abbreviations at first mention.

- Response: We accept the comments, and tried to correct accordingly through the whole manuscript.

*Line 153, page 7: ‘*Data was analysed*…’ Data is a plural word; so, revise as: ‘Data were analysed…’
*Lines 163-164, page 8: the authors write: ‘*…ethicalclearance was obtained from mekelle university, collage of health sience research and community service commiyyee*’. The word ‘ethicalclearance’ should be written as two words (ethical clearance). Names of places should always begin with a capital letter, e.g. Mekelle University… The word ‘sience’ should be edited to ‘Sciences’. This also applies to ‘collage’ which should be written as ‘College’. The word ‘commiyyee’ does not exist in English. I think the authors meant to write: ‘committee’. In general, the entire paper should be edited to address any other areas that are not included in this report. The entire sentence should be revised as follows:
‘**… ethical clearance was obtained from Mekelle University College of Health Sciences Research and Community Service Committee …**’

- Response: We accept the comments, and it is corrected accordingly under the ‘data analysis and ethical consideration sub sections.

*Please note that should include the study protocol clearance numbers (e.g. Protocol#: 001/2019’) as per your country’s national research management guidelines.

- Response: We accept the comments, and the registration number has been included in the manuscript, under “Ethical consideration sub section.

*Ensure consistency in writing the word ‘pre-cancerous’. In some sections of the paper, the word is written as ‘precancerous’ while in others, it is written as ‘pre-cancerous’. Please choose one form and maintain it throughout the paper.

- Response: We accept the comments, and we tried to correct throughout the manuscript accordingly.

*Check ALL the references used against the journal’s referencing guidelines. I can see that some journal names are italicized while others are not; I can also see that the referenced paper’s title ends with a comma instead of a full stop. Other references have the journal name written in full. Please check the journal’s referencing style [(https://journals.plos.org/plosone/s/submission-guidelines)](https://journals.plos.org/plosone/s/submission-guidelines) and fix all the errors in the reference section. When you click on the link, scroll down and look for ‘references’. There is a table that shows you how different references should be written.

- Response: We accept the comments. As per our understanding, some journals specify citation preference with some abbreviations (example – BMC Res Notes is for BMC research Notes e.tc.), while others not, for those journals we took as it is. Because we may miss spell it when we abbreviate ourselves. The big challenge is that I am now in Tigray, North Ethiopia where the crisis found and all way of communication are locked. I am responding this Email through NGOs, which allow us to use internet for project and PhD publication only. I am sorry to say that I could not get internet access to either research the journals or to contact my most co-authors to recheck the reference (since they are living abroad). Anyway, I had tried to address some errors as I could.

*Reference 15 reads: ‘*Dye D et al., 2009) Dye D., Solomon B., Claire H., Yared T., Vanessa H., Teshome D., 384 Marion B., Anne R., (2009) Complex care systems in developing countries. Breast 385 Cancer Patient Navigation in Ethiopia 116:577–85.)*’ This is an example of bad referencing style.

- Response: We accept the comments, and we tried to correct reference 15 in the revised manuscript by referring the old version.

In general, please ensure that the references are well formatted based on the journal style.

Please note that:
- Where there are more than six authors, please cite three of them followed by the word ‘et al.’
- Where there are six authors or less, list all the authors.
- A reference cannot have two years of publication in the same reference, as shown in the example above
- The year of publication should come at the end, after the journal’s name

- PLoS ONE does not use the author version (Anne R., or Marion B., ...). Click on the link above for guidance on how to write authors' names in the references section.
- PLoS ONE uses abbreviated journal names. Please ensure that the journal names are abbreviated but not italicized.

- Response: We accept the comments. For the name of authors in the references, it is difficult to differentiate the surname to be abbreviated, example - Amos D. Mwaka. We understand D. could be the first name. Therefore, when we use initial letter of last name first, we could mistake the first name rather than last name; for that, we prefer to use as it has been published in the journals.

5. Supplementary information. The journal requires that: “**Authors can submit essential supporting files and multimedia files along with their manuscripts. All supporting information will be subject to peer review. All file types can be submitted, but files must be smaller than 20 MB in size**.” I did not see any supplementary information provided. Please click on the link above and check for ‘supplementary information’. Here are examples of supplementary information that should be included:
*Data collection tools
*Dataset used during the analysis
* Please check the journal’s guidelines on how supplementary information should be formatted.

- Response: We accept the comments, and we had uploaded the dataset in excel used during the analysis. There is no multimedia file which did not incorporated in the analysis and all of the relevant information is included in the manuscript. Data uploaded in the form of tables are used to analyze and develop the manuscript. For the confidentiality issue, we did not upload filled questionnaires.

6. Additional information requested at submission: All manuscripts should carry the following sub-section (please click on the link above for guidance on how to include this sub-section):
- Authors’ contributions
[Note: HTML markup is below. Please do not edit.]

- Response: We accept the comments. Since it is short time allowed to use internet, we could not check the link to recheck requirements, but we used the link when we were preparing the manuscript for PLoS ONE. We understand that declaration is important and we added the declaration section as additional information including Authors’ contributions and others at the end of this manuscript above acknowledgement sub section.

**Reviewers' comments:**

Reviewer's Responses to Questions

**Comments to the Author**

1. If the authors have adequately addressed your comments raised in a previous round of review and you feel that this manuscript is now acceptable for publication, you may indicate that here to bypass the “Comments to the Author” section, enter your conflict of interest statement in the “Confidential to Editor” section, and submit your "Accept" recommendation.

Reviewer #1: All comments have been addressed

Reviewer #2: (No Response)

2. Is the manuscript technically sound, and do the data support the conclusions?

The manuscript must describe a technically sound piece of scientific research with data that supports the conclusions. Experiments must have been conducted rigorously, with appropriate controls, replication, and sample sizes. The conclusions must be drawn appropriately based on the data presented.

Reviewer #1: Yes

Reviewer #2: No

3. Has the statistical analysis been performed appropriately and rigorously?

Reviewer #1: Yes

Reviewer #2: No

4. Have the authors made all data underlying the findings in their manuscript fully available?

The [PLOS Data policy](http://www.plosone.org/static/policies.action#sharing) requires authors to make all data underlying the findings described in their manuscript fully available without restriction, with rare exception (please refer to the Data Availability Statement in the manuscript PDF file). The data should be provided as part of the manuscript or its supporting information, or deposited to a public repository. For example, in addition to summary statistics, the data points behind means, medians and variance measures should be available. If there are restrictions on publicly sharing data—e.g. participant privacy or use of data from a third party—those must be specified.

Reviewer #1: Yes

Reviewer #2: No

5. Is the manuscript presented in an intelligible fashion and written in standard English?

PLOS ONE does not copyedit accepted manuscripts, so the language in submitted articles must be clear, correct, and unambiguous. Any typographical or grammatical errors should be corrected at revision, so please note any specific errors here.

Reviewer #1: Yes

Reviewer #2: No

6. Review Comments to the Author

Please use the space provided to explain your answers to the questions above. You may also include additional comments for the author, including concerns about dual publication, research ethics, or publication ethics. (Please upload your review as an attachment if it exceeds 20,000 characters)

Reviewer #1: (No Response)

Reviewer #2: Summary: The comments have been incompletely addressed, and there are still numerous spelling/grammatical errors.

Please find below specific comments for each section.
Data availability:
1. Previous comment: The authors state that the data are fully available and included in the manuscript. Is there an appendix with the individual patient data, or where can they be found?
Author reply: only data in table 1, 2, and 3 were collected in this paper. Except the
identifiers (like ID), there is no additional information collected than tables 1, 2, and 3.
If the format of the empty questionnaire is needed, we can upload when requested.
Comment: Data in table 1, 2, and 3 are aggregate data, not individual patient data. It is not possible to replicate the analyses with these data. Can the dataset with the individual patient data be access somewhere? If not, the data availability statement needs to be addressed accordingly.

- As per your good recommendation, the data availability sub section had been added in the manuscript under declaration section, describing all data are included in the manuscript.

Abstract:
1. Previous comment: The abbreviation rrr should be introduced.
Author reply: The abbreviation rrr has been introduced in the “list of abbreviations”
section.
Comment: All abbreviations should be introduced in the manuscript text, not only in the list of abbreviations.

- Response: We accept the comment and rrr is written in expanded form (relative risk ratio) under the title of “data analysis” at last paragraph in its first use. In abstract, since the result section is better to be summary of the findings, we prefer to summarize and to delete the values of regression including the rrr. This could not change the meaning of the results.

2. Previous comment: In the conclusion, please state the implications of your findings.
Author reply: We accept the comment and correction is made accordingly.
Comment: What changes were made? I don’t see any in the conclusion.

- Response: We accept the comment and implications for the prevalence and risk factors has been specified as seen under conclusion section.

Introduction:
1. Previous comment: Page 3, line 54: Which host factors are the authors referring to?
Author reply: We accept the comment and we refer the participants’ factor as the host
factors.
Comment: It is not enough to refer to participants’ factors instead of host factors. Which participant factors may be relevant?

- Response: We understand your concern and we are referring to the life style (habits) of participants like risk taking behavior, substance abuse, alcohol use e.tc.

Methods:
1. Previous comment: Page 5, line 102: Why did the authors assume a cervical precancer prevalence of 50% for the sample size calculation when a previous institution-based study had found a prevalence of 6.7% in the Tigray region?
Author reply: In Ethiopia, VIA screening was not available for non HIV women until
2015. It was only three hospitals providing VIA screening for HIV patents in the study
area, Tigray. Therefore the above mentioned prevalence is for HIV patients. Moreover,
the above prevalence would provide us less number of participants and it is advisable
to increases sample size if affordable. Therefore, we assume increasing participants
would improve the representativeness of the eligible participants.
Comment: I do not understand this reply. Women living with HIV are at higher risk of developing precancerous cervical lesions. If the estimate of 6.7% stems from a study among women living with HIV, then a lower prevalence should be assumed for the reported study including HIV-negative women.

- Response: Yes, this is logically true. But to initiate our study, we base on a 4 years report from the regional health bureau and PATHFINDER, Ethiopia, Sep, 2014. In the report, 111 HIV unknown status were counseled and all were screened. Based on the result found, 29(26%) were VIA +ve for pre-cancerous cervical lesion with no specified reason. Therefore, we thought that it could be more and taking the 6.7% may provide less number of samples that could not represent the region and we decide to use if there is greater prevalence. We did not find any recent community based study in the region that could give us large sample size; for that reason we consider the prevalence that can provide us maximum sample size (50%). The drawback of this may be maximizing the cost of the study.

2. Previous comment: Please state which variables were included in the multivariable model finally.
Author reply: Dear/Sir/Madam: the model variables are mentioned under the “factors
associated---,” sub section, in paragraph two.
Response: From that paragraph it is not clear which variables were included in the final multivariable model. Furthermore, the authors should explain how they chose variables for inclusion in the multivariable model. This information should be given in the methods section.

- Response: We apologize for our previous response. As now included under the analysis sub section, the variables with p-value < 0.05 in the unadjusted multinomial regression was considered as significant and used in the multivariable multinomial logistic regression.

3. Eligibility criteria: “all adult women” – please state an age range. Were women eligible for screening from the age of 15 already? What do the national cervical cancer screening guidelines recommend?

- Response: We understand and accept the concern. When we depend on the national guideline, it recommends 30 – 49 years old female to be eligible for screening. This manual is developed based on different developing countries. Meaning, in our set up, there are 9 years old married (starting of sexual contact). The range of 30-49 ages implies that they started sex in the least 5 (immune-compromised) to 10 years (which is at 20 years to 44 years). In our set up, it is different, (which may be 14 - 19 years); for that we can find at least 15 to 19 years old married women stayed 5 to 10 yeast after sexual initiation on the ground. Moreover, this guideline is focusing on the HPV caused cervical cancer screening. But PHV is not the only factor that can cause cervical cancer. As part of our professional activities, we had faced with 16 years old women who had advanced cervical CA. This may be caused by other factors (not HPV) that are not related to early sexual initiation. Therefore, we consider 16 to 65 years old women who are with sexual experience (at least in the last 5 years) provided that they are living independent of their family or with husband for the justification of Ethical clearance. This provides a good coverage of eligible women in the community.

  Results:
  1. Previous comment: How was history of STI determined? Symptoms? Treated STI?
  Author response: We determine the STI history based on items related to symptom of the
  infections taken from the national syndrome approach of the STI guideline.
  Comment: This information should be added to the manuscript.
- Response: We accept the comment and we used - having history of STI, if any one of the S/S is present (discharge, offensive secretion, itching, dysuria, lower abdominal pain, and fever). We also added it to the manuscript at the last statement of the data collection procedure sub section.

2. Previous comment: Why was history of cervical cancer screening not included as a potential predictor? It would have been interesting to see how many women had been screened previously, and whether that was associated with having cervical precancer.
Author response: we respect your valuable comment. First of all, there was no routine
cervical screening mechanism in the country; except for HIV cases started 2011. In
the absence of service, asking utilization is not logical.
Comment: This background information should be added to the manuscript as readers may not be aware of this.

- Response: We accept the concern, and we added it to the manuscript at the last statement of the data collection procedure sub section.

3. Table 4: For some of the variables (e.g., age) there is no reference category for aRRR. The analysis looks wrong.

- Response: We accept the concern, and 15-30 is the reference and we corrected it in the table (wrongly, last values of the category is pasted to first category in both positive and suspicious outcome variables).

4. Table 4: explanations for *, **, and *** are missing.

- Response: We accept your concern, it is included under the table as **NB:** P - value < 0.001 = ***, 0.001 - 0. 009 = ** and 0. 010 - 0. 05 = * and cRRR – crude relative risk ratio, aRRR - Adjusted relative risk ratio, C.I. - Confidence Interval

Discussion:

1. Previous comment: Page 12, line 232: HIV status mentioned here, but not in the results section?
Author response: We accept the comment and correction is made accordingly.
Comment: What is the correction? That HIV status is removed entirely? Does that mean that no information was available on HIV status?

- Response: At first, it was included as variable, and the statistician was used the raw data to analyze the draft result. But at that time when we evaluate the data collection process, we addressed that the collected data on the variable ‘HIV’ status was incomplete (some sites has no HIV testing clinic and filled as unknown, which could not be logically true to treat those sites with sites that have HIV clinic). Hence we agreed to discard this variable to prevent error. The problem that we included it at the first draft was because of all Co-authors did not meet in person, since they live at different countries (Addis Ababa, Mekelle - Tigray, German, and Chicago (USA).

2. Previous comment: The authors should discuss the limitations of their study.
Author response: We accept the comment and correction is made accordingly.
Comment: Discussion of study limitations is insufficient.

- Response: We accept the concern, and we have tried to incorporate more limitations as much as we can. We believe limitations beyond the study may not be explanatory for our findings.

3. Previous comment: The discussion is mainly a comparison with results from other studies. It would be helpful if the authors could also expand on hypotheses and explanations of why certain factors may predict cervical pre-cancer. For example, why would divorced and widowed women be at increased risk of precancer after adjustment for age? Any hypotheses?
Author response: We accept the comment and correction is made accordingly.
Comment: This comment has been inadequately addressed, and the discussion section is difficult to understand sometimes. E.g., “In Ethiopia, long time contraceptive users are few, which is evidenced by having high fertility rate. This may not predispose to cervical pre-cancerious lesion.” – what does that mean? What does not predispose to precancerous cervical lesions? The discussion section needs to be restructured and improved.

- Response: We accept the comment and tried to go through again to incorporate some hypothesis. It can be seen from track changes file.

7. PLOS authors have the option to publish the peer review history of their article ([what does this mean?](https://journals.plos.org/plosone/s/editorial-and-peer-review-process#loc-peer-review-history)). If published, this will include your full peer review and any attached files.


If you choose “no”, your identity will remain anonymous but your review may still be made public.


**Do you want your identity to be public for this peer review?** For information about this choice, including consent withdrawal, please see our [Privacy Policy](https://www.plos.org/privacy-policy).

Reviewer #1: No

Reviewer #2: No

[NOTE: If reviewer comments were submitted as an attachment file, they will be attached to this email and accessible via the submission site. Please log into your account, locate the manuscript record, and check for the action link "View Attachments". If this link does not appear, there are no attachment files.]

While revising your submission, please upload your figure files to the Preflight Analysis and Conversion Engine (PACE) digital diagnostic tool, <https://pacev2.apexcovantage.com/>. PACE helps ensure that figures meet PLOS requirements. To use PACE, you must first register as a user. Registration is free. Then, login and navigate to the UPLOAD tab, where you will find detailed instructions on how to use the tool. If you encounter any issues or have any questions when using PACE, please email PLOS at [figures@plos.org](mailto:figures@plos.org). Please note that Supporting Information files do not need this step.

*In compliance with data protection regulations, you may request that we remove your personal registration details at any time.* [*(Remove my information/details)*](https://www.editorialmanager.com/pone/login.asp?a=r)*. Please contact the publication office if you have any questions.*
