# Supplementary material for: Epidemiology of pre-cancerous cervical lesion and risk factors among adult women in Tigray, Ethiopia
Source: PLoS One. 2023 Jan 6;18(1):e0280191. doi: 10.1371/journal.pone.0280191 (PMC9821489; doi:10.1371/journal.pone.0280191)
Supplement: Attachment — Submitted filename: Response to Reviewers_Sept - 11 - 2022.docx [file pone.0280191.s005.docx]

| **Date:** | September 14_2022 |
| --- | --- |
| **To:** | PLOS ONE" plosone@plos.org |
| **From:** | Gerezgiher Buruh Abera" [gbamsc2002@gmail.com](mailto:gbamsc2002@gmail.com) (PI) |
| **Subject:** | Point by Point response to editor and reviewer(s). |
| **Title** | Epidemiology of Cervical Precancerous Lesion and Risk Factors Among Adult Women In Tigray, Ethiopia |
| **Reference** | PONE-D-20-24267R2 |

Dear/Sir/ professors: editor and reviewers:
Thank you for your constructive and supportive comments. We are very grateful for the consideration of the manuscript. In accordance with the editors and reviewers’ valuable comments and recommendations, we have revised the manuscript and we are hereby submitting the revised work for your consideration.

**Reviewers' comments:**

**Reviewer's Responses to Questions**

1. If the authors have adequately addressed your comments raised in a previous round of review and you feel that this manuscript is now acceptable for publication, you may indicate that here to bypass the “Comments to the Author” section, enter your conflict of interest statement in the “Confidential to Editor” section, and submit your "Accept" recommendation.

Reviewer #3: (No Response)

Reviewer #4: (No Response)

________________________________________

2. Is the manuscript technically sounds, and do the data support the conclusions?

The manuscript must describe a technically sound piece of scientific research with data that supports the conclusions. Experiments must have been conducted rigorously, with appropriate controls, replication, and sample sizes. The conclusions must be drawn appropriately based on the data presented.

Reviewer #3: Yes

Reviewer #4: No

________________________________________

3. Has the statistical analysis been performed appropriately and rigorously?

Reviewer #3: Yes

Reviewer #4: I Don't Know

________________________________________

4. Have the authors made all data underlying the findings in their manuscript fully available?

The PLOS Data policy requires authors to make all data underlying the findings described in their manuscript fully available without restriction, with rare exception (please refer to the Data Availability Statement in the manuscript PDF file). The data should be provided as part of the manuscript or its supporting information, or deposited to a public repository. For example, in addition to summary statistics, the data points behind means, medians and variance measures should be available. If there are restrictions on publicly sharing data—e.g. participant privacy or use of data from a third party—those must be specified.

Reviewer #3: No

Reviewer #4: No

________________________________________

5. Is the manuscript presented in an intelligible fashion and written in Standard English?

PLOS ONE does not copyedit accepted manuscripts, so the language in submitted articles must be clear, correct, and unambiguous. Any typographical or grammatical errors should be corrected at revision, so please note any specific errors here.

Reviewer #3: Yes

Reviewer #4: No

________________________________________

**6. Review Comments to the Author**

Please use the space provided to explain your answers to the questions above. You may also include additional comments for the author, including concerns about dual publication, research ethics, or publication ethics. (Please upload your review as an attachment if it exceeds 20,000 characters)

**Reviewer #3:**

The most recent version is much better but still needs some improvement. So I have 4 main comments:

1. Yu need to go through the attached document and see all my highlighted arts and suggestions for improvement etc. then do work on them.

- Response: Thank you for detail comments on the attachment. We went through the attached pdf and tried to address comments highlighted. We accept all comments and that help us to made more correction in the manuscript.

2. Under the methods part what quality assurance measures were put in place apart from the training the nurses had? Did anyone else verify any screening findings?

- Response: We accept the comment and the provider was supported by the guideline protocol of VIA screening and supervised by gynecologists and assigned supervisors available in the institutions as appropriate to assure the quality of data collection. We include the quality steps in the main text as well.

3. I did not see any write up on what happened to screen positive women. You must always link screening to management so what happened?

- Response: We accept your comment and each client with positive or suspicious result was liked to related medical centers for further investigation and/or management. Most data collection sites have at least Cryotherapy for management and Gynecologic algorithm was used to follow clients with certain sign of pre-cancerous lesion. This is also added in the main text under the “Risk factors of cervical cancer:” subsection at page 11.

4. The limitations part, the key issue is the limitation of VIA for finding lesions, that is important and must be stated. And should then explain why that was used instead of more sensitive and specific screening modalities

- Response: We accept your comment and we added that other cervical screening are better identify pre-cancerous lesion, though the methods are not available in the study sites and are expensive than VIA.

**Reviewer #4:**

1. Previous comment: The abbreviation rrr should be introduced.

a. Author reply: The abbreviation rrr has been introduced in the “list of abbreviations” section.

b. Comment: All abbreviations should be introduced in the manuscript text, not only in the list of abbreviations.

c. •Response: We accept the comment and rrr is written in expanded form (relative risk ratio) under the title of “data analysis” at last paragraph in its first use. In abstract, since the result section is better to be summary of the findings, we prefer to summarize and to delete the values of regression including the rrr. This could not change the meaning of the results.

Comment on Revision 2: Results sections in the abstracts do normally contain the relative risk or adjusted relative risk. I would recommend including the numerical estimates, with the appropriate introduction of the abbreviation in the abstract.

- Response: We accept the comment and effects size estimations are included in the abstract with introduction of the rrr. We introduced the rrr in the abstract, under subsection “Factors associated with pre-cancerous Cervical Lesion) of page 12 and as footnote of table 4.

2. Previous comment: In the conclusion, please state the implications of your findings.

a. Author reply: We accept the comment and correction is made accordingly.

b. Comment: What changes were made? I don’t see any in the conclusion.

c. •Response: We accept the comment and implications for the prevalence and risk factors have been specified as seen under conclusion section.

Comment on Revision 2: I do not see any updates in the abstract’s conclusion section on implications of findings.

- Response: We accept the comment and sorry for that we missed correcting in the abstract, now we added the statement as “The prevalence of pre-cancerous cervical lesion is high as compared to other regional prevalence in the country. This finding implies that the sexual exposure, having no permanent husband and being not educated attributes to the high prevalence of pre-cancerous cervical lesion and may aggravate the transmission of HPV.”

Introduction:

1. Previous comment: Page 3, line 54: Which host factors are the authors referring to?

a. Author reply: We accept the comment and we refer the participants’ factor as the host factors.

b. Comment: It is not enough to refer to participants’ factors instead of host factors. Which participant factors may be relevant?

c. Response: We understand your concern and we are referring to the life style (habits) of participants like risk taking behavior, substance abuse, alcohol use e.tc. Methods: 1.

Comment on Revision 2: Substance abuse, alcohol use, risk-taking behavior are not risk factors for CC, except as they would work through increase in risk for HPV or HIV. I believe an appropriate way to address this comment would include the specifically identified risk factors which include immune status and smoking.

- Response: Dear/Sir, sorry for confusing you about our response statements, as per the Ethiopian cervical cancer prevention and control guideline, we tried to see if similar risk factors found in different researches. The ‘host factor” is mentioned in other studies as reference 5 and 6, but it is not ass variable of our study. There are many associated factors in different studies, example: risk taking behavior like sex without condom use, substance use including alcohol use or smoking and exposing to STI as risk factors for cervical cancer. Therefore we are referring to those factors as participant’s risk factors to unsafe sex and exposing to HPV, as HPV is prevalent STI. HPV is most common cause of cervical cancer. Hence those participants’ factors could have association with cervical cancer.

2. Previous comment: Page 5, line 102: Why did the authors assume a cervical pre-cancer prevalence of 50% for the sample size calculation when a previous institution-based study had found a prevalence of 6.7% in the Tigray region?

a. Author reply: In Ethiopia, VIA screening was not available for non HIV women until 2015. It was only three hospitals providing VIA screening for HIV patents in the study area previously, Tigray. Therefore the above mentioned prevalence is for HIV patients. Moreover, the above prevalence would provide us less number of participants and it is advisable to increases sample size if affordable. Therefore, we assume increasing participants would improve the representativeness of the eligible participants.

b. Comment: I do not understand this reply. Women living with HIV are at higher risk of developing precancerous cervical lesions. If the estimate of 6.7% stems from a study among women living with HIV, then a lower prevalence should be assumed for the reported study including HIV-negative women.

c. •Response: Yes, this is logically true. But to initiate our study, we base on a 4 years report from the regional health bureau and PATHFINDER Ethiopia, Sep, 2014. In the report, 111 HIV unknown status were counseled and all were screened. Based on the result found, 29(26%) were VIA +ve for pre-cancerous cervical lesion with no specified reason. Therefore, we thought that it could be more and taking the 6.7% may provide less number of samples that could not represent the region and we decide to use if there is greater prevalence. We did not find any recent community based study in the region that could give us large sample size; for that reason we consider the prevalence that can provide us maximum sample size (50%). The drawback of this may be maximizing the cost of the study.

d. Comment on Revision 2: I am confused by the reply. Overestimating the prevalence of precancer would decrease the overall sample size estimate for the study, making it more feasible, but also more likely to be underpowered to show associations. There are multiple studies from East Africa which show prevalence of VIA positivity, and most are within the rates of 10-30%. As the prior reviewer suggested, using the actual estimate of 6.7% would have facilitated a more appropriate sample size calculation.

- Response: We accept that it was good if the prevalence of cervical cancer among the HIV cases based screening to be used to calculate the sample size. The contextual differences between countries make us not to use other countries prevalence. We were standing on the fact that it could be good if all the communities included in the study, hence increasing participants would increase the power of the study. Because a prevalence cold give us minimum possible sample size, provided that maximizing is promoted. Besides, in the absence of prevalence among HIV unknown stats, in the study area, 50% is an alternative.

Three options are commonly used to determine sample size; the survey based, pre-existing study, and 50%. Therefore, at the conception of this project, we agreed to use 50%, as other alternative was not convenient. Being at this stage of the manuscript, we are happy if we get recommendation on the sample determination modification, but we believe it is difficult to modify the proportion size at this stage.

3. Previous comment: Please state which variables were included in the multivariable model finally.

a. Author reply: Dear/Sir/Madam: the model variables are mentioned under the “factors associated,” sub section, in paragraph two.

b. Response: From that paragraph it is not clear which variables were included in the final multivariable model. Furthermore, the authors should explain how they chose variables for inclusion in the multivariable model. This information should be given in the methods section.

c. •Response: We apologize for our previous response. As now included under the analysis sub section, the variables with p-value < 0.05 in the unadjusted multinomial regression was considered as significant and used in the multivariable multinomial logistic regression.

d. Comment on Revision 2: This has been partially addressed. Did the authors use any additional assessments of fit for the final regression model?

- Response: Variables like age, marital and education status, occupation, symptoms of cervical cancer, parity, STI history of partner, and lower abdominal pain were included in the multivariate analysis. Those variables were selected based on the p-value < 0.05 in the unadjusted multinomial regression. No other tests were used to select the model variables.

4. 3. Eligibility criteria: “all adult women” – please state an age range. Were women eligible for screening from the age of 15 already? What do the national cervical cancer screening guidelines recommend?

a. • Response: We understand and accept the concern. When we depend on the national guideline, it recommends 30 – 49 years old female to be eligible for screening. This manual is developed based on different developing countries. Meaning, in our set up, there are 9 years old married (starting of sexual contact). The range of 30-49 ages that they started sex in the least 5 (immune-compromised) to 10 years (which is at 20 years to 44 years). In our set up, it is different, (which may be 14 - 19 years); for that we can find at least 15 to 19 years old married women stayed 5 to 10 yeast after sexual initiation on the ground. Moreover, this guideline is focusing on the HPV caused cervical cancer screening. But PHV is not the only factor that can cause cervical cancer. As part of our professional activities, we had faced with 16 years old women who had advanced cervical CA. This may be caused by other factors (not HPV) that are not related to early sexual initiation. Therefore, we consider 16 to 65 years old women who are with sexual experience (at least in the last 5 years) provided that they are living independent of their family or with husband for the justification of Ethical clearance. This provides a good coverage of eligible women in the community.

b. Comment on Revision 2: I am concerned that the authors’ state screening women 15-19 or even younger would help identify non-HPV related cervical cancer. These numbers are vanishingly low (<0.01%), and multiple studies across many settings have shown that early screening, even in the setting of early sexual debut will have more harms, and costs, than benefits.

- Response: We accept your concern, but as per our senior gynecologists (member of the research) recommendation, we based on the community marital status and accepted as participants if they are married and had at least child, being in the age of 16 and above.

Results:

1. Previous comment: How was history of STI determined? Symptoms? Treated STI?

a. Author response: We determine the STI history based on items related to symptom of the infections taken from the national syndrome approach of the STI guideline.

Comment: This information should be added to the manuscript.

b. Response: We accept the comment and we used - having history of STI, if any one of the S/S is present (discharge, offensive secretion, itching, dysuria, lower abdominal pain, and fever). We also added it to the manuscript at the last statement of the data collection procedure sub section.

c. Comment on Revision 2: These was previously resolved

- Response: Thank you for helping us to address the above mentioned statement in the manuscript.

2. Previous comment: Why was history of cervical cancer screening not included as a potential predictor? It would have been interesting to see how many women had been screened previously, and whether that was associated with having cervical precancer.

a. Author response: we respect your valuable comment. First of all, there was no routine cervical screening mechanism in the country; except for HIV cases started 2011. In the absence of service, asking utilization is not logical.

b. Comment: This background information should be added to the manuscript as readers may not be aware of this.

c. •Response: We accept the concern, and we added it to the manuscript at the last statement of the data collection procedure sub section.

d. Comment on Revision 2: This has been addressed, but I also wonder why HIV status was not included as a risk factor?

- We accept the comment and the variable HIV status was included in our questionnaire, but excluded from analysis, because it was filled incomplete, since some sites has no HIV testing clinic and filled as unknown, which could not be logically true to treat those sites with sites that have HIV clinic. This has been added in the text at page 11 above table 2.

3. Table 4: For some of the variables (e.g., age) there is no reference category for aRRR. The analysis looks wrong.

a. •Response: We accept the concern, and 15-30 is the reference and we corrected it in the table (wrongly, last values of the category is pasted to first category in both positive and suspicious outcome variables).

b. This was addressed. Table 4 needs additional formatting to ensure visibility of complete variable names.

- Thank you for making us to see the table again, we tried to make the values visible by stretching the table space especially in the clean manuscript.

4. Table 4: explanations for *, **, and *** are missing.

•Response: We accept your concern, it is included under the table as NB: P - value < 0.001 = ***, 0.001 - 0. 009 = ** and 0. 010 - 0. 05 = * and cRRR – crude relative risk ratio, aRRR - Adjusted relative risk ratio, C.I. - Confidence Interval

a. Comment on Revision 2: This has been addressed.

- Thank you for confirmation of the response, your comment makes us to see our manuscript in different view.

Discussion:

1. Previous comment: Page 12, line 232: HIV status mentioned here, but not in the results section?

a. Author response: We accept the comment and correction is made accordingly.

b. Comment: What is the correction? That HIV status is removed entirely? Does that mean that no information was available on HIV status?

c. •Response: At first, it was included as variable, and the statistician was used the raw data to analyze the draft result. But at that time when we evaluate the data collection process, we addressed that the collected data on the variable ‘HIV’ status was incomplete (some sites has no HIV testing clinic and filled as unknown, which could not be logically true to treat those sites with sites that have HIV clinic). Hence we agreed to discard this variable to prevent error. The problem that we included it at the first draft was because of all Co-authors did not meet in person, since they live at different countries (Addis Ababa, Mekelle - Tigray, German, and Chicago (USA).

d. Comment on Revision 2: This absence and reason for the absence of this important variable needs to be addressed,

- Response: We accept the comment and we added the reason of removal of the “HIV status” variable under “risk factors of cervical cancer” subsection at last paragraph of page 11 above table 2, as follows “the variable HIV status was excluded from analysis, since it was filled incomplete, because some sites has no HIV testing clinic”, which is illogical to treat sites that has no HIV testing with those that has testing centers.

2. Previous comment: The authors should discuss the limitations of their study.

a. Author response: We accept the comment and correction is made accordingly.

b. Comment: Discussion of study limitations is insufficient.

c. •Response: We accept the concern, and we have tried to incorporate more limitations as much as we can. We believe limitations beyond the study may not be explanatory for our findings.

d. Comment on Revision 2: This is still underdeveloped. This would be a place to include limitations on data for follow-up (VIA is a proxy for pre-cancer and cancer), and lack of data on HIV status.

- Response: Thank you for helping us to see the limitation again. We tried to add the missed variables, used design and screening method other than VIA as limitation in the main text.

3. Previous comment: The discussion is mainly a comparison with results from other studies. It would be helpful if the authors could also expand on hypotheses and explanations of why certain factors may predict cervical pre-cancer. For example, why would divorced and widowed women be at increased risk of precancer after adjustment for age? Any hypotheses?

a. Author response: We accept the comment and correction is made accordingly.

Comment: This comment has been inadequately addressed, and the discussion section is difficult to understand sometimes. E.g., “In Ethiopia, long time contraceptive users are few, which is evidenced by having high fertility rate. This may not predispose to cervical pre-cancerious lesion.” – What does that mean? What does not predispose to precancerous cervical lesions? The discussion section needs to be restructured and improved.

b. •Response: We accept the comment and tried to go through again to incorporate some hypothesis. It can be seen from track changes file.

c. Comment on Revision 2: I feel like this comment has still not been appropriately addressed. The discussion still reads as a list of comparisons, rather than an exploration of the reasons for these differences. Major restructuring would improve this section. The conclusions sections does not make sense either—cervical cancer prevention through “preventable risk factors” is not as effective as through HPV testing and vaccination. I don’t think study of the age distribution of pre-cancerous lesions makes as much sense as study of the relationship between VIA and diagnosis of pre-cancer or cancer.

- We accept and agreed with your comments. We tried still to synthesis and justify the findings in the discussion part as can be seen from the track change of the manuscript. Or limitation is that we have no internet right now to find backgrounds of cited countries for justification and comparison of the other studies. It is a fact that HPV testing and vaccination is effective to prevent cervical cancer than doing on risk factors, but, it is not available in our setup and dealing with the absence of the service may not give meaning. The preventable risk factors could be in the capacity of poor countries like our country Ethiopia. In research findings, contraceptive use is mentioned as associated factors for cervical cancer. This could be because of the long term use of contraceptive example pills, expose them to STI, including HPV. This has been added to the main text

________________________________________

7. PLOS authors have the option to publish the peer review history of their article (what does this mean?). If published, this will include your full peer review and any attached files.

If you choose “no”, your identity will remain anonymous but your review may still be made public.

Do you want your identity to be public for this peer review? For information about this choice, including consent withdrawal, please see our Privacy Policy.

Reviewer #3: No

Reviewer #4: No

________________________________________

[NOTE: If reviewer comments were submitted as an attachment file, they will be attached to this email and accessible via the submission site. Please log into your account, locate the manuscript record, and check for the action link "View Attachments". If this link does not appear, there are no attachment files.]

While revising your submission, please upload your figure files to the Preflight Analysis and Conversion Engine (PACE) digital diagnostic tool, https://pacev2.apexcovantage.com/. PACE helps ensure that figures meet PLOS requirements. To use PACE, you must first register as a user. Registration is free. Then, login and navigate to the UPLOAD tab, where you will find detailed instructions on how to use the tool. If you encounter any issues or have any questions when using PACE, please email PLOS at figures@plos.org. Please note that Supporting Information files do not need this step.
